# Phytochemical Insights and Biological Potential of the *Helianthus* Genus

**DOI:** 10.3390/plants15030401

**Published:** 2026-01-28

**Authors:** Aldana Malen Corlatti, Hernán Bach, Ignacio Jorge Agudelo, Orlando Germán Elso, Rafael Ricco, Laura Cecilia Laurella, Valeria Patricia Sülsen

**Affiliations:** 1 Universidad de Buenos Aires, Facultad de Farmacia y Bioquímica, Cátedra de Farmacognosia, Junín 956 2° Floor, Buenos Aires 1113, Argentina; aldanamalencorlatti@gmail.com (A.M.C.); orlando.elso@gmail.com (O.G.E.); 2 CONICET–Universidad de Buenos Aires, Instituto de Química y Metabolismo del Fármaco (IQUIMEFA), Junín 956 2° Floor, Buenos Aires 1113, Argentina; 3 Instituto Nacional de Tecnología Agropecuaria, Nicolas Repetto y De los Reseros s/n, Buenos Aires B1686, Argentina; bach.hernan@inta.gob.ar; 4 Universidad de Buenos Aires, Facultad de Farmacia y Bioquímica, Museo de Farmacobotánica “Juan A. Dominguez”, Junín 956 1° Floor, Buenos Aires 1113, Argentina; 5 Universidad de Buenos Aires, Facultad de Farmacia y Bioquímica, Cátedra de Farmacobotánica, Junín 956 4° Floor, Buenos Aires 1113, Argentina; iagudelo@ffyb.uba.ar (I.J.A.); raricco@ffyb.uba.ar (R.R.)

**Keywords:** *Helianthus*, phenolic compounds, flavonoids, diterpenes, sesquiterpene lactones, biological activity

## Abstract

The *Helianthus* genus comprises more than 60 species distributed throughout North and Central America, with a few extending into South America. Among these, *H. annuus* and *H. tuberosus* represent the most widely utilized and extensively investigated species. The aim of this paper is to provide an overview of the current knowledge regarding the phytochemical composition and biological activities of *Helianthus* species. Phytochemical studies of *Helianthus* taxa have demonstrated that terpenoid constituents, including sesquiterpene lactones, diterpenes, and triterpenes, together with phenolic compounds, constitute the principal classes of secondary metabolites. Pharmacological investigations on *Helianthus* extracts have revealed a broad spectrum of biological activities. More than twenty distinct bioactivities have been reported for *H. annuus*, with the majority supported by in vitro assays (≈26 reports), reflecting multiple experimental evaluations per activity using different plant parts, extracts, and models; followed by a substantial number of in vivo studies in animal models (≈21 reports), and very limited clinical evidence. In comparison, five bioactivities have been described for *H. tuberosus*, mainly in vitro with a few in vivo reports, whereas only single in vitro bioactivities have been described for *H. salicifolius* and *H. angustifolius*. Among these, antidiabetic, antioxidant, antimicrobial, and anticancer properties are the most frequently documented.

## 1. Introduction

With more than 1600 genera and approximately 25,000 species distributed worldwide, the Asteraceae family, also known as Compositae, is one of the largest families of flowering plants [1,2]. Members of this family have been used since ancient times for both nutritional and medicinal purposes. Most species are valued for their therapeutic properties and have been widely employed in traditional medicine. They exhibit a broad spectrum of biological activities, including anti-inflammatory, antimicrobial, antioxidant, and hepatoprotective effects, among others [3]. The main phytochemical groups reported in Asteraceae include polyphenols, phenolic acids, flavonoids, acetylenes, triterpenes, and sesquiterpene lactones (STLs), which are considered responsible for many of their pharmacological properties [1].

Among the diverse members of the Asteraceae family, the tribe Heliantheae is one of the largest, comprising over 2000 species. Within this tribe, the genus *Helianthus*, classified under the subtribe Helianthinae, stands out due to its wide distribution across North America, Mexico, and South America [4]. Given the extensive diversity of the tribe and the genus, *Helianthus* species have been the focus of numerous studies on their ecological, economic, and pharmacological significance.

The *Helianthus* genus established by Linnaeus comprises between 51 and 67 recognized species, depending on the taxonomic framework adopted [5,6,7,8]. These plants are native to North America, with the highest species diversity found in the United States. Among them, the most widely recognized species is *Helianthus annuus* L., known as “Sunflower”, valued for its economic relevance and cultural significance.

Different criteria have been applied to the recognition of species within the genus *Helianthus* and to its infrageneric classification. As early as 1836, De Candolle proposed a system in which he recognized four groups. Subsequently, in 1842, Torrey and Gray divided the genus into six sections. During the 20th century, Watson separated it into two sections, whereas Heiser et al. classified it into three sections and seven series [5]. Later, Schilling and Heiser subdivided the genus into four sections: *Agrestes*, *Helianthus*, *Ciliares* (comprising two series), and *Divaricati* (comprising four series) [8]. All these proposals were based on morphological characteristics. Beginning in the 1990s, molecular studies provided new insights into the phylogeny of the group [9,10]. Annual species have been the most extensively studied, allowing the establishment of their affinities and even the elucidation of the hybrid origin of three species: *H. anomalus*, *H. deserticola*, and *H. paradoxus* [11,12,13,14,15,16,17].

Currently, the taxonomic classification of the genus *Helianthus*, according to the International Plant Names Index (IPNI) database, comprises 66 species. The species names and their geographical distributions are listed in Table 1.

Wild forms of *Helianthus annuus*, now widely cultivated, are common weeds across many parts of the United States. Other species within the genus have diverse distributions throughout the United States, Canada, and Mexico [18]. These species can be classified into three main groups: (a) shrubby species found in South America, (b) perennial species with rhizomes and tubers from North America (including *H. tuberosus* and various ornamental species), and (c) tap-rooted grassland perennials and annual plants, mostly found in the western regions of North America, with *H. annuus* being the most well-known annual species [19].

Some *Helianthus* species play prominent roles in natural vegetation, while several thrive as weeds in human-altered environments. Additionally, several species have been intentionally cultivated. Among these, *H. annuus* L. (common sunflower) and *H. tuberosus* L. (Jerusalem artichoke) are grown for their seeds and tubers, respectively, as food sources [7,20]. Numerous sunflower varieties are also cultivated for ornamental purposes, including *H. annuus*, *H. argophyllus* T. & G., and *H. debilis* Nutt. among the annuals, and *H. decapetalus* L., *H.* × *laetiflorus* Pers., *H. maximiliani* Schrad., *H.* × *multiflorus* L., and *H. salicifolius* A. Dietr. among the perennials [7]. Previous reviews on the genus have addressed its ethnobotanical and phytochemical characteristics [7,20,21,22]. However, in recent years, research has focused predominantly on *H. annuus* and its constituents. In this context, the present review aims to provide an updated overview of the genus *Helianthus*, emphasizing ethnobotanical, phytochemical, and pharmacological data published over the last decade. Special attention is given to studies on the chemical composition of *Helianthus* species, particularly terpenoid and phenolic compounds, as well as the biological activities of extracts and isolated constituents.

## 2. Methods

### Search Strategy and Selection Criteria

A literature search was conducted using three electronic databases: PubMed, Scopus, and Google Scholar. Relevant research articles and reviews addressing the phytochemistry, ethnobotany, pharmacology, and biological activities of *Helianthus* species were included. The search strategy combined the species names listed in Table 1, with the terms “biological activity,” “sesquiterpene lactones,” “terpenoid,” and “phenolic compounds”. For the review of ethnomedical uses, the terms “*Helianthus*” “ethnobotany” and “ethnomedical” were applied.

The general search string applied was: (species name) AND (biological activity OR sesquiterpene lactones OR terpenoid OR phenolic compounds). Each species’ name was queried independently using this structure. In PubMed and Scopus, additional Boolean operators were used to refine the searches by excluding studies focused exclusively on agronomic traits, crop production, and essential oils, in accordance with the predefined exclusion criteria. For Google Scholar, the same core search strategy was employed, with additional Boolean refinements applied to reduce irrelevant results, considering the platform’s limitations regarding advanced search syntax. In addition, an ethnobotanical search string was applied as follows: *Helianthus* AND (ethnobotany OR ethnomedical).

The search strategy for this review focused on academic sources published between 2014 and 2024, specifically studies addressing the biological activities of *Helianthus* species, as well as extracts, terpenoids, and phenolic compounds isolated from them. Some relevant earlier papers and reviews were also considered when they were not retrieved within the defined time frame but were considered essential for context.

All identified records were compiled, and duplicate entries were removed. Subsequently, titles and abstracts were screened based on predefined inclusion and exclusion criteria. Full-text articles were retrieved for records that passed the initial screening, and only those meeting all eligibility criteria were included in the final narrative synthesis. The inclusion criteria comprised: (i) publications addressing the phytochemistry, biological activity, and/or ethnobotanical or ethnomedical uses of *Helianthus* species; (ii) English-language articles; and (iii) original research articles and relevant reviews providing experimental or descriptive data. Exclusion criteria included: (i) studies dealing exclusively with agronomic traits or crop production; (ii) studies focused solely on essential oils without relevance to other phytochemical or pharmacological aspects; (iii) records without available full text; and (iv) duplicate publications. Emphasis was placed on aspects related to geographical distribution, phytochemistry, ethnobotany, and the pharmacological and biological activities of *Helianthus* species.

**Figure 1:**
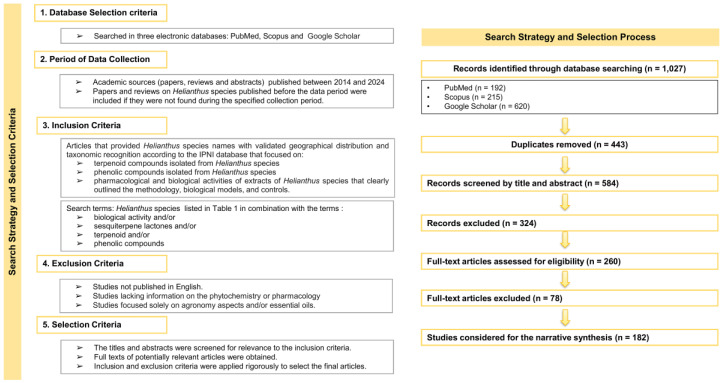
Flow diagram summarizing the literature search and selection process considered for the review.

The database search yielded a total of 1027 records, comprising 192 from PubMed, 215 from Scopus, and 620 from Google Scholar. After the removal of 443 duplicates, 584 records were screened by title and abstract, of which 324 were excluded for not meeting the selection criteria. A total of 260 full-text articles were assessed for eligibility, and 78 were excluded after full-text evaluation. Finally, 182 studies fulfilled the eligibility criteria and were included in the qualitative synthesis. Of these, a subset of the most relevant and representative publications was selected for explicit citation throughout the manuscript. The search strategy and selection process, including the number of records screened and included at each stage, are summarized in Figure 1.

The taxonomic classification and geographical distribution of *Helianthus* species were obtained from “Plants of the World Online” (https://powo.science.kew.org/ accessed on 20 May 2025) and the International Plant Names Index (IPNI; https://www.ipni.org accessed on 20 May 2025) and are summarized in Table 1.

Chemical structures of the compounds were generated using ChemOffice 2016 (ChemDraw Professional 2016, PerkinElmer, Waltham, MA, USA) and Microsoft Office (Microsoft Corp., Redmond, WA, USA), and verified against the PubChem database (https://pubchem.ncbi.nlm.nih.gov. accessed on 10 March 2025).

## 3. Ethnobotany of *Helianthus* spp.

Although the *Helianthus* genus comprises a wide range of species, *H. annuus* and *H. tuberosus* have been the most extensively used. Both species are employed nowadays all over the world, in a wide variety of geographical and cultural contexts. Therefore, there are many different uses for the same species according to the place and community that is using it.

Heiser was a pioneer in exploring the history and ethnobotany of the genus in North America, documenting the use of several *H. annuus* varieties and, less frequently, *H. petiolaris*. Among the reported uses, Dakota communities employed the flowers as a pectoral analgesic, while the Zuni used the same part of the plant as an antidote [23]. The earliest evidence of *H. annuus* domestication dates to 3000 years B.P. in the eastern United States, where numerous collections of achenes were found that differed from wild populations [24]. Additionally, archaeological records suggest the domestication of *H. annuus* in Mexico around 2600 B.C., with its use dating to 2900 B.P. This species had both nutritional and ceremonial significance, as its floral morphology symbolized solar deities in native cults. Regarding its contemporary uses in the same region, surveys conducted with indigenous communities in Mexico revealed that the main uses of sunflowers were for food and baking (achenes) and for ornamental purposes (flowers). However, some ethnic groups also highlight its medicinal properties, using it to treat stomach pain, as antitussive, and for rheumatism [25].

In Nuevo León, Mexico, the aerial parts of *H. annuus* have been used as forage by rural inhabitants [26]. Some documents from the 19th century likewise mention its aphrodisiac use and report headaches as a consequence of excessive consumption [27].

In South America, a decoction made from the achenes of *H. annuus* and the leaves of *Jodina rhombifolia* (Santalaceae) has been used to treat asthma [28]. In Brazil, Roma communities use the same part of the plant to treat hypercholesterolemia, hypertension, and cough [29]. It has also been employed as a vermifuge [30]. In the state of Pará, macerated sunflower seeds are consumed in alcoholic beverages to treat strokes, headaches, toothaches, and insect bites [31]. Similar uses for stroke have been reported for seeds consumed as infusions and decoctions in northeastern Brazil [32], and in Ceará, where they are also recommended to treat labyrinthitis [33]. Additionally, riverside populations in Mato Grosso use sunflower syrup to treat respiratory conditions [34]. In rural communities of Boyacá, Colombia, a decoction of the achenes and flowering aerial parts has been used as a digestive remedy for stomach inflammation [35].

*H. annuus* has also been used in Europe, Asia, and Africa. In Asturias, Spain, this species is grown for both food and ornamental purposes, while *Helianthus* × *laetiflorus* is cultivated only for decoration [36]. In Morocco, the seeds of *H. annuus* have been employed to treat cholesterol and circulatory pathologies [37]. In southern Kosovo, both Albanians and Gorani mix *H. annuus* seeds with animal fat to treat skin infections [38]. Reviews mention the use of this species in infusions and decoctions for the treatment of dermatitis and as an anti-stomachic [39]. In Ondo province, Nigeria, infusions of the leaves are used to treat respiratory infections [40].

*H. tuberosus* was introduced in Europe in the 16th century as food for humans and livestock. Due to its ability to spread through tubers and adapt to the European climate, it has been considered invasive in many countries [41]. Consequently, it has been widely used across Europe and Asia. In Turkey, for example, rural populations employ tubers to treat diabetes [42,43,44,45]. This use overlaps with its role as food, making it a functional food or nutraceutical. The same use has been reported in rural areas of southeastern Serbia [46], and it has been applied to relieve constipation (decoction) and as a salad by people in the Greek region of Macedonia [47]. The antidiabetic use has also been noted in the Lazio region [48], Emilia Romagna, and Malta [49]. In central Italy, it has been used as a galactagogue [50]. *H. tuberosus* is also consumed as food in Slovenia [51], by Albanians in southern Kosovo [38], and by locals in Armenia [52]. Interestingly, this species was introduced as forage for pig farming in Patagonia, Argentina [53].

References to the ethnobotanical uses of other *Helianthus* species are scarce. The sap of *Helianthus debilis* Nutt. is used to heal wounds in the Uttarakhand region of India [54].

It is important to keep in mind that there is no evidence based on clinical trials for these ethnomedical uses. However, it has been proven that *H. annuus* seed extracts rich in caffeoylquinic acids improved body fat loss and hyperlipidemia. These clinical trials validate the reported uses related to these diseases [55].

A comprehensive summary of ethnobotanical uses of *Helianthus* species, including plant parts used, traditional indications, preparation methods, cultural groups, and geographic distribution, is provided as Supplementary Materials (Table S1).

## 4. Phytochemistry of *Helianthus*

### 4.1. Terpenoid Compounds

Terpenoids are widely distributed among *Helianthus* species, predominantly comprising STLs, diterpenes, and triterpenes.

Given the recent comprehensive review by Galisteo et al. [56] on terpenoid diversity in *H. annuus*, this section provides only a brief overview of its phytochemistry [56].

The STLs in *Helianthus* have been widely studied by Spring and Schilling by employing the microsampling technique [21,57,58,59,60]. This method involves collecting 10 to 20 capitate glandular trichomes (GT) from the surface of the aerial parts of the plant and transferring them to a vial for extraction with organic solvent and further HPLC analysis. This technique, in contrast with leaf rinses with organic solvents, improves the purity of the sample in terms of STL content and allows the detection of those compounds that may be missed when processing bulk samples by traditional methods. In addition, microsampling techniques allow the detection of STLs in species lacking GT on leaves, by extraction and analysis of trichomes located in other parts of the plant [60].

**Figure 2:**
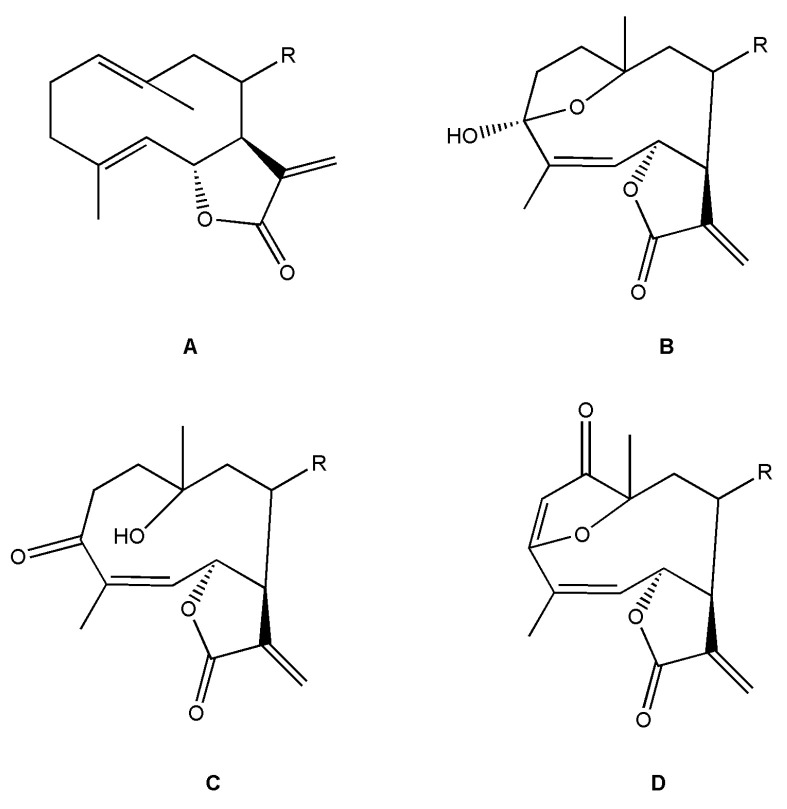
Most frequently germacranolide types present in *Helianthus*: germacrolides of the eupaserrin type (**A**) and heliangolides of the niveusin (**B**), tifruticin (**C**) and budlein types (**D**). Adapted from Spring and Schilling [21].

*Helianthus* species are rich sources of germacranolides, specifically germacrolides (*trans*,*trans*-1(10),4(5)-germacradienolides) and heliangolides (*trans*,*cis*-1(10),4(5)-germacradienolides) [22]. Among germacrolides, those belonging to the eupaserrin type and heliangolides of the niveusin, tifruticin and budlein types are frequently found (Figure 2). In contrast, germacranolides of the eupatolide and argophyllin types, 7,8-germacranolides, guaianolides and melampolides are only present in a minor number of species. Eudesmanolides, 6–6 bicyclic compounds based on the eudesmane skeleton, have also been isolated from *Helianthus* species [21,57,58,59,60].

Phytochemically, only a few studies have focused on the isolation of sesquiterpenes from *Helianthus* species other than *H. annuus* following the work of Spring. In this context, the study conducted by Kretschmer et al. can be highlighted, in which 8-isobutyryl, 8-isovaleryl-, and 8-methylbutyryl derivatives of 8-methacrylyl-4,15-iso-atropliciolide were reported from the flowers of *H. angustifolius* [61].

Subsequently, Yuan et al. isolated a new guaianolide, 3-hydroxy-8 β -tigloyloxy-1,10-dehydroariglovin, from the ethyl acetate extract of *H. tuberosus*, along with ten previously identified STLs. The known compounds identified were 4,15-iso-atripliciolide methacrylate, 4,15-iso-atripliciolide tiglate, 4,15-iso-atripliciolide isobutyrate, 4,15-iso-atripliciolide angelate, budlein A methacrylate, budlein A tiglate, desacetylovatifolin, 1α-hydroxypinnatifidin, 1α-acetoxypinnatifidin, and melampolide [62]. Later, Yuan et al. developed a HPLC-MS method for the simultaneous quantification of 11 STLs from *H. tuberosus*, allowing the detection of melampolide, 1α-acetoxypinnatifidin, and 4,15-iso-atripliciolide tiglate, among other previously reported compounds [63]. In 2019, Galkina et al. isolated a new furanoheliangolide (FHL) from the aerial parts of *H. tuberosus*, named heliantuberolide-8-O-tiglate [64]. In a later study, Saiki et al. isolated a STL identified as heliangin from the leaves of the same species known as Jerusalem artichoke [65].

Phytochemistry of *H. annuus* was extensively studied by Mascias et al. for the allelopathic potential of its terpenes, including the STLs [66,67,68,69,70]. Authors described the isolation of annuolides A-H, helivypolides A, B, D-F, H-J, helieudesmanolide A, and other STLs previously reported from aerial parts of *H. annuus*. A method for the isolation of bioactive compounds from *H. annuus* by supercritical carbon dioxide extraction has been described by El Marsni et al. [71]. In this study, the authors reported the isolation of several classes of phytochemicals, including the unreported STLs helivypolides K and L and helieudesmanolide B. The phytochemical profile of *H. annuus* was also investigated to assess the potential of its STLs in deterring the sunflower moth [72]. The study led to the isolation of argophyllone B, haageanolide, and several other previously reported STLs. More recently, Spring reported the presence of STLs in commercially available sunflower oils [73].

**Figure 3:**
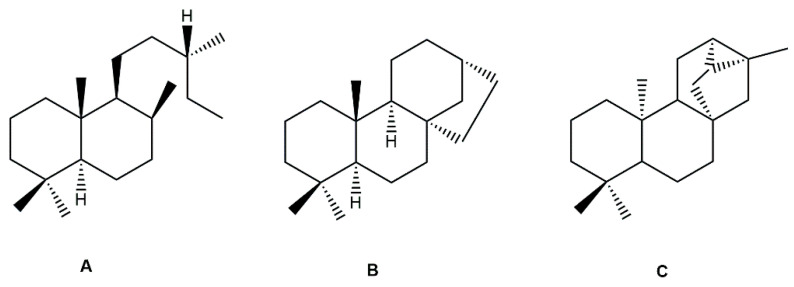
Diterpenes types frequently found in *Helianthus* species: labdane (**A**), kaurane (**B**) and trachylobane (**C**) types.

Diterpenes are another class of terpenoids widely distributed in *Helianthus* species. The main carbon skeletons identified belong to the labdane, kaurane, and trachylobane types (Figure 3). Diterpenes of the kaurane group are the most reported diterpenoids in *Helianthus*, being grandifloric acid, angeloylgrandifloric acid, ent-Kaur-16-en-19-oic acid and the latter’s 17-oxo and 17-hydroxy derivatives the major diterpenoids of this class in the genus. Diterpenes of the trachylobane, pimarane, labdane and atisane groups are also present in *Helianthus*, with ciliaric acid (trachylobanic skeleton) and ozic acid (labdane skeleton) the most distributed compounds of these types among *Helianthus* species. A dimer of a kaurenic acid and a trachylobanic acid was isolated from *H. radula*. More recently, Torres et al. reported the isolation of helikaurolides A-D from *H. annuus*, compounds with a structure that combines a sesquiterpene lactone and a kaurane diterpene [74].

Most of the diterpenes are carboxylic acids that are also found in other genera of Asteraceae, such as kaur-16-en-19-oic acid and 15-α-hydroxy-(-)-kaur-16-en-19-oic acid (grandifloric acid).

The presence of diterpenes has also been reported in economically valuable *Helianthus* species [22]. Among the diterpenes, 7-α-hydroxy-4-epitrachylobanoic acid was found in *H. petiolaris* Nutt. and *H. ciliaris* DC. Additionally, *ent*-12,16-cyclokaurenoic acid (also known as trachylobanoic acid) was identified in *H. debilis* Nutt., *H. giganteus* L., *H. hirsutus* Raf., *H. rigidus* Desf., and *H. tomentosus* Michx. *Ent*-kaurenoic acid has been reported in *H. decapetalus* Darl., *H. giganteus* L., *H. nuttallii* T. & G., and *H. rigidus* Desf. Furthermore, *ent*-12 β -acetoxykaurenoic acid was detected in *H. decapetalus* Darl., *H. decapetalus* var. *multiflorus*, *H. hirsutus* Raf., and *H. rigidus* Desf. *Ent*-13(S)-angeloxyatisenoic acid was described in *H. decapetalus* Darl. Gibberellins have been reported in *H. giganteus* L., while ent-9,11-didehydrokaurenoic acid was detected in *H. grosseserratus* Martins and *H. maximiliani* Schrad. Pimarane-type diterpenes have been identified in *H. hirsutus* Raf., and isocaurene derivatives were reported in *H. occidentalis* var. *dowellianus* T. & G.

Several triterpenes have been identified in *Helianthus* species, including triterpene alcohols similar to those reported in other yellow-flowered members of the Asteraceae family. Many of these compounds occur as glycosides. Triterpenes of the β-amyrin type, as well as oleanolic and albigenic acids, are commonly found.

Six triterpenoid glycosides, specifically known as helianthoside 1, helianthoside 2, helianthoside 3, helianthoside 4, helianthoside 5 and helianthoside B, were isolated from *H. annuus* [75,76].

Some of the most relevant terpenoid compounds isolated from *Helianthus* species are listed in Table 2.

The data summarized in Table 2 indicate that germacranolides represent the most widespread phytochemical group, being reported in 37 of the 47 species for which STLs have been described. These are followed by furanoheliangolides (27 species), heliangolides (19 species), and eudesmanolides (12 species). The least frequently occurring STL types are guaianolides, reported in *H. annuus*, *H. microcephalus*, and *H. tuberosus*; melampolides, identified in *H. tuberosus*; and furanogermacranolides, which have been reported in *H. niveus*.

**Figure 4:**
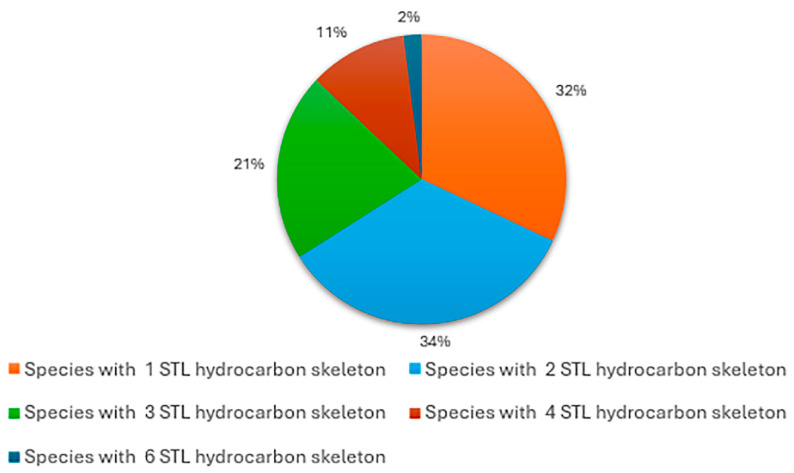
Expression of STL hydrocarbon skeletons in the *Helianthus* genus.

Analysis of skeletal diversity across the genus indicates that 15 species (32%) produce STLs with a single hydrocarbon skeleton, whereas 16 species (34%) and 10 species (21%) exhibit two and three distinct skeletal types, respectively. Higher skeletal diversity is less common, with five species (11%) expressing four different skeletons, and only one species, *H. tuberosus*, displaying six distinct STL skeletons (2%) (Figure 4).

Notably, two of the most widely used medicinally and economically important species within the genus, *H. annuus* and *H. tuberosus*, exhibit comparatively high STL skeletal diversity, with four and six skeletons, respectively. This chemical diversity may confer adaptive advantages by enabling these species to cope with a broad range of environmental conditions, herbivory pressures, and pathogen challenges. In addition, their rich and diverse STL profiles make them particularly suitable model systems for investigating the genetic and biochemical regulation of STL biosynthetic pathways.

No clear geographic pattern was evident in the distribution of STL skeletal types across the genus, as all major STL classes were represented across the different biomes occupied by these species. Nevertheless, comprehensive genus-wide metabolomic studies, ideally based on high-resolution mass spectrometry and specifically designed to address biogeographic variation, will be required to rigorously evaluate potential geographic structuring in STL diversity.

**Figure 5:**
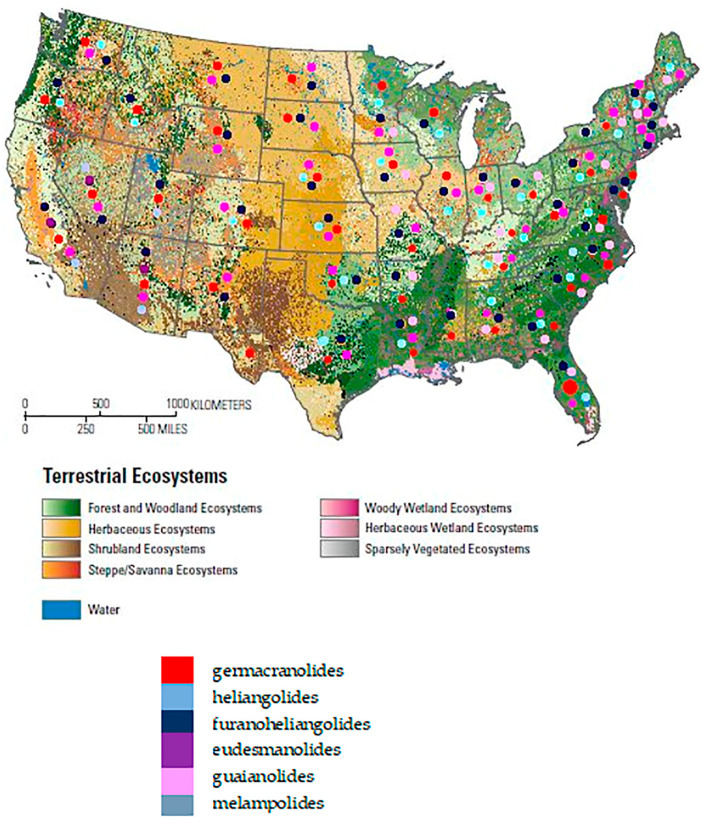
Geographic distribution of sesquiterpene lactone skeletal types reported in *Helianthus* species. Color code used to represent the different sesquiterpene lactone subclasses (filled circles on the map): germacranolides (red), heliangolides (light blue), furanoheliangolides (blue), eudesmanolides (violet), guaianolides (pink), and melampolides (gray).

To further contextualize this structural diversity, the geographic distribution of the main STLs skeletal types reported for *Helianthus* species was explored and is summarized in Figure 5.

Map adapted from USGS Publications Warehouse (https://pubs.usgs.gov/pp/1768/ accessed on 23 December 2025).

### 4.2. Flavonoids and Phenolic Compounds

The flavonoid chemistry of *Helianthus* has been previously reviewed by Bohm and Stuessy [79]. Flavones and flavonols represent the predominant flavonoid subclasses reported in the genus, mainly isolated from leaves. Among these, the aglycones 6-methoxy apigenin, 6,4-dimethoxy apigenin, and 6-methoxy luteolin, as well as the glycosides of kaempferol and quercetin, are the most reported flavonoids in *Helianthus*. The flavones hymenoxyn and nevadensin have also been reported in several species of the genus. The yellow pigment coreopsin (chalcone) and sulfuretin (aurone) have been isolated from flowers of *Helianthus* species.

The flavonoid composition of *Helianthus* species of economic relevance has been evaluated by Tosun et al. [22]. Among the documented compounds, nevadensin was detected in *H. annuus* L., *H. angustifolius* L., *H. floridanus* A. Gray, *H. microcephalus* T. & G., *H. simulans*, and *H. strumosus* L. The aurone 5-hydroxy-4,6,4′-trimethoxyaurone was described in *H. annuus* L. [22,79], while the flavone hymenoxin was reported in *H. angustifolius* L., *H. floridanus* A. Gray, *H. microcephalus* T. & G., *H. simulans*, and *H. strumosus* L. Additionally, hispidulin was identified in *H. angustifolius* L., *H. floridanus* A. Gray, *H. microcephalus* T. & G. and *H. simulans*. Nepetin and jaceosidin were also described in these species [22].

More recently, Kaszás et al. characterized the biochemical composition of leaf protein concentrate (JAPC) obtained from fresh aerial biomass of *H. tuberosus* using UHPLC-ESI-ORBITRAP-MS/MS. This analysis revealed the presence of isorhamnetin-3-O-glucoside, kaempferol 3-glucuronide (kaempferol 3-O-β-D-glucopyranosiduronic acid), and astragaline (kaempferol 3-O-β-D-glucopyranoside). In addition, glucuronide derivatives of isorhamnetin (isorhamnetin-3-O-glucuronide) and isoquercetin (quercetin 3-O-β-d-glucopyranoside) were identified for the first time in JAPC, along with several dimethoxy- and trihydroxyflavone derivatives, including two dimethoxy-trihydroxyflavone isomers, a dimethoxy-tetrahydroxyflavone, a dihydroxy-methoxyflavone, and a trihydroxy-trimethoxyflavone. The flavones nevadensin and hymenoxin were also detected. Among chalcones, butein (2′,3,4,4′-tetrahydroxychalcone) and kukulkanin B (3′-methoxy-2′,4,4′-trimethoxychalcone) were reported, whereas liquiritigenin (4′,7-dihydroxy flavanone) was the only flavanone identified [80].

Similarly, Wang et al. identified a wide range of bioactive compounds in the aerial parts of *H. tuberosus*, including the flavonoids nevadensin, hymenoxin, kaempferol gluconate, kaempferol, kaempferol-3-O-glucoside, rutin, andrographin, nobiletin, silymarin, puerarin, and rhamnazin, as well as the phenolic acids 3,4-dicaffeoylquinic acid, 3-feruloylquinic acid, chlorogenic acid, 1,5-dicaffeoylquinic acid, catechin, salicylic acid, epigallocatechin gallate, and p-coumaroylquinic acid [81].

The chalcone coreopsin was reported in *H. angustifolius* L., *H. floridanus* A. Gray., *H. heterophyllus* Nutt., and *H. longifolius* Pursh [22]. Additionally, its presence was documented in *H. atrorubens*, *H. glaucophyllus*, and *H. gracilentus* by Bohm and Stuessy [79]. The aurone sulfuretin was initially described in *H. angustifolius* L., *H. floridanus* A. Gray., *H. heterophyllus* Nutt., and *H. longifolius* Pursh [22], and was later documented in *H. atrorubens*, *H. glaucophyllus*, and *H. gracilentus* by Bohm and Stuessy [79]. These authors also reported the presence of 5-hydroxy-7,4′-dimethoxyflavan in *H. microcephalus*, as well as the flavonol fisetin in *H. gracilentus*. Several additional flavonoids were also characterized in this species, including chalcones such as 2′,4′,4-trihydroxy-3′-methoxychalcone and heliannone A, the flavanone heliannone C, and the flavonol tambulin [79].

Spring et al. isolated the flavones gardenin B, methylsudachitin, desmethylsudachitin, acerosin, sideritiflavone and 5-deoxy-flavenone from linear GT of *H. annuus* [82]. Silva et al. applied UPLC-DAD-MS and developed a mass spectrometry imaging method for direct analysis of secondary metabolites from trichomes of *H. annuus* and, through comparison with authentic standards and UV/MS data, detected the flavone demethoxysudachitin, nevadensin, acerosin and xanthomicrol among other previously reported flavonoids [83].

Other studies have reported the isolation of additional flavonoids from *H. tuberosus.* Chae et al. isolated the glycosides Kaempferol 3-O-glucoside and quercetin 7-O-glucoside, while Yuan et al. reported the flavones pedunculin (5,8-dihydroxy-6,7,4′-trimethoxyflavone) and 5,8-dihydroxy-6,7,3′,4′-tetramethoxy flavone [62,84]. Jantaharn et al. analyzed the phytochemistry of *H. tuberosus* flowers, leading to the isolation of the chalcone isoliquiritigenin, the flavanone liquiritigenin, the flavones acerosin and quercetin 7-O-glucoside, as well as the aurones sulfuretin and sulfuretin glycoside. Compound identification was achieved through chromatographic separation followed by spectroscopic characterization and comparison with literature data [85].

Isoquercetin was detected in *H. angustifolius* L., *H. carnosus*, *H. floridanus* A. Gray., *H. heterophyllus* Nutt., *H. longifolius* Pursh., and *H. microcephalus* T. & G. [22], and was subsequently identified in *H. tuberosus* by Kaszás et al. [80]. The flavone quercetin 7-*O*-glucoside was reported in *H. floridanus* A. Gray., *H. carnosus*, and *H. microcephalus* T. & G. [22]. The chalcone isoliquiritigenin was initially identified in *H. longifolius* Pursh [22] and was subsequently documented in *H. tuberosus* [84]. The flavone luteolin was first reported in *H. microcephalus* T. & G., and was later documented in *H. annuus*, as summarized by Singh et al. [76]. The flavone acerosin was described in *H. strumosus*, *H. simulans*, and *H. microcephalus* T. & G as well as in *H. annuus* and *H. tuberosus* [76,85]. Sudachitin was found in *H. simulans* and *H. strumosus* L. [82].

A study comparing seeds and sprouts of *H. annuus* reported different phytochemical profiles, including flavonoids and phenolic acids. Flavonoids such as dihydrofavonol, kaempferol, apigenin, quercetin, genistein, genistin, daidzein, daidzin, biochanin A and formononetin were identified, together with phenolic acids such as 5-O-*p*-coumaroylquinic acid, 5-O-feruloylquinic acid, among others [86]. In another study employing high-performance liquid chromatography (HPLC), numerous chlorogenic acids were detected in *H. annuus* seeds extract, including 3-O-Caffeoylquinic acid, 4-O-Caffeoylquinic acid, 3,5-Di-O-caffeoylquinic acid, 4,5-Di-O-caffeoylquinic acid, 3,4-Di-O-caffeoylquinic acid, and 5-O-Caffeoylquinic acid, the last being the predominant compound [87].

The chalcone kukulkanin B was identified in *H. annuus*, along with flavanone, rutin, and the flavanone heliannone B, in addition to other previously reported flavonoids [76]. Phenolic acids such as sinapic acid, gallic acid, *p*-coumaric acid, *cis*-ferulic acid, *trans*-ferulic acid, caffeic acid, and protocatechuic acid have also been reported [76].

The most relevant flavonoid and phenolic compounds isolated from *Helianthus* species are listed in Table 3.

To facilitate comparison across species, Table 4 provides a compiled overview of the occurrence and identification confidence of the most representative flavonoids in *Helianthus* species, based on the evaluated literature. The following confidence codes were applied: L (literature-only), referring to flavonoids reported solely in the literature; MS (MS tentative), corresponding to compounds identified by HPLC–MS/MS; SC (standard confirmed), referring to flavonoids identified by spectroscopic methods and confirmed by comparison with authentic reference compounds; and a hyphen (-), indicating compounds not reported.

### 4.3. Flavonoids of Helianthus: A Chemotaxonomic Perspective

General chemotaxonomic studies on the genus *Helianthus* were primarily conducted during the 1980s. The methodologies employed included thin-layer chromatography (TLC) for Rf characterization and UV spectroscopy; therefore, further studies using modern advanced analytical techniques are necessary to properly validate these results. Briefly, these works cover the series *Corona-solis*, *Angustifolii*, and *Microcephali*. Although this subdivision has been a subject of debate [10], this categorization will be maintained for the purpose of analyzing these publications.

The study by Schilling & Mabry [88] focused on the series *Corona-solis*, analyzing the leaves of the following species: *H. divaricatus*, *H. giganteus*, *H. grosseserratus*, *H. hirsutus, H. maximiliani, H. mollis, H. nuttallii, H. decapetalus, H. strumosus, H. californicus, H. eggertii, H. resinosus, H. schweinitzii*, and *H. tuberosus*. This research found that foliar flavonoids consisted of a mixture of 6-methoxyflavones, flavonol glycosides, and, more rarely, the chalcones sulfuretin 6-glucoside, sulfuretin 6-galactoside, and coreopsin. These latter two classes of compounds are primarily characteristic of the head flowers, where they serve as nectar guides for pollination; thus, their presence in leaves represents a distinctive trait for the species containing them. The predominant flavonol glycoside in the leaves was kaempferol 3-glucoside, present in all studied species, followed by kaempferol 3-rutinoside, quercetin 3-glucoside, and kaempferol 3-galactoside. On the other hand, 6-methoxyapigenin, 6-methoxyluteolin, and 6,4’-dimethoxyapigenin were the most frequent methoxylated flavones in this series. *H. decapetalus* lacked 6-methoxyflavones, while *H. schweinitzii* contained only 6-methoxyapigenin [88].

Consistent with observations of STLs, *H. tuberosus* was among the species with the greatest diversity of compounds. *H. tuberosus*, *H. strumosus*, and *H. maximiliani* contained all three flavonoid classes, while chalcones were absent in the remaining species of this series. As previously mentioned, *H. decapetalus* lacked 6-methoxylated flavones, although it was the only species where quercetin 7-glucoside was detected. The polymethoxylated flavones nevadensin and hymenoxin were identified in *H. schweinitzii*.

Regarding the series *Angustifolii*, the species *H. angustifolius*, *H. carnosus*, *H. floridanus*, *H. heterophyllus*, *H. longifolius*, *H. radula*, and *H. simulans* were studied [89]. The same subclasses of foliar flavonoids as in the *Corona-solis* series were identified, although glycosides occurred in very low concentrations. Furthermore, methoxylated flavones with 6-substitution (hispidulin, jaceosidin, and nepetin) and 6,8,4’-substitution (hymenoxin and nevadensin) patterns were found in *H. angustifolius*, *H. floridanus*, and *H. simulans*. These aglycones were concentrated in glandular trichomes; notably, species lacking aglycones also lacked these trichomes. Regarding chalcones, sulfurein was found in the leaves of *H. heterophyllus*. *H. longifolius* possessed chalcone aglycones, one of which was identified as isoliquiritigenin, while another remained undetermined. It is important to highlight that this species is glabrous; therefore, the authors suggest that these aglycones are located in epidermal structures other than trichomes.

In the *Microcephali* series [90], the analysis of flower flavonoids revealed the chalcones coreopsin and sulfurein, alongside quercetin 7-glucoside. Additionally, *H. microcephalus* contains quercetin 3-glucoside. Acetone rinses of *H. microcephalus* leaves yielded luteolin, acerosin (5,7,3’-trihydroxy-6,8,4’-trimethoxyflavone), nepetin (6-methoxyluteolin), jaceosidin (6-methoxyluteolin 3′-methyl ether), hymenoxin (6,8-dimethoxyluteolin 3′,4-methyl ether), hispidulin (6-methoxyapigenin), and nevadensin (6,8-dimethoxyapigenin 4′-methyl ether). In this context, the occurrence of luteolin and its methoxylated derivatives is noteworthy as a chemotaxonomic marker. Furthermore, a chalcone was detected, but the techniques employed did not allow for its identification. These compounds appeared to be sequestered in glandular trichomes, as the other two species studied, *H. glaucophyllus* and *H. laevigatus*, lacked this class of compounds.

Wild *Helianthus* species have been investigated as genetic reservoirs to enhance disease resistance in *H. annuus* [91,92,93]. Consequently, it is imperative to conduct further chemotaxonomic, genetic, and ecological research. Such studies should focus not only on identifying compounds using advanced techniques like high-resolution mass spectrometry (HRMS) but also on determining their distribution within plant organs and their seasonal variation in both annual and perennial species.

### 4.4. Integrated Phytochemical, Taxonomic, and Geographic Patterns in Helianthus

**Figure 6:**
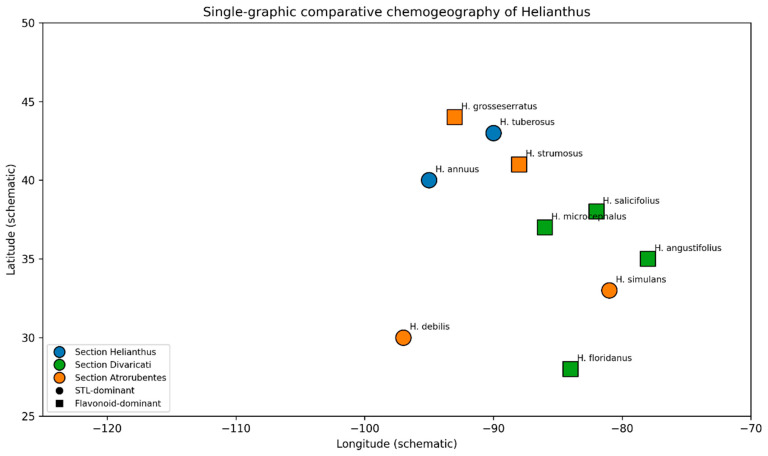
Integrated representation of geographic distribution, taxonomic section, and dominant phytochemical class in *Helianthus* species. Points indicate species sampled across North America; colors denote taxonomic sections (blue, Section *Helianthus*; green, Section *Divaricati*; orange, Section *Atrorubentes*), and symbol shapes indicate dominant chemistry (circles, sesquiterpene lactone–dominant; squares, flavonoid-dominant).

To allow comparative interpretation of phytochemical diversity across the genus *Helianthus*, a single-graphic chemogeographic map was constructed integrating taxonomic, chemical, and geographic information (Figure 6). This visualization summarizes the distribution of major secondary metabolite classes—STLs and flavonoids—across selected *Helianthus* species, grouped by taxonomic section and overlaid with their reported sampling locations.

## 5. Biological Activity of *Helianthus* Extracts

### 5.1. Anti-Diabetic, Antidislipidemic, and Hepatoprotective Effects

The in vitro inhibitory effects of various extracts (ethyl acetate, hexane, and acetone) from *H. annuus* leaves on diabetes-related enzymes α-glucosidase and α-amylase were evaluated by Ojo et al. [94]. The hexane extract demonstrated the highest potency, significantly inhibiting α-glucosidase and α-amylase activities, with IC_50_ values of 3.29 ± 0.12 mg/mL and 3.92 ± 0.02 mg/mL, respectively. Lineweaver-Burk plot analysis indicated a non-competitive inhibition pattern for α-glucosidase and a competitive inhibition pattern for α-amylase with this extract [94]. Although these findings indicate strong enzyme-level antidiabetic potential, they are based exclusively on in vitro assays; therefore, further in vivo studies are necessary to substantiate their antidiabetic efficacy.

According to Al-Snafi’s review published in 2018, various extracts of *H. annuus* (sunflower) have demonstrated significant antidiabetic and antiglycative properties on in vivo rat models, including studies performed in normoglycemic, glucose-loaded hyperglycemic, and streptozotocin (STZ)-induced type 2 diabetic rats. Oral administration of the ethanolic seed extract (HSE; 250 and 500 mg/kg) produced a marked reduction in blood glucose levels (*p* < 0.001) in streptozotocin-nicotinamide induced diabetic rats, comparable to the standard drug glibenclamide (600 μg/kg). Additionally, HSE improved several metabolic parameters, including body weight, hepatic glycogen, lipid profile, glycosylated hemoglobin, glutathione, serum insulin, and plasma malondialdehyde levels. The extract also reduced blood glucose level in normoglycemic rats (*p* < 0.05), whereas a much greater reduction in blood glucose levels was observed in diabetic rats (*p* < 0.01) [75].

A crude methanolic extract of *H. annuus* was fractionated and evaluated in vivo for antidiabetic activity in an alloxan-induced hyperglycemic rat model. Among the thirteen fractions obtained, fractions 8, 9, 10, and 13 (60 mg/kg) produced significant time-dependent reductions in fasting blood glucose levels. Their antihyperglycemic effects were compared with those of the crude extract administered at 600 mg/kg. At 6 h post-administration, the crude extract, glibenclamide, and fractions 8, 9, 10, and 13 reduced fasting blood glucose levels by 66.74%, 57.43%, 61.36%, 59.80%, 70.63%, and 78.03%, respectively [75].

Additionally, *H. annuus* methanolic leaf extract was also evaluated for antidiabetic, oral glucose tolerance, and antioxidant activities in alloxan-induced diabetic rats. Oral administration of the extract at 150, 300, and 600 mg/kg produced a significant, dose- and time-dependent reduction in blood glucose levels (*p* < 0.05). At 6 h post-treatment, the highest dose (600 mg/kg) reduced blood glucose by 66.74% compared with the negative control. In normoglycemic rats, the oral glucose tolerance test (OGTT) showed no significant differences among treatment groups (*p* > 0.05), whereas in diabetic rats the extract (600 mg/kg) significantly lowered blood glucose levels at 120 min post-glucose load compared with the negative control (*p* < 0.05), with no significant difference relative to glibenclamide (2 mg/kg). The extract also exhibited a concentration-dependent increase in antioxidant activity [75].

Similarly, the antiglycative and antioxidant activities of *H. annuus* sprouts were evaluated using in vitro formation of advanced glycation end products (AGE-formation) and antioxidant assays. At 1.0 mg/mL inhibited AGE-formation by 83.29%, surpassing the reference compound aminoguanidine (80.88% at 1 mM) [75]. Although these results indicate strong in vitro antiglycative and antioxidant potential, in vivo validation is still lacking.

The hepatoprotective activity of aqueous and ethanolic extracts from *H. annuus* flowers was evaluated in an in vivo CCl4-induced hepatotoxicity model in Wistar rats. Treatment with these extracts significantly (*p* < 0.001) reduced elevated levels of serum enzymes such as alkaline phosphatase, total bilirubin, glutamate pyruvate transaminase, and glutamate oxaloacetate transaminase in rats exposed to CCl4 and treated with 200 mg/kg body weight. The biochemical effects of the extracts were further confirmed through histopathological examinations of the liver [75].

Furthermore, the hydromethanolic leaf extract of *H. annuus* was evaluated in vivo in an alloxan-induced diabetic rat model for its hypoglycemic, antidislipidemic, and hepatoprotective properties. The extract was administered orally at 150, 300, and 600 mg/kg once daily for 21 consecutive days, with glibenclamide (2 mg/kg) used as a positive control and 5% Tween-20 as a negative control. Significant (*p* < 0.05) improvements were observed in fasting blood glucose levels, malondialdehyde levels, lipid profile, and glycosylated hemoglobin in rats treated with *H. annuus* extract and glibenclamide, accompanied by improved body weight gain, compared to the negative control group. Moreover, evidence of reversal of alloxan-induced hepatic and pancreatic degeneration was noted in rats treated with both glibenclamide and the extract, supporting the in vivo antidiabetic, antidislipidemic, and hepatoprotective potential of *H. annuus* leaf extracts [95].

A study by Brobbey et al. evaluated the in vivo hepatoprotective activity of a methanolic extract from *H. annuus* seeds using a paracetamol-induced hepatotoxicity model in rats. Following administration of the extract at various doses (100, 300, and 500 mg/kg once daily) over 7 days and subsequent induction of hepatotoxicity with acetaminophen, a significant reduction in hepatic enzyme levels such as alkaline phosphatase, aspartate aminotransferase, and alanine aminotransferase was observed (*p* < 0.0001), indicating protection against liver injury. These findings suggest that the extract exhibits potential hepatoprotective properties, possibly due to its antioxidant capacity and high phenolic content (40.60 ± 1.14 mg gallic acid equivalents/g) and measurable flavonoid levels (7.72 ± 2.3 mg quercetin equivalents/g) [96].

The methanolic extract and the n-hexane, methanolic and ethyl acetate fractions of *H. tuberosus* tubers were evaluated for their antidiabetic activity using in vitro enzyme inhibition assays against α-amylase and α-glucosidase. Among these, the ethyl acetate fraction demonstrated the strongest inhibitory activity, with IC_50_ values of 187.04 ± 0.42 μg/mL for α-glucosidase and 102.53 ± 1.39 μg/mL for α-amylase. To further explore the metabolic relevance of these findings, glucose uptake was assessed in an insulin-resistant HepG2 cell line, demonstrating that the ethyl acetate fraction may enhance glucose uptake in these insulin-resistant cells [97]. Collectively, the evidence is summarized in Table S2. Data indicates that the antidiabetic and hepatoprotective activities reported for *Helianthus* species—predominantly *H. annuus*, and minor representation of *H. tuberosus*—are mainly supported by in vivo animal models (Level A), complemented by in vitro enzymatic and cellular assays (Levels C, D). To date, no clinical studies have been reported for either species. Importantly, most of the available data are based on crude or partially characterized extracts, which limit reproducibility and preclude clear attribution of the observed effects to specific bioactive constituents. Consequently, further studies focusing on compound isolation, mechanism of action, pharmacokinetics, and safety are required.

### 5.2. Analgesic and Anti-Inflammatory Activities

The methanolic seed extract of *H. annuus* demonstrated significant analgesic activity in vivo using rodent pain models. In the acetic acid-induced writhing test, the extract markedly inhibited pain responses, achieving 50.35% and 57.85% inhibition at doses of 100 and 200 mg/kg, respectively (*p* < 0.05). Likewise, in the hot plate test, it significantly prolonged reaction latency compared with aspirin, with response times of 13 ± 0.91 s and 16.5 ± 1.55 s at 60 min for the 100 and 200 mg/kg doses, respectively. The ethanolic leaf extract also demonstrated potent analgesic and anti-inflammatory effects in vivo at doses of 0.5–4 g/kg. In tail immersion and hot plate tests, the extract markedly increased pain tolerance, showing greater efficacy than indomethacin (10 mg/kg). In the albumin-induced paw edema model, it significantly reduced inflammation, again surpassing the anti-inflammatory effect of indomethacin [75,76]. Consistently, Onoja et al. reported that the methanolic leaf extract (HAE) of *H. annuus* (150–600 mg/kg) produced dose-dependent analgesic and anti-inflammatory effects in vivo. In edema models, HAE at 300 mg/kg significantly reduced paw volume within three hours. In the writhing test, HAE (600 mg/kg) and acetylsalicylic acid similarly reduced pain episodes, while in the hot plate test, HAE (300 mg/kg) and pentazocine significantly increased pain reaction time [98]. These studies provide consistent in vivo evidence supporting the analgesic and anti-inflammatory potential of *H. annuus*; however, mechanistic studies and compound-level attribution remain limited.

### 5.3. Anti-Ulcer Activity

The anti-ulcer activity of hydroalcoholic leaf extracts of *H. annuus*, *Abutilon indicum*, and their combination was evaluated in vivo using pyloric ligation-induced and ethanol-induced gastric ulcer models in Albino Wistar rats. The extracts were administered orally at 200 and 400 mg/kg, following acute toxicity evaluation, with omeprazole (20 mg/kg) used as the reference drug. In the ethanol-induced ulcer model, *H. annuus* extract produced a dose-dependent gastroprotective effect, achieving 57.79% and 62.41% inhibition of gastric lesions at 200 and 400 mg/kg, respectively, compared with 81.67% inhibition for omeprazole. In the pyloric ligation model, the same extract showed 61.69% and 67.18% inhibition at the corresponding doses, versus 78.90% for the standard drug. Notably, the combined extract of *A. indicum* and *H. annuus* exhibited the strongest anti-ulcer activity in both models, reaching 75.23% inhibition (ethanol-induced ulcers) and 74.21% inhibition (pyloric ligation) at 400 mg/kg, approaching the efficacy of omeprazole. Overall, the protective effects followed the order combined extract > *H. annuus* > *A. indicum*, supporting the in vivo gastroprotective potential of *H. annuus*, particularly in combination with *A. indicum* [99].

### 5.4. Antihistaminic Activity

The ethanolic extract of *H. annuus* leaves demonstrated significant antihistaminic activity in both a microshock model in rabbits and in guinea pigs through histamine-induced bronchoconstriction [76]. Antihistaminic activity was evaluated in vivo using histamine-induced bronchoconstriction models in guinea pigs (exposed to 0.1% of histamine aerosol) and rabbits (exposed to 0.2% of histamine aerosol). The extract significantly prolonged the preconvulsive dyspnea time, providing 46.54 and 62.15% protection in guinea pigs and 52.55 and 70.69% protection in rabbits after four hours at 250 and 500 mg/kg (*p* < 0.001) [76].

### 5.5. Antidiarrheal Effects

The ethanolic extract of *H. annuus* leaves exhibited antidiarrheal activity in vivo using castor oil-induced diarrhea and gastrointestinal transit models in mice [76]. Oral administration of the extract significantly reduced fecal output by 42.15% and 67.01% at doses of 250 and 500 mg/kg, respectively (*p* < 0.001), and decreased intestinal transit by 27.59% and 48.62% compared with the control group [76].

### 5.6. Anti-Fertility Effect

The anti-fertility effects of the ethanolic extract of *H. annuus* leaves on Wistar rats have been investigated in vivo. Evaluation of epididymal sperm properties, blood levels of reproductive hormones, and testicular histology revealed fertility-reducing effects of the extract. In another study, the impact of the ethanolic extract of *H. annuus* leaves on rat fecundity was assessed. Administered orally at a dose of 0.5 g/kg for 2 weeks, the extract significantly decreased both the number of pups per rat and the pregnancy rate, although the frequency of copulation remained unaffected. The histo-degenerative alterations observed in the gonads may account for the reduced reproductive performance in adult rats treated with *H. annuus* leaf extract [75,76].

### 5.7. Anti-Hyperuricemic and Anti-Gouty Arthritis Activity

The therapeutic potential and mechanisms of sunflower head extract (SHE) from *H. annuus* in gout and hyperuricemia models were investigated in vivo by Lanzhou Li et al. [100]. Among the tested preparations, SHEB (20% ethanol:80% water extract) showed the most pronounced activity. In a monosodium urate (MSU)–induced acute gout model in Sprague–Dawley rats, oral administration of SHEB at 1 g/kg/day for 8 days significantly reduced ankle swelling by 16.2% at 12 h and 27.1% at 48 h compared with the untreated gout model (*p* < 0.05). Additionally, SHEB markedly alleviated joint damage by reducing inflammatory cell infiltration and restoring joint space.

In the acute gout model, SHEB (1 g/kg) increased serum levels of the anti-inflammatory cytokine interleukin-10 (IL-10) (35.5 ± 4.9 pg/mL vs. 22.3 ± 3.0 pg/mL in the model group, *p* < 0.001), and monocyte chemoattractant protein-1 (MCP-1), and elevated macrophage inflammatory protein-1α (MIP-1α), suggesting modulation of inflammatory responses [100].

In parallel, the antihyperuricemic effects of SHEB were evaluated in vivo in an induced hyperuricemia model in BALB/c mice. Oral administration of SHEB (1 g/kg/day for 8 days) significantly reduced serum uric acid levels by approximately 50% compared with hyperuricemic controls (*p* < 0.05), an effect comparable to allopurinol (20 mg/kg). Moreover, SHEB significantly inhibited xanthine oxidase (XO) activity, decreasing XO levels by 13.1% in serum and 40.2% in liver tissue (*p* < 0.05–0.01). The extract also attenuated oxidative stress, particularly in liver tissue, by markedly reducing malondialdehyde (66.5%), nitric oxide (59.1%), superoxide dismutase (59.2%), and glutathione peroxidase (65.2%) levels (*p* < 0.001) [100]. These findings demonstrate that SHEB exerts significant in vivo anti-gout and antihyperuricemic effects, likely mediated through suppression of XO activity, modulation of oxidative stress, and enhancement of anti-inflammatory cytokine production.

### 5.8. Anti-Obesity Effect

The anti-obesity effects of the methanolic extract of *H. annuus* seeds (HMS) were investigated using an in vivo mouse model. Over a period of 6 weeks, mice were treated daily with atorvastatin (10 mg/kg), a cafeteria diet, and HMS (200 mg/kg). Parameters evaluated included body weight, body mass index (BMI), food consumption, Lee index of obesity (LIO), locomotor activity, glucose, triglycerides total cholesterol, HDL, and LDL levels. HMS significantly reduced body weight, food consumption, LIO, BMI, glucose, triglycerides, total cholesterol, and LDL levels, while significantly increasing locomotor activity and HDL levels [75,76].

Two polyherbal formulations containing *H. tuberosus* root powder demonstrated significant anti-obesity effects. On a diet-induced obesity model in mice, both formulations effectively reduced body weight compared to a high-fat diet control group. Additionally, these formulations improved lipoprotein imbalances caused by obesity and reduced the atherogenic index. Notable reductions in liver and epididymal white adipose tissue weight further suggest their potential as adjunct treatments for obesity [101].

### 5.9. Renoprotective Effect

The effects of ethanolic and aqueous extracts of *H. annuus* leaves on calcium oxalate nephrolithiasis model in male rats (hyperoxaluria induced by ammonium chloride and ethylene glycol) were evaluated. Each extract was administered at a dose of 500 mg daily for 10 days. Induction of hyperoxaluria and increased renal excretion of phosphorus and calcium was achieved through feeding with ammonium chloride and ethylene glycol. Both ethanolic and aqueous extracts significantly reduced the elevated deposition of stone-forming constituents in the kidneys of calculogenic rats [75].

Table S3 consolidates the available data for *Helianthus* species using standardized descriptors of assay type, model system, outcome measures, and evidence level. As summarized in Table S3, the reported biological activities of *Helianthus* species—predominantly *H. annuus*—are supported mainly by preclinical evidence, including in vivo animal models. The frequent use of crude extracts represents a major limitation, as it precludes precise identification of active constituents and mechanisms of action. Notably, the lack of clinical studies emphasizes that these findings should be interpreted cautiously and regarded as preliminary.

### 5.10. Antibacterial and Antifungal Activity

The antibacterial and antifungal activities of frozen, freeze-dried, and dried ethanolic extracts from *H. annuus* bee-pollen extracts were investigated in vitro using the agar well diffusion method by Fatrcová-Šramková et al. [102]. Antimicrobial activity is expressed as inhibition zone diameter (mm, mean ± SD). The frozen extracts were effective against *Pseudomona aeruginosa* (2.27 ± 0.15), *Paenibacillus larvae* (2.38 ± 0.38), and *Escherichia coli* (2.70 ± 0.56), and demonstrated additional antifungal activity against *Aspergillus ochraceus* (1.77 ± 0.25). Freeze-dried extracts were active against *Enterococcus raffinosus* (2.57 ± 0.21), *Brochotrix thermosphacta* (2.64 ± 0.15), and *P. larvae* (2.73 ± 0.21), with further antifungal effects against *Aspergillus niger* (1.77 ± 0.15). Dried extracts exhibited antibacterial activity against *P. aeruginosa* (2.23 ± 0.15), *E. raffinosus* (2.00 ± 0.10), and *P. larvae* (2.60 ± 0.20) [102].

The antimicrobial effects of aqueous and ethanolic extracts from *H. annuus* flowers were evaluated in vitro against multidrug-resistant (MDR) bacterial strains isolated from clinical samples using disc diffusion and tube dilution assays. The ethanolic extract inhibited MDR *Pseudomonas* and *E. coli* with minimum inhibitory concentrations (MICs) of 2.5 mg/mL and 4 mg/mL, respectively. In contrast, the aqueous extract showed no detectable activity against MDR *Pseudomonas*, *Klebsiella*, or *E. coli* but exhibited transient antibacterial effects against MDR *Staphylococcus* strain [103]. These results indicate that the antibacterial efficacy of *H. annuus* flowers against MDR pathogens is strongly dependent on the extraction solvent, with ethanolic extracts showing superior activity in vitro, highlighting their potential as a source of antimicrobial agents for combating resistant bacterial infections [103].

The methanolic extract from *H. annuus* seeds was evaluated in vitro for its antibacterial and antifungal activities against a range of microorganisms, including *Vibrio cholerae*, *Salmonella typhi*, *Fusarium oxysporum*, *Bacillus subtilis*, *Staphylococcus aureus*, *Candida albicans*, *Rhizopus stolonifer*, and *Aspergillus fumigatus*. Antibacterial activity was assessed using an agar diffusion assay, while antifungal effects were determined through antifungal susceptibility testing and agar well diffusion methods. At a concentration of 50 µg/mL, the seed extract exhibited high antibacterial activity against *S. typhi*, producing an inhibition zone of 1.5 cm, moderate activity against *S. aureus* (1.2 cm) and *V. cholerae* (1.1 cm), and low activity against *B. subtilis* (0.7 cm). These antibacterial effects were reported to be comparable to those of the reference antibiotic ampicillin (10 µg/mL). In the antifungal assays, the methanolic seed extract (50 µg/mL) showed strong inhibitory activity against *A. fumigatus* (1.3 cm) and *R. stolonifer* (1.2 cm), moderate activity against *C. albicans* (1.0 cm), and weak activity against *F. oxysporum* (0.5 cm), which was considered resistant to the extract. In summary, these findings confirm the broad-spectrum in vitro antimicrobial potential of *H. annuus* seed methanolic extracts, with differential sensitivity among bacterial and fungal species [75].

The antibacterial effects of aqueous and ethanolic leaf extracts of *H. annuus* were evaluated in vitro using disc diffusion and agar well diffusion assays. The aqueous extract showed weak to moderate inhibitory activity against *S. aureus*, *Klebsiella pneumoniae*, *P. aeruginosa*, *B. subtilis*, *E. coli*, *Salmonella typhimurium*, and *Micrococcus luteus*, with inhibition zones generally ranging from 1.1 to 2.1 mm depending on the assay. In contrast, the ethanolic extract demonstrated significant antibacterial activity against these bacteria in both evaluation methods, producing inhibition zones of approximately 5.2–7.1 mm across both diffusion methods, indicating a substantially higher antibacterial potency compared with the aqueous extract [75].

The antimicrobial properties of an ethanolic stem extract of *H. annuus* (HMT) were also tested in vitro against *E. coli*, *S. aureus*, *C. albicans*, and *Aspergillus niger*. HMT exhibited antimicrobial activity against *A. niger*, *C. albicans*, and *S. aureus*, but *E. coli* showed resistance. The Minimum Bactericidal/Fungicidal Concentrations (MBC/MFC) and MIC values were 80 and 80 mg/mL for *A. niger*, 70 and 50 mg/mL for *C. albicans*, and 90 and 70 mg/mL for *S. aureus*, respectively [75].

Additionally, the antimicrobial effects of a seed oil extract from *H. annuus* were investigated for its potential in treating diaper dermatitis. The extract inhibited the growth of various microorganisms, including *P. aeruginosa*, *Staphylococcus epidermidis*, *S. aureus*, *C. albicans*, *Proteus vulgaris*, and *E. coli* at different concentrations and was effective as a topical treatment for diaper dermatitis [75]. Supporting these findings, in vivo clinical evidence demonstrated that topical application of sunflower seed oil three times daily in preterm infants (<34 weeks gestation) significantly improved skin condition (*p* = 0.037) and reduced the incidence of nosocomial infections (adjusted incidence ratio 0.46; 95% CI: 0.26–0.81; *p* = 0.007), with no reported adverse effects [75].

In another study, Akpor et al. explored the in vitro antibacterial potential of *H. annuus* leaf extracts obtained using n-hexane, methanol, and ethyl acetate. Antibacterial activity was evaluated by the agar diffusion method against *P. aeruginosa*, *S. aureus*, *K. pneumoniae*, *B. subtilis*, and *E. coli*. The n-hexane and ethyl acetate extracts selectively inhibited *P. aeruginosa*, *E. coli*, and *B. subtilis*, while the methanol extract inhibited the growth of all the evaluated bacterial species [104]. The inhibitory activity was concentration dependent, with MIC values ranging from approximately 1000 to 3000 mg/L depending on the extract and bacterial species [104]. However, these findings are limited to in vitro assays, and further in vivo studies are required to confirm the therapeutic relevance of these antibacterial effects.

Extracts from the aerial parts of *H. salicifolius* and *H. tuberosus*, obtained by carbon dioxide supercritical fluid extraction with water as a co-solvent, were evaluated in vitro for their antimicrobial and antioxidant activities. Both extracts exhibited antimicrobial effects, with the most pronounced activity against *S. aureus* ATCC 29213, with *H. salicifolius* extract exhibiting a MIC of 0.62 mg/mL and *H. tuberosus* showing a MIC of 2.5 mg/mL, as verified by time–kill assays. *H. tuberosus* extract also demonstrated greater antioxidant activity (EC_50_ = 0.332 mg/mL) compared to *H. salicifolius* (EC_50_ = 0.609 mg/mL). Consistently, the total polyphenol content (TPC) was higher in *H. tuberosus* (33.06 ± 0.80 mg gallic acid equivalents (GAE)/g) than in *H. salicifolius* (13.75 ± 0.50 mg GAE/g). A positive correlation was observed between antioxidant activity and TPC, whereas no clear correlation was found between antistaphylococcal activity and total polyphenol content [105].

In a study conducted by Alexandrino et al., the antimicrobial activity of phenolic-rich crude extracts from *H. annuus* seeds was evaluated in vitro. Phenolic-rich crude extracts were obtained using ethanol and sodium bisulfite as extraction solvents, and their antimicrobial activity was tested against *S. aureus*, *E. coli*, *B. subtilis*, and *P. aeruginosa*. Among the tested preparations, the ethanolic extract demonstrated significant antimicrobial activity against all tested bacteria, with *E. coli* being particularly susceptible, exhibiting a MIC value of 11.6 mg chlorogenic acid equivalents (CGA)/mL, followed by *B. subtilis* (26.5 mg CGA/mL), *S. aureus* (33.2 mg CGA/mL), and *P. aeruginosa* (33.2 mg CGA/mL) [106].

Finally, the antimicrobial activity of an ethanolic leaf extract of *H. annuus* and its lycopene-enriched variant was evaluated in vitro against multidrug-resistant *Streptococcus agalactiae* and *Streptococcus pyogenes*. Antibacterial activity was assessed using disc diffusion assays and confirmed by MIC determination with the Alamar blue method. The ethanolic leaf extract alone inhibited *S. agalactiae* and *S. pyogenes* at concentrations of 175 µg/mL and 125 µg/mL, respectively, comparable to standard antibiotics. Notably, supplementation with lycopene (1 µM) significantly enhanced antibacterial efficacy, reducing the MIC to 80 µg/mL for *S. agalactiae* and 50 µg/mL for *S. pyogenes* [107].

### 5.11. Antiparasitic Activity

The leishmanicidal activity of leaf extracts from *H. annuus* was evaluated in vitro by Mohamed et al. [108] against *Leishmania donovani* promastigotes. The chloroform and petroleum ether extracts demonstrated significant antileishmanial activity, with IC_50_ values of 3.0 and 4.5 μg/mL, respectively, indicating strong activity against the promastigote stage of the parasite.

*Helianthus annuus* extracts were evaluated for antiplasmodial activity. In vitro assays against *Plasmodium falciparum* K1 strain showed that seed extracts exhibited notable antiplasmodial potency. The petroleum ether extract of *H. annuus* showed an IC_50_ of 0.6 μg/mL, while the methanol extract exhibited a lower IC_50_ of 0.1 μg/mL, indicating significant antiplasmodial activity ranging from moderate to strong. In addition, the antiplasmodial potential of ethanolic leaf extracts was assessed in vivo using *P. berghei*-infected Swiss albino mice. Administration of doses at 2 g/kg and 4 g/kg body weight per day for three days resulted in chemo suppression rates of 98.1% and 98.3%, respectively [75].

Furthermore, ethanolic extracts from various parts of *H. annuus* (seeds, flowers, roots, leaves, and stems) were also evaluated for their antimalarial properties by Ekasari et al. [109]. In vitro assays against *P. falciparum* 3D7 strain revealed that the root extract exhibited the highest antiplasmodial activity, with an IC_50_ value of 2.3 ± 1.4 μg/mL, followed by the leaf extract (IC_50_ = 4.3 ± 2.2 μg/mL). In vivo assays using *P. berghei*-infected BALB/c mice demonstrated that the ethanolic root extract at 100 mg/kg resulted in 63.6 ± 8.0% parasite inhibition in the 4-day suppressive test. Further in vivo studies showed that the root extract exhibited significant prophylactic activity, achieving 79.2% inhibition at a dose of 400 mg/kg and strong curative efficacy, with an ED_50_ value of 10.6 ± 0.2 mg/kg. Additionally, in the heme detoxification inhibition assay, both leaf and root extracts exhibited lower IC_50_ values (0.5 and 0.4 mg/mL, respectively) compared to the positive control (chloroquine), indicating that inhibition of heme detoxification may contribute to their antimalarial mechanism of action [109].

Table S4 consolidates the antimicrobial and antiparasitic activities reported for *Helianthus* species, classified according to experimental model, potency metrics, and strength of evidence.

Overall, the antimicrobial and antiparasitic activities summarized in Table S4 are supported predominantly by in vitro microbiological screening assays (Levels C–D), including agar diffusion, MIC determination, and protozoal growth inhibition tests, with comparatively few in vivo animal studies. While several *Helianthus* extracts—particularly those from *H. annuus*—exhibited measurable antibacterial, antifungal, antiprotozoal, and antiplasmodial effects, most studies relied on crude extracts. Importantly, antimicrobial potency was frequently expressed as inhibition zone diameters or MIC values in the mg/mL range. Except for sunflower seed oil evaluated clinically for topical use in neonates, no human data are available. Consequently, although these findings highlight the broad antimicrobial potential of *Helianthus* species, they should be interpreted as preliminary. Further studies are required before any therapeutic or clinical relevance can be inferred.

### 5.12. Antioxidant Activity

Multiple studies have demonstrated strong antioxidant activity in extracts derived from various parts of *H. annuus*, assessed through diverse in vitro assays.

- Cotyledons and Pollen Extracts

Giada and Mancini-Filho evaluated the in vitro antioxidant capacity of aqueous and ethanolic extracts from *H. annuus* cotyledons obtained by sequential extraction with solvents of varying polarities. Antioxidant activity was determined through DPPH radical scavenging assay, oxygen radical absorbance capacity (ORAC), and ferric reducing antioxidant power (FRAP) assays. In all assays, the aqueous extract tested at 30 µg/mL exhibited significantly higher antioxidant potential in all tests, with inhibition values of 50.18% (DPPH), 1.5 Trolox equivalents (ORAC), and 45.27 µmol (FRAP), compared to the ethanolic extract. By contrast, the ethanolic extract showed lower antioxidant capacity across all methods (DPPH 15.21%, ORAC 0.50 Trolox equivalents, FRAP 32.17 µmol). Depending on the method employed, the aqueous extract achieved 45–66% of the activity observed for the synthetic antioxidant butylated hydroxytoluene (BHT), highlighting the strong antioxidant potential of sunflower seed cotyledons [110].

Fatrcová-Šramková et al. also evaluated the antioxidant activity in vitro of ethanolic extracts of *H. annuus* bee pollen obtained through different preservation methods—frozen, freeze-dried, and dried. All extracts displayed moderate radical scavenging capacity, with DPPH inhibition values around 48–50%, along with measurable reducing power as evidenced by phosphomolybdic complex formation. Among the tested samples, the freeze-dried extract exhibited the highest antioxidant activity, followed by the dried and frozen extracts [102]. This enhanced activity correlated with a higher total polyphenol content, indicating that polyphenols contributed more substantially to the antioxidant effects than carotenoids. Overall, the results classify sunflower bee pollen extracts as possessing a medium but reproducible antioxidant capacity, strongly influenced by the preservation method employed [102].

- Leaf and Seed Extracts

Mirghani et al. reported that methanolic extracts of *H. annuus* leaves showed moderate radical scavenging activity (55 ± 0.05% at 500 μg/mL) in vitro in the DPPH assay. Fractionation by column chromatography yielded eight fractions, of which fraction seven exhibited the highest antioxidant activity, reducing DPPH radicals by 65% (IC_50_ = 329 μg/mL). This activity was higher than that of the crude methanolic extract. Thin-layer chromatography confirmed the purity of F7, and qualitative assays (cyanidin reaction and AlCl_3_ colorimetric test) suggested that the compound corresponded to a flavonoid of the flavone type [111].

Similarly, Al-Snafi described the antioxidant potential of a crude methanolic *H. annuus* leaf extract and its fractions. The methanolic extract yielded thirteen fractions, which were evaluated in vitro at a concentration of 400 μg/mL using DPPH and FRAP assays. The crude extract, as well as fractions 8, 9, 10, and especially 13, showed notable DPPH radical scavenging activity (89.00%, 30.42%, 47.90%, 88.03%, and 92.72%, respectively). Consistent results were obtained in the FRAP assay with values of 3.69, 0.95, 0.23, 0.67 and 0.28 μM antioxidant potential, respectively. Methanolic seed extracts also demonstrated strong and concentration-dependent DPPH scavenging activity, further supporting the relevance of *H. annuus* phenolic constituents as effective natural antioxidants [75].

Studies on *H. annuus* sprouts available in Chinese markets further confirmed the antioxidant potential of this species. The 80% methanolic extract showed excellent activity with EC_50_ values of 35.00 ± 3.82 μg/mL (DPPH), 18.00 ± 0.31 μg/mL (Fe^3+^ reduction), and 2.93 ± 0.23 μg/mL (β-carotene oxidation protection) [75].

Akpor et al. compared leaf extracts obtained with n-hexane, methanol, and ethyl acetate, observing that the ethyl acetate extract exhibited the highest reducing power and radical neutralizing ability, indicating that medium-polarity solvents may better extract antioxidant constituents [104]. Later, Muhtadi corroborated these findings by evaluating the antioxidant activity of ethanolic extracts obtained from different parts of *H. annuus* (leaves, flowers, and stem bark) using the DPPH radical scavenging assay. Among the tested samples, the leaf extract exhibited the strongest antioxidant activity (IC_50_ = 48.84 ppm), whereas the flower extract showed weaker activity (IC_50_ = 180.50 ppm) and the bark extract showed minimal activity (IC_50_ = 274.03 ppm). This correlated with the high phenolic (35.15 mg gallic acid equivalents/g extract) and flavonoids (10.92 mg quercetin equivalents/g extract) contents of the leaves [112]. A strong positive correlation was observed between DPPH scavenging activity and both phenolic and flavonoid contents, indicating that these compounds are major contributors to the antioxidant potential of *H. annuus* leaf extracts.

In a complementary in vitro study, Alexandrino et al. evaluated phenolic-rich extracts from *H. annuus* seeds and found that ethanolic extracts exhibited greater DPPH scavenging capacity (EC_50_ = 0.36 g extract/g DPPH) compared with sodium bisulfite extracts (EC_50_ = 1.01 g extract/g DPPH), confirming the major efficiency of ethanol for extracting antioxidant phenolic compounds [106]. Moreover, Alshahrani et al. demonstrated that enrichment of ethanolic *H. annuus* leaf extracts with lycopene significantly enhanced their antioxidant properties, reducing reactive oxygen species (ROS) by 24% and increasing DPPH activity by 30%, highlighting the synergistic potential of combining *H. annuus* extracts with other natural antioxidants [107].

*H. tuberosus* has also shown remarkable antioxidant potential in vitro. Rolnik et al. [113] investigated the protective effects of an aqueous- methanolic root extract on human plasma subjected to oxidative stress induced by H_2_O_2_/Fe^2+^ in vitro. The extract inhibited lipid peroxidation, reduced protein carbonylation, and modulated thiol group levels at 50 μg/mL, confirming its capacity to protect biomolecules from oxidative damage. At the highest tested concentration (50 μg/mL), the extract reduced plasma protein carbonylation by approximately 50% compared with plasma exposed to H_2_O_2_/Fe^2+^ alone. In addition, the extract inhibited the oxidation of plasma protein thiol groups. The extract also significantly modulated lipid peroxidation, as evidenced by changes in thiobarbituric acid–reactive substances (TBARS) levels in oxidatively stressed plasma. In contrast, no significant effects were observed on total antioxidant capacity (TAC) or oxygen radical absorbance capacity (ORAC) at 50 μg/mL. Direct radical scavenging activity assessed by TLC–DPPH• analysis revealed moderate antioxidant capacity (0.140 ± 0.01, relative to chlorogenic acid). In conclusion, these results indicate that *H. tuberosus* root extracts exert protective antioxidant effects in plasma primarily through inhibition of protein and lipid oxidation rather than by increasing overall antioxidant capacity.

Mariadoss et al. further evaluated methanolic extracts and their n-hexane and ethyl acetate fractions from *H. tuberosus* tubers. The ethyl acetate fraction displayed the highest free radical scavenging activity in both DPPH and ABTS assays (IC_50_ = 161.55 ± 0.98 and 104.45 ± 3.01 μg/mL, respectively) [97].

In addition, polyherbal formulations containing *H. tuberosus* root powder demonstrated significant antioxidant capacity in both DPPH and ABTS assays, reflecting synergistic interactions among plant components [101].

To provide a structured and critical overview of the antioxidant activity reported for *Helianthus* species, the main findings are summarized in Table S5. The antioxidant activity reported for *Helianthus* species is supported mainly by in vitro chemical assays (Level D), with limited evidence from cellular or ex vivo models (Level C). Most studies are based on crude extracts or fractions, and antioxidant potency is expressed using IC_50_, EC_50_, or percentage inhibition values, which are suitable for preliminary screening but do not predict biological efficacy in vivo. Although antioxidant effects often correlate with total phenolic or flavonoid content, these associations remain descriptive. Notably, no in vivo antioxidant efficacy or clinical studies are available.

### 5.13. Anti-Asthmatic Effect

The effects of an aqueous extract from *H. annuus* seeds (HAS) were investigated in vivo using an ovalbumin (OVA)-induced murine model of asthma. Mice sensitized intraperitoneally with OVA and subsequently challenged with aerosolized OVA, were orally administered the extract, and subsequent lung changes were evaluated. Histopathological evaluation revealed marked inflammatory cell infiltration and leukocyte accumulation in OVA-challenged mice, whereas HAS treatment significantly attenuated these pathological changes, reducing total leukocyte infiltration in lung tissues. Immunohistochemical analysis further demonstrated a reduction in CD4^+^ T-cell infiltration in HAS-treated mice compared with OVA-challenged controls. At the molecular level, ELISA analysis showed that OVA challenge increased IL-4 levels from 106.3 ± 8.9 pg/mL in control mice to 250.2 ± 35.5 pg/mL; however, HAS treatment suppressed IL-4 expression to approximately 42% of OVA-induced levels. Similarly, IL-13 expression was markedly reduced to about 30% in the HAS-treated group, a finding corroborated by immunohistochemical staining of lung tissues. In agreement with these results, serum IgE levels were also significantly decreased following HAS administration, indicating attenuation of the Th2-mediated allergic response. Furthermore, it was observed that within a dosage range of 6 g to 60 g/kg/day, HAS did not induce morphological toxicity at the tissue level [114].

### 5.14. Effect on Atopic Dermatitis

Topical application of *H. tuberosus* (30% ethanol extract from the tubers) alleviated atopic dermatitis (AD) symptoms in vivo in an NC/Nga mouse model of AD induced by *Dermatophagoides farinae* body (Dfb), resulting in a notable reduction in the dermatitis score and production of inflammatory mediators. Daily topical treatment with *H. tuberosus* (100 mg/kg) for 4 weeks markedly reduced the dermatitis score compared with the Dfb-induced group (*p* < 0.001), showing an effect comparable to or greater than dexamethasone. *H. tuberosus* also decreased epidermal thickness and infiltration of mast cells, restored filaggrin expression, and inhibited adhesion molecules, including ICAM-1, VCAM-1, and E-selectin in the mice. These effects were also corroborated in vitro using TNF-α/IFN-γ-stimulated HaCaT keratinocytes. Furthermore, *H. tuberosus* (100–400 μg/mL) suppressed activation of NF-κB, Akt, and mitogen-activated protein kinase signaling pathways induced by TNF-α/IFN-γ [115].

### 5.15. Effect on the Central Nervous System

The methanolic extract of *H. annuus* seeds was evaluated for its effect on the central nervous system (CNS) activity in vivo in mice at doses of 100 and 200 mg/kg. CNS activity was assessed through general behavioral profiling, anxiolytic models (light–dark box and elevated plus maze), and an antidepressant model (tail suspension test). The extract produced significant CNS-stimulating effects, particularly at 200 mg/kg, including increased spontaneous activity, enhanced grip strength, heightened pain response, and intensified motor reflexes such as pinna reflex and tactile reactivity. Moderate improvements in alertness and awareness were also noted. In the light–dark box test, the extract increased the latency to enter the dark compartment from 34 ± 5.63 s in control mice to 63 ± 0.62 s and 72 ± 0.85 s at 100 and 200 mg/kg, respectively, indicating moderate anxiolytic activity comparable to diazepam. In the elevated plus maze, treatment with the extract significantly increased the time spent in open arms and reduced entries into closed arms at doses of 100 and 200 mg/kg, with values of 90.7 ± 0.64 s and 80.0 ± 1.08 s, respectively, compared with the control group (110.7 ± 0.70 s), further supporting anxiolytic effects. In the tail suspension test, the extract demonstrated marked antidepressant activity by significantly reducing immobility time from 190.8 ± 0.75 s in control mice to 93 ± 0.47 s and 78 ± 1.3 s at 100 and 200 mg/kg, respectively (*p* < 0.05), although the effect was less pronounced than that of imipramine (60 mg/kg, 30.2 ± 0.64). In summary, this evidence indicates that the methanolic extract of *H. annuus* seeds exerts significant CNS-stimulating and antidepressant effects, along with moderate anxiolytic activity, without evidence of neurotoxicity in the tested animal models [116].

### 5.16. Cytotoxic and Antitumor Activity

The dichloromethane flower extract of *H. angustifolius* demonstrated strong in vitro cytotoxic activity against human cancer cell lines CCRF-CEM leukemia cells, achieving potent inhibition at a concentration of 10 μg/mL, reducing cell viability to approximately 0.2% of the vehicle-treated control [61].

According to Singh et al. and Al-Snafi, the chloroform root extract of *H. annuus* displayed marked in vitro antiproliferative activity against human cancer cell lines, including MCF-7 (breast adenocarcinoma), A-431 (epidermoid carcinoma), and HeLa (cervical carcinoma) cell lines. The extract showed MIC_50_ values of 3.36 μg/mL for MCF-7, 4.19 for A-431, and 3.51 μg/mL for HeLa cell lines, indicating significant cytotoxic efficacy at low concentrations against diverse epithelial cancer cell lines [75,76].

In a study conducted by Alexandrino et al., the phenolic-rich crude extracts obtained from *H. annuus* seeds were evaluated in vitro for their antiproliferative and DNA-protective effects. Both ethanolic and sodium bisulfite extracts were analyzed. DNA-protective activity was assessed using a plasmid DNA oxidative damage model induced by the peroxyl radical generator AAPH. Both extracts demonstrated the ability to protect supercoiled DNA from oxidative strand breakage; however, only the ethanolic extract exhibited pronounced DNA-protective activity without evidence of cytotoxicity in cell proliferation assays. Furthermore, the ethanolic extract provided substantial protection against AAPH-induced DNA oxidative damage, preserving 89% of the DNA compared with 52% protection observed for the sodium bisulfite extract [106]. At a concentration range of 0.25–250 μg/mL, neither extract exhibited direct antiproliferative activity against several human tumor cell lines and one non-tumor human cell line, as cell growth remained above the cytotoxic threshold, in contrast to the positive control doxorubicin. These results indicate that the sunflower seed phenolic extracts did not exert cytotoxic effects on cell proliferation under the tested conditions [106]. These findings suggest that although *H. annuus* seed phenolic extracts do not directly inhibit tumor cell proliferation, they may contribute to chemopreventive effects through antioxidant-mediated DNA protection, highlighting their potential role in preventing oxidative DNA damage associated with carcinogenesis [106].

### 5.17. Photoprotective Activity

The ethanolic extract of *H. annuus* flowers (HAF) exhibited notable protective effects against UVB-induced photoaging in human dermal fibroblasts (NHDFs) in vitro [117]. NHDFs were exposed to UVB irradiation (144 mJ/cm^2^) and subsequently treated with HAF (1–100 μg/mL). HAF significantly reduced the generation of reactive oxygen species (ROS) and the expression of matrix metalloproteinases (MMP-1 and MMP-3), while maintaining normal levels of type I procollagen. Mechanistically, these protective effects were associated with the activation of the Nrf2 pathway, promoting its nuclear translocation, upregulation of TGF-β1 expression, and inhibition of AP-1 and MAPK signaling cascade.

Furthermore, HAF suppressed UVB-induced secretion of vascular endothelial growth factor (VEGF) and pro-inflammatory mediators, including IL-6, COX-2, iNOS, and TNF-α, indicating a potential modulatory role in NFAT-dependent inflammatory signaling. Complementing these findings, HAF demonstrated potent antioxidant activity, with IC_50_ values of 72.65 μg/mL and 174.52 μg/mL for DPPH and ABTS radical scavenging assays, respectively. These results suggest that *H. annuus* flower extract may serve as a promising natural agent for the prevention of oxidative stress and photoaging-related skin damage [117].

### 5.18. Choleretic and Cholekinetic Activity

Dababneh investigated the phytochemical composition of the dry extract of sunflower heads (*H. annuus*; DESH) and its potential choleretic and cholekinetic activities in vivo using a complex experimental model of toxic liver injury, focusing on changes in bile secretion processes under pathological conditions. Hepatobiliary pathology was induced by combined administration of carbon tetrachloride (CCl_4_) and ethanol, and DESH was administered orally in a therapeutic–prophylactic regimen at doses ranging from 15 to 100 mg/kg. The results showed that administration of DESH at doses of 75 and 100 mg/kg significantly increased bile secretion rate (BSR) by 1.7-fold and 1.5-fold, respectively, compared to the control group (*p* < 0.05). Additionally, at these doses, DESH normalized bile acid synthesis, resulting in a 1.4-fold increase (*p* < 0.05), and also improved biliary cholesterol content with increases of 1.7-fold and 1.5-fold, respectively (*p* < 0.05) [118]. These observations indicate that DESH exhibits significant biliary-synthetic and choleretic activity at effective doses between 75 and 100 mg/kg, supporting its potential use as an adjuvant phytotherapeutic agent in the management of hepatobiliary disorders associated with cholestasis.

Table S6 summarizes diverse biological activities reported for *Helianthus* species, including immunomodulatory and anti-asthmatic effects, anti-atopic and anti-inflammatory activities, central nervous system modulation, cytotoxic and antiproliferative effects, photoprotective actions, and choleretic activity, as supported by in vitro and in vivo experimental models (Levels A–C). While these studies highlight a broad pharmacological spectrum, most rely on crude extracts.

## 6. Biological Activity of Terpenoid Compounds Isolated from *Helianthus*

### 6.1. Anti-Inflammatory Activity and Atherosclerosis Prevention

Three diterpene acids (kaurenoic acid, trachylobanoic acid, and grandifloric acid) obtained from a petroleum ether extract of *H. annuus* flower heads were assessed for anti-inflammatory activity using in vitro and in vivo models. In LPS-activated RAW 264.7 macrophages, all compounds reduced nitric oxide (NO), prostaglandin E_2_ (PGE_2_), and TNF-α production in a concentration-dependent manner at non-cytotoxic concentrations (1–20 μM), as well as suppressed the expression of inducible nitric oxide synthase (NOS-2) and cyclooxygenase-2 (COX-2). In addition, these compounds exhibited significant in vivo anti-inflammatory effects in a 12-*O*-tetradecanoylphorbol-13-acetate (TPA)-induced mouse ear edema model. Topical application of diterpenoids significantly reduced ear edema in mice induced by TPA, comparable to indomethacin. Myeloperoxidase (MPO) activity was also significantly reduced by pretreatment with diterpenoids [75,76].

The anti-inflammatory potential of six triterpene glycosides isolated from a methanol extract of sunflower petals (*H. annuus*) was investigated. Helianthosides 1, 2, 3, 4, 5, and helianthoside B were obtained from the *n*-butanol–soluble fraction and were evaluated in vivo using a mouse TPA–induced mouse ear edema model (1.7 nmol/ear). All tested compounds exhibited significant anti-inflammatory effects, with ID_50_ (50% inhibitory dose) values ranging from 65 to 262 nmol/ear. Notably, all helianthosides were more potent than indomethacin (ID_50_ = 838 nmol/ear). Among them, helianthoside B displayed the strongest inhibitory activity, with potency comparable to that of hydrocortisone (ID_50_ = 83 nmol/ear) [75,76].

Heliangin, isolated from Jerusalem artichoke (*H. tuberosus*) leaf ethanol extract, showed anti-inflammatory activity in vitro. Heliangin (2–25 μM) significantly inhibited the production of NO in macrophage-like RAW 264.7 cells induced by LPS. Additionally, heliangin (15 μM) exhibited suppression of ICAM-1, VCAM-1, E-selectin, and MCP-1 expression by inhibiting NF-κB and IκBα phosphorylation in vascular endothelial cells when stimulated with TNF-α. These data indicate that heliangin modulates macrophage and endothelial inflammatory responses relevant to atherosclerosis, although evidence remains limited to cellular models [65].

### 6.2. Antiviral Activity

Six rearranged 3,4-seco-tirucallane-type triterpenoids were isolated from the diethyl ether extract of sunflower pollen grains (*H. annuus*). These triterpenoids include Sunpollenol, (24R)-24,25-Epoxysunpollenol, (24S)-24,25-Epoxysunpollenol, (23E)-23-Dehydro-25-hydroxysunpollenol, (24R)-24,25-Dihydroxysunpollenol, and (24S)-24,25-Dihydroxysunpollenol. Their ability to inhibit Epstein-Barr virus early antigen (EBV-EA) activation, which is induced by the tumor promoter 12-O-tetradecanoylphorbol-13-acetate (TPA) in Raji cells, was evaluated in vitro. All compounds demonstrated potent inhibitory effects on EBV-EA activation, achieving 97–100% inhibition at a molar concentration of 1 × 10^3^ relative to TPA [119].

### 6.3. Antiparasitic Activity

The antiplasmodial activity of both the methanol extract and ethyl acetate fraction obtained from *H. annuus* leaves against the *P. falciparum* 3D7 strain was investigated in vitro at concentrations ranging from 0.01 to 100 µg/mL by Mutiah et al. [120]. The methanolic extract exhibited an IC_50_ value of 22.18 µg/mL, whereas the ethyl acetate fraction showed a lower IC_50_ value of 16.68 µg/mL; according to the Gessler criteria, both samples can be classified as having good in vitro antiplasmodial activity. UPLC–MS analysis of the ethyl acetate fraction revealed the presence of compounds such as heliangolide, artemisinin, and eupalinolide C. However, individual compounds were not isolated nor directly evaluated for antimalarial activity.

The STL 4,15-iso-atriplicolide tiglate, isolated from the dichloromethane extract obtained from *H. tuberosus* aerial parts, demonstrated significant antitrypanosomal activity in vitro with an IC_50_ value of 15 nM against *Trypanosoma brucei rhodesiense* (IC_50_ value 0.015 µM) [64]. The activity against *T. cruzi* and *P. falciparum* was also reported (IC_50_ values 3.7 µM and 1.0µM, respectively) [64].

### 6.4. Cytotoxic Activity

Sesquiterpene lactones with cytotoxic activity have been identified in *Helianthus* species. Among them, germacranolides and furanosheliangolides such as desacetoxyeupaserrin and desacetyleupaserrin are notable for their antileukemic effects [22]. The STL 4,15-iso-aripliciolide tiglate, isolated from the ethyl acetate extract of *H. tuberosus* leaves, exhibited pronounced cytotoxic activity against human cancer cell lines. Its growth-inhibitory effects were evaluated in vitro using the MTT assay after 48 h of treatment at concentrations ranging from 0.1 to 20 µg/mL. 4,15-iso-aripliciolide tiglate showed significant growth inhibitory activity against MCF-7, A549, and HeLa cancer cells lines with IC_50_ values of 1.97 ± 0.04, 7.79 ± 0.44, and 9.87 ± mg/mL, respectively [62]. Structure–activity relationship analysis indicated that FHL-type STLs were more cytotoxic than guaianolide-type, with activity influenced by the configuration and substitution pattern of the conjugated ester side chain. In particular, the presence of an exocyclic double bond at C-4 and a *cis*-configured ester group would contribute to the enhanced cytotoxic potency of 4,15-iso-aripliciolide tiglate [62].

STL 8-methacrylyl-4,15-iso-atriplicolide, isolated from the dichloromethane extract of *H. angustifolius* flowers, showed significant in vitro cytotoxic activity against human cancer cell lines (CCRF-CEM leukemia, MDA-MB-231 breast cancer, U251 glioblastoma, HCT 116 colon cancer, and human lung fibroblast cell line MRC-5). Cytotoxicity-guided fractionation followed by XTT viability assays revealed IC_50_ values of 0.26 ± 0.01 μM for CCRF-CEM cells, 3.08 ± 0.15 µM for MDA-MB-231 cells, 10.17 ± 1.60 µM for U251 cells, 1.02 ± 0.09 µM for HCT 116 cells, and 4.22 ± 0.26 μM for MRC-5 cells [61]. Structure–activity relationship analysis suggested that the α-olefine-γ-lactone (Michael acceptor) moiety and the exocyclic methylene group at C-15 contribute to enhanced cytotoxicity. The presence of an additional α,β-unsaturated carbonyl group in 8-methacrylyl-4,15-iso-atriplicolide may further enhance alkylating potential and DNA cross-linking, leading to higher cytotoxicity [61].

### 6.5. Antioxidant and Antidiabetic Activity

The antioxidant and antidiabetic properties of 20-dehydroeucannabinolide, a derivative of the sesquiterpene lactone heliangolide, isolated from the hydromethanolic extract of *H. annuus* leaves, were investigated. The compound was purified through successive solvent–solvent partitioning followed by column chromatography, thin-layer chromatography, and preparative high-performance liquid chromatography. Antioxidant activity was assessed in vitro using DPPH and nitric oxide radical scavenging assays, while antidiabetic effects were evaluated in vivo in alloxan-induced diabetic albino Wistar rats. In experiments with diabetic rats, oral administration of the heliangolide derivative (200 mmol kg^−1^) significantly (*p* < 0.05) reduced fasting blood glucose (FBG) levels, demonstrating effects comparable to those of glibenclamide (4 mmol kg^−1^) within the first 6 h post-treatment. Additionally, in assays for nitric oxide and DPPH radical scavenging, the compound showed inhibition rates of 26.0% and 23.7%, respectively, at a concentration of 954.2 μmol/L, whereas ascorbic acid, which was used as a control in the study, exhibited higher inhibitory effects (96.6% and 50.9%, respectively) at 2271.2 µmol/L [121].

### 6.6. Antimicrobial Activity

From the acetone extract of *H. debilis* subsp. *cucumerifolius* leaves, the FHL 17,18-dihydrobudlein A was isolated and evaluated in vitro for antimicrobial activity. This compound demonstrated potent antimicrobial activity in agar diffusion assays against *Bacillus brevis*, with a minimum inhibitory concentration (MIC) of 16 ng/mL [22].

In another study, t glandulon A, B, and C, bizabolene-type sesquiterpenes identified in *H. annuus*, were also reported to display antimicrobial effects. These compounds demonstrated antimicrobial activity by exhibiting a cytostatic in vitro activity against *B. brevis* in agar diffusion tests [22].

Table S7 provides an overview of the biological activities reported for terpenoid compounds isolated from *Helianthus* species, based on the available literature. The diterpenes kaurenoic, trachylobanoic, and grandifloric acids from *H. annuus* exhibited anti-inflammatory effects both in vitro and in vivo. The triterpene glycoside helianthoside B showed potent anti-inflammatory activity in vivo, surpassing indomethacin and approaching the efficacy of hydrocortisone. Among STLs, heliangin from *H. tuberosus* demonstrated notable anti-inflammatory and anti-atherosclerotic potential. The STL 4,15-iso-atriplicolide tiglate emerged as a particularly promising lead, displaying strong antitrypanosomal activity at submicromolar concentrations, along with significant cytotoxic effects against multiple human cancer cell lines. Similarly, 8-methacrylyl-4,15-iso-atriplicolide showed high potency and selectivity against leukemia and solid tumor cell lines. In addition, 3,4-seco-tirucallane-type triterpenoids from *H. annuus* pollen exhibited potent antiviral activity against EBV without detectable cytotoxicity, while 17,18-dihydrobudlein A showed remarkable antibacterial activity at ng/mL concentrations.

## 7. Biological Activity of Flavonoids and Phenolic Compounds Isolated from *Helianthus* Species

### 7.1. Antioxidant Activity

Jerusalem artichoke (*H. tuberosus*) leaves were analyzed in vitro for their antioxidant potential through the determination of total phenolic content and radical scavenging activities to assess their potential as a source of natural antioxidants. Dried leaves were extracted with 60% ethanol, followed by solvent–solvent partitioning to obtain petroleum ether, ethyl acetate, n-butanol, and aqueous fractions. Total phenolic content was quantified using the Folin–Ciocalteu method, while antioxidant activity was evaluated using DPPH, ABTS^+^, and hydroxyl radical scavenging assays, with BHT as a positive control. Among all fractions, the ethyl acetate fraction exhibited the highest phenolic content (266.69 ± 2.51 mg GAE/g dry extract) and demonstrated a strong capacity to scavenge free radicals. Six phenolic compounds with high efficacy in neutralizing free radicals were isolated from this fraction. Notably, 3-O-caffeoylquinic acid and 1,5-dicaffeoylquinic acid were identified as major contributors due to their potent radical scavenging abilities and high concentrations. The content of 3-O-caffeoylquinic acid reached 74.58 ± 1.05 mg/g in the n-butanol fraction, whereas 1,5-dicaffeoylquinic acid was most abundant in the ethyl acetate fraction (104.51 ± 2.86 mg/g), supporting their key role in the observed antioxidant effects [122]. Analysis of structure–activity relationships revealed that the caffeoyl moiety represents the key functional group responsible for the observed radical scavenging activity of the phenolic compounds isolated from *H. tuberosus* leaves. Compounds containing two caffeoyl units exhibited enhanced antioxidant activity compared to monocaffeoyl derivatives, whereas variations in the position of the caffeoyl groups on the quinic acid skeleton exerted only a limited effect. In the DPPH, ABTS^+^, and hydroxyl radical scavenging assays, dicaffeoylquinic acid isomers showed comparable activities. Notably, all isolated phenolics were more active than the crude extract and solvent fractions, indicating that these compounds are primarily responsible for the antioxidant capacity of Jerusalem artichoke leaves [122].

In a study conducted by Alexandrino et al., the antioxidant properties of phenolic compounds extracted from defatted *H. annuus* seeds flour using aqueous ethanolic (70%, *v*/*v*) and sodium bisulfite solutions were investigated. Total phenolic contents were quantified as chlorogenic acid equivalents (CGA) using the Folin–Ciocalteu method. Both extracts demonstrated antioxidant activity, yielding15.44 g CGA eq/100 g for the ethanolic extract and 11.57 g CGA eq/100 g for the sodium bisulfite extract. Antioxidant activity was evaluated in vitro by complementary radical scavenging and oxygen radical absorbance assays (DPPH, ABTS, and ORAC), with both extracts exhibiting significant antioxidant capacity; however, the ethanolic extract was consistently more active, displaying a lower DPPH EC_50_ value (0.36 g extract/g DPPH•) and higher ORAC values. Chlorogenic acid content was determined by HPLC-DAD at 324 nm, identifying it as the predominant phenolic compound, constituting approximately 62% of the total phenolic content (2.46 ± 0.11 g/100 g, dry basis). These findings indicate that sunflower seed phenolic compounds, particularly chlorogenic acid, are the main contributors to the observed antioxidant activity, likely due to their multiple hydroxyl groups that favor radical scavenging mechanisms [106].

The methanolic extract of *H. tuberosus* tubers was fractionated into n-hexane and ethyl acetate fractions, which were subsequently evaluated for their antioxidant and antidiabetic activities. Among them, the ethyl acetate fraction exhibited the highest total phenolic and flavonoid contents as determined by the Folin–Ciocalteu method (69.55 ± 0.36 mg gallic acid equivalent/g and 21.03 ± 0.97 mg quercetin equivalent/g, respectively). Consistently, this fraction showed the strongest in vitro antioxidant activity in both DPPH● and ABTS^+^ radical scavenging assays, with IC_50_ values of 161.55 ± 0.98 μg/mL and 104.45 ± 3.01 μg/mL, respectively, compared with ascorbic acid (IC_50_ = 48.84 ± 2.04 μg/mL). In addition, the ethyl acetate fraction displayed marked antidiabetic potential, as evidenced by its superior inhibitory activity against α-amylase and α-glucosidase compared with the other fractions. The IC_50_ values for α-glucosidase inhibition were 187.04 ± 0.42 μg/mL for the ethyl acetate fraction, 377.88 ± 1.63 μg/mL for the hexane fraction, <1000 μg/mL for the methanolic fraction, and 723.58 ± 0.15 μg/mL for the crude methanolic extract, whereas acarbose showed an IC_50_ of 64.62 ± 0.59 μg/mL. Similarly, the ethyl acetate fraction exhibited the most pronounced inhibitory activity for α-amylase (IC_50_ = 102.53 ± 1.39 μg/mL). These results indicate that the ethyl acetate fraction effectively inhibits key carbohydrate-hydrolyzing enzymes, potentially slowing polysaccharide breakdown, lowering blood glucose levels, and reducing postprandial hyperglycemia. Phytochemical analysis of the ethyl acetate fraction by UPLC-QTOF-MS-MS and GC/MS led to the identification of several phenolic compounds, including neochlorogenic acid, chlorogenic acid, caffeoylquinic acid, caffeic acid, 5-O-(4-coumaroyl)-quinic acid, dicaffeoylquinic acid isomers, 1,4-dicaffeoylquinic acid, feruloylquinic acid, β-D-glucoside of salicylic acid, and isomers of salvianolic acid derivatives. These phenolic phytocompounds were proposed to be responsible for the antioxidant and antidiabetic effects observed for the ethyl acetate fraction [97].

Wang et al. investigated the antioxidative properties of flavonoids isolated from Jerusalem artichoke leaves using in vitro radical scavenging assays. The flavonoids 5,8-diOH-6,7-diMeO-2-(3,4-diMeOPh)-4-benzopyrone and 5,8-diOH-6,7,40-triOMe (pedunculin) were evaluated at concentrations ranging from 0.5 to 200 µg/mL using DPPH●, ABTS^+^, and hydroxyl radical scavenging assays, with butylated hydroxytoluene (BHT) as the positive control. Both compounds exhibited dose-dependent radical scavenging activity in all assays. In the DPPH assay, SC_50_ values of 90.61 ± 0.59 µg/mL and 106.80 ± 0.85 µg/mL were obtained for the flavonoids, respectively, compared with 103.30 ± 0.76 µg/mL for BHT. Notably, both flavonoids showed markedly superior activity in the ABTS^+^ assay (SC_50_ = 1.40 ± 0.06 and 1.31 ± 0.11 µg/mL, respectively) relative to BHT (SC_50_ = 5.13 ± 0.21 µg/mL), as well as enhanced hydroxyl radical scavenging capacity (SC_50_ = 15.07 ± 0.56 and 10.61 ± 0.31 µg/mL, respectively, vs. 46.62 ± 0.35 µg/mL for BHT). These results evidence that Jerusalem artichoke leaves are a rich source of highly active antioxidant flavonoids with greater radical scavenging potency than a conventional synthetic antioxidant, supporting their potential use as natural antioxidants for mitigating oxidative stress–related disorders, although in vivo validation is still required [123]. This activity is consistent with a structure–activity relationship in which free phenolic hydroxyl groups play a key role in radical scavenging.

### 7.2. Choleretic and Hepatoprotective Activity

According to the study conducted by Dababneh in 2021, the choleretic and biliary effects of the dry extract of *H. annuus* heads (DESH) are likely attributable to its phytochemical composition, particularly flavonoids such as quercetamethrin and the coumarin glycoside scopolin present in the extract [118]. The observations indicate that DESH exerts dose-dependent choleretic and hepatoprotective effects in vivo, likely mediated by the synergistic action of flavonoids and coumarin derivatives, which may contribute to improved bile formation and secretion under cholestatic conditions [118].

### 7.3. Anti-Obesity Effect

In a randomized, double-blind, placebo-controlled pilot study, the effects of a standardized sunflower (*H. annuus*) seed extract, containing 40% chlorogenic acids, on body weight and body composition in obese adults were investigated. Fifty participants (BMI 30–40 kg/m^2^) were randomly assigned to receive either 500 mg/day of sunflower seed extract or a placebo (2 capsules/day) for 12 weeks, in combination with a hypocaloric diet. Each capsule contained 250 mg of extract standardized by HPLC, containing mono- and dicaffeoylquinic acids, including 3-, 4-, 5-caffeoylquinic acids, as well as 3,4- 3,5-, and 4,5-di-caffeoylquinic acids, with 5-caffeoylquinic acid as the major constituent. The results indicated a grater reduction in body weight in the sunflower extract group (6.8%) compared to the placebo group (5.7%). Sunflower extract consumption for 12 weeks resulted in a significant reduction in Body Mass Index (BMI) (−2.60 vs. −1.88; *p* = 0.02), and waist circumference (−8.44 vs. −4.75 cm; *p* = 0.001) compared to placebo, with particularly notable improvements observed among obese women over 30 years old [87]. Overall, this human study provides preliminary clinical evidence that chlorogenic acid–rich sunflower seed extract exerts beneficial effects on body weight regulation, fat mass reduction, and lipid profile, supporting its potential use as a natural adjunct in obesity management. Recently, another study also demonstrated that sunflower seed extract may represent a promising and well-tolerated option for the management of obesity [55].

Table S8 provides a compiled overview of the biological activities reported for flavonoids and phenolic compounds isolated from *Helianthus* species, based on the evaluated literature. Among the phenolic compounds, caffeoylquinic acid derivatives stand out as the main contributors to antioxidant and metabolic effects. In *H. tuberosus*, mono- and dicaffeoylquinic acids, particularly 1,5-dicaffeoylquinic acid, exhibited strong in vitro radical scavenging activity. In *H. annuus*, chlorogenic acid was identified as the predominant phenolic constituent determinant of antioxidant capacity. Notably, polymethoxylated flavonoids from *H. tuberosus* leaves showed antioxidant activities comparable to or superior to the synthetic standard BHT across multiple assays.

## 8. Other Secondary Metabolites Isolated from *Helianthus*

### 8.1. Other Compounds Identified or Isolated from Helianthus Species

Beyond terpenoids and phenolic compounds, the genus *Helianthus* has been reported to contain additional classes of secondary metabolites, mainly identified in *H. annuus*. Recent computational studies identified a complex profile of alkaloids in the receptacles of *H. annuus*, with 231 alkaloid molecules detected by LC–MS; among them, 2-naphthylalanine, medroxalol, and fenspiride were highlighted as potential xanthine oxidase inhibitors with relevance for gout management [124].

Sunflower seed oil is also a rich source of tocopherols, mainly α-, β-, γ-, and δ-tocopherol, with α-tocopherol being the most biologically relevant isomer [76,86]. In addition, phytosterols such as β-sitosterol, stigmasterol, and campesterol have been isolated from *H. annuus* seeds [125]. Moreover, sunflower oil was found to be particularly rich in linoleic acid (C18:2), accounting for approximately 55.7% of its fatty acid composition, thereby contributing to its nutritional and functional properties [126].

Other relevant constituents include polysaccharides, particularly inulin, which is abundant in the tubers of *H. tuberosus* (Jerusalem artichoke) [127].

A summary of the reported compounds and their sources is presented in Table 5.

Besides secondary metabolites, proteins have also been described in *H. annuus*, including helianthinin, albumins, and bioactive proteins such as a lipase inhibitor and the 16-kDa protein SAP16. Extraction and characterization studies indicate that sunflower proteins display functional and potentially biological properties, emphasizing their relevance beyond nutritional aspects. In particular, optimized aqueous extraction conditions have been shown to significantly affect protein yield, solubility, and functional performance, with sunflower protein isolates exhibiting good solubility over a wide pH range as well as foaming and emulsifying capacities [128,129].

### 8.2. Biological Activity of Other Compounds Isolated from Helianthus

Among the emerging pharmacological applications associated with *H. annuus*, gout has recently gained attention as a relevant therapeutic target. A recent computational study combined LC–MS profiling with advanced in silico approaches to investigate alkaloids extracted from *H. annuus* receptacles against gout. Using a novel clustering strategy—TriDimensional Hierarchical Fingerprint Clustering with Tanimoto Representative Selection (3DHFC-TRS)—231 alkaloid molecules from *H. annuus* receptacles were analyzed, leading to the identification of six clusters with potential anti-gout relevance. Molecular docking, BatchDTA affinity prediction, and molecular dynamics simulations highlighted 2-naphthylalanine, medroxalol, and fenspiride as the most promising XO inhibitors, with medroxalol proposed as a novel candidate for gout management [124].

Tocopherols are among the major bioactive constituents of sunflower oil and play a central role in its antioxidant capacity. These compounds act as chain-breaking antioxidants by scavenging lipid peroxyl radicals, thereby protecting cellular membranes and lipoproteins from oxidative damage both in vitro and in vivo. Four main tocopherol isomers (α-, β-, γ-, and δ-tocopherol) have been identified, differing in their antioxidant efficacy, with α-tocopherol exhibiting the highest biological potency. Through their antioxidant activity, tocopherols contribute to the maintenance of redox homeostasis and have been associated with a reduced risk of oxidative stress-related disorders, including cardiovascular diseases and certain types of cancer. As vitamin E cannot be synthesized endogenously, dietary sources such as sunflower oil represent an important means of ensuring adequate intake [86]. Beyond their antioxidant function, vitamin E and magnesium present in sunflower contribute to anti-inflammatory effects and have been associated with a reduced risk of several oxidative stress–related and inflammatory conditions, including cardiovascular diseases, osteoarthritis, rheumatoid arthritis, asthma, and hypertension. Additionally, tocopherols, together with phytosterols, exert hypocholesterolemic effects by lowering LDL and plasma cholesterol levels and by inhibiting intestinal cholesterol absorption, reinforcing the cardioprotective potential of sunflower-derived products [76].

In addition, phytosterols from *Helianthus annuus* have demonstrated neurite outgrowth–promoting activity in PC12 cells in vitro, an effect associated with the enhancement of nerve growth factor (NGF) signaling, which is relevant in the context of neurodegenerative disorders such as Alzheimer’s disease. Bioassay-guided fractionation of sunflower seed extracts led to the identification of a phytosterol-rich fraction composed mainly of β-sitosterol, along with stigmasterol and campesterol. Among these compounds, β-sitosterol and stigmasterol exhibited the strongest neurite outgrowth–promoting activity. Further immunostaining analyses revealed that β-sitosterol–induced neurite formation was accompanied by increased neurofilament expression. These findings indicate that β-sitosterol is the principal contributor to the NGF-enhancing activity of sunflower seeds and may represent a promising neuroactive compound [125].

Inulin isolated from *H. tuberosus* has demonstrated significant antihyperglycaemic activity in vivo. In a high-fat diet and streptozotocin-induced hyperglycaemic mouse model, inulin supplementation reduced fasting blood glucose levels, body weight gain, liver weight, and circulating lipid parameters, including triacylglycerols and cholesterol. These metabolic improvements were accompanied by modulation of liver-related gene expression and a marked improvement in intestinal microbiota composition, particularly through an increase in *Bacteroides* abundance. This evidence supports inulin from *H. tuberosus* as a promising functional food ingredient for the prevention and management of hyperglycaemia [127].

The biological activities of other compounds isolated from *Helianthus* are summarized in Table S9.

## 9. Discussion

This review provides a comprehensive analysis of the available scientific data on 66 species within the genus *Helianthus*. It covers the taxonomic classification of *Helianthus* species according to the IPNI database and highlights their geographic distribution. Native to North America, these species are most diverse in the United States. Among these species, *H. annuus* L., commonly known as sunflower, stands out due to its significant economic and cultural importance. This species has garnered widespread recognition and utilization, which has driven extensive research interest, further highlighting the genus’ relevance in various sectors.

Despite the wide variety of species in the *Helianthus* genus, *H. annuus* and *H. tuberosus* are the most used. Ethnobotanical evidence highlights *H. annuus* as a multifunctional medicinal and nutritional species, traditionally used across cultures for respiratory, digestive, dermatological, and circulatory ailments, supporting its relevance as a source of bioactive compounds for therapeutic applications. *H. tuberosus* is valued for its nutritional and medicinal uses too, particularly in diabetes management and digestive health, consistent with its inulin-rich composition and bioactive properties that support metabolic and gastrointestinal benefits. Overall, *H. annuus* emerges as a culturally versatile medicinal plant with a wide but heterogeneous range of traditional applications, whereas *H. tuberosus* represents a more specialized species whose ethnomedical relevance is tightly linked to its nutritional composition and metabolic benefits.

An update on the terpenic compounds reported up to the end of 2024 reveals the isolation of more than 119 terpenic compounds in the *Helianthus* genus. Among the STLs, compounds such as budlein A, niveusin A, niveusin B, niveusin C, eupasserin, pinnatifidin 1,2-dihydroxy, tifruticin desoxy-3-dehydro-15-hydroxy, atripliciolide angelate, costunolide 8-14-dihydroxy, along with their natural derivatives, as well as mollisorin B and its natural diastereoisomers, have been reported in several *Helianthus* species. Additionally, the diterpenoids *ent*-12,16-cyclokaurenoic acid, *ent*-kaurenoic acid, and *ent*-12 β -acetoxykaurenoic acid were also reported in several species.

Of the one hundred STLs summarized in Table 2, sixty-two were identified by comparison with authentic reference standards using chromatographic techniques. Most of the studies reporting these identifications were published during the 1990s, a period in which classical chromatographic approaches were widely applied and considered reliable. Although these methodologies provided a solid foundation for compound identification, complementary analyses using contemporary analytical tools, such as LC–MS/MS and advanced NMR techniques, could further strengthen the structural assignments and enhance comparability with more recent phytochemical studies.

Studies investigating STLs in *Helianthus* species have employed both microsampling approaches and bulk extraction methodologies. Microsampling techniques, often based on the extraction of glandular trichomes, are particularly informative for chemotaxonomic and ecological studies, as they target sites of localized STL biosynthesis and accumulation. In contrast, bulk extraction of whole organs or tissues yields an integrated chemical profile but may dilute tissue-specific metabolites such as STLs. Consequently, differences in extraction scale and strategy can substantially influence STL detection and the reported extent of chemical diversity. These methodological differences should therefore be considered when comparing STL profiles across species and studies. Notably, of the one hundred STLs listed in Table 2, eighty-seven were obtained through microsampling techniques, underscoring the importance of this approach for capturing STL diversity within the genus. Conversely, the use of large amounts of plant material remains appropriate for purposes other than chemotaxonomy, such as the isolation and structural characterization of individual compounds.

In future phytochemical studies of *Helianthus*, a standardized analytical workflow is recommended, encompassing well-documented sampling with deposited voucher specimens and complete metadata (geographic origin, plant organ, developmental stage, and extraction strategy), the strategic use of microsampling for chemotaxonomic and ecological analyses versus bulk extraction for compound isolation, comprehensive LC–HRMS profiling coupled with MS/MS for structural annotation, and, where feasible, confirmation by NMR spectroscopy using authentic reference compounds. Adoption of such a workflow would substantially improve data quality, facilitate genus-wide comparisons, and support integrative studies linking chemistry, ecology, and applied research in *Helianthus*.

Taken together, STLs constitute the most diverse terpenoid class within the *Helianthus* genus, with *H. annuus* displaying the greatest structural diversity, likely reflecting both its ecological relevance and the intensity of phytochemical investigation. Other species, such as *H. tuberosus* and *H. angustifolius*, exhibit more restricted but biologically relevant STL profiles, often dominated by specific germacranolide or heliangolide derivatives. Diterpenes, particularly kaurane- and trachylobane-type acids, are widely distributed across the genus, indicating conserved biosynthetic pathways, while triterpenes appear less diverse and are mainly reported from *H. annuus*. These patterns highlight *H. annuus* as the chemically most complex species, while also underscoring the untapped terpenoid diversity of less-studied *Helianthus* taxa.

Sesquiterpene lactones are well recognized for their central role in plant defense, contributing to resistance against herbivores and pathogens, as well as mediating allelopathic interactions with competing plant species.

Allelopathy is a biochemical interaction in which a plant influences neighboring organisms through the release of chemical compounds into the environment. In this sense, STLs have been widely reported to exhibit allelopathic activity. Within the genus *Helianthus*, several STLs have been identified as allelochemicals in *H. annuus*, including helivypolide D, leptocarpin, helivypolide E, annuolides F and H, helivypolides F, H, and J, helieudesmanolide A, and 8β-angeloyloxycumambranolide. Similarly, allelopathic activity has been reported for *H. tuberosus*, notably for 1,10-epoxidized heliangolides, 1-keto-2,3-unsaturated furanoheliangolides, 4,15-isoatriplicolide angelate, and 4,15-isoatriplicolide methylacrylate. The production of these allelochemicals may enhance ecological fitness by reducing interspecific competition through the inhibition of neighboring plant establishment, thereby conferring a competitive advantage in resource-limited environments [130].

A broad spectrum of biological activities has been attributed to STLs, encompassing antibacterial, antifungal, and effects against both invertebrate and vertebrate organisms. These effects are largely associated with the presence of α,β-unsaturated carbonyl groups, which readily interact with biological macromolecules via Michael-type addition reactions, often resulting in enzyme inhibition. Additionally, the stereochemical configuration of the STL ring has been shown to affect herbivore resistance, with plants producing *cis*-fused STLs generally suffering greater herbivore damage than those producing trans-fused lactones [131].

Flavonoid profiles in the *Helianthus* genus combine conserved structural features with marked species-specific diversification. Flavones and flavonols predominate, with methoxylated flavones and common flavonol glycosides recurrently reported across species; representative compounds include nevadensin, hispidulin, nepetin, jaceosidin, isoquercetin, hymenoxin, coreopsin, sulfuretin, acerosin, and quercetin-7-O-glucoside. *H. annuus* exhibits the greatest structural diversity, encompassing aurones, chalcones, and flavones localized in GT, whereas *H. tuberosus* is characterized mainly by glycosylated flavonols and phenolic acids consistent with its nutritional and functional uses. Wild *Helianthus* species further expand the genus’ chemical space through a more restricted but taxonomically informative distribution of chalcones and aurones.

Most phytochemical and pharmacological studies have focused on *H. annuus* and *H. tuberosus*, allowing the identification of several associations between chemical composition and biological activity. These species exhibit a high chemical diversity, encompassing a wide range of STL skeletons—including guaianolides, germacranolides, heliangolides, furanoheliangolides, and eudesmanolides (Table 2)—as well as diverse flavonoid subclasses and phenolic acids (Table 3). Species rich in STLs are more frequently associated with anticancer, anti-inflammatory, antimicrobial, and antiprotozoal activities, whereas flavonoid-rich species and extracts are predominantly linked to antioxidant effects. Diterpenes, although less widely distributed within the genus, have also been implicated in anti-inflammatory and antiviral activities, particularly in *H. annuus*. These trends are supported by the biological activity data summarized in the Supplementary Materials in Tables S2–S6 (activities reported for *Helianthus* species) and Tables S7 and S8 (activities of terpenoid and phenolic compounds isolated from *Helianthus* species). These observations highlight clear interspecific differences in both chemical diversity and biological activity within the genus.

More than twenty distinct biological activities have been reported for *H. annuus*, with the majority supported by in vitro assays (≈26 reports), reflecting multiple experimental evaluations per activity using different plant parts, extracts, and models; followed by a substantial number of in vivo studies in animal models (≈21 reports), and very limited clinical evidence. In comparison, five biological activities have been described for *H. tuberosus*, mainly in vitro with a few in vivo reports, and one each in vitro biological activity for *H. salicifolius* and *H. angustifolius*. Among these, antidiabetic, antioxidant, antimicrobial, and anticancer activities are the most reported. Terpenoid compounds, including diterpenes and triterpene glycosides reported in *H. annuus* and the STL heliangin isolated from *H. tuberosus*, have shown anti-inflammatory activities [65,75,76]. Furthermore, 3,4-seco-tirucallane-type triterpenoids have been shown to possess antiviral activity against the Epstein–Barr virus [113]. In addition, compounds such as heliangolide, artemisinin, and eupalinolide C, reported in *H. annuus*, exhibit significant antiplasmodial effects [114]. Overall, the evidence presented highlights *H. annuus* as the most pharmacologically versatile species within the genus, while also underscoring the need for deeper bioactivity exploration of less-studied *Helianthus* taxa.

Several STLs exhibiting antitumor activity have been identified in *Helianthus*. Among them, germacranolides and furanosheliangolides such as desacetoxyeupaserrin and desacetyleupaserrin are particularly notable for their antileukemic effects [22]. The STL 4,15-iso-atripliciolide tiglate and 8-methacrylyl-4,15-iso-atriplicolide stand out for their cytotoxic effects against breast cancer, lung carcinoma, leukemia, glioblastoma, and colon cancer human cancer cell lines [62]. In addition to its anticancer properties, 4,15-iso-atriplicolide tiglate also exhibited significant activity against *Trypanosoma brucei rhodesiense* [64].

Among the STLs identified in *Helianthus* species, furanogermacranolides stand out as a group of particular interest. Comparative analyses suggest that furanogermacranolides tend to exhibit higher cytotoxicity than guaianolides and eudesmanolides, as reported for STLs isolated from *H. tuberosus* and *H. angustifolius*. Structural features appear to play a key role in modulating activity within this subclass of STLs. In particular, the presence of additional α,β-unsaturated carbonyl groups enhances cytotoxic potency, as compounds bearing unsaturated side chains showed higher activity than analogues with saturated substituents. A similar trend was observed for antiparasitic activity: furanogermacranolides isolated from *H. tuberosus* exhibited potent activity against protozoa, whereas a compound lacking an α,β-unsaturated carbonyl group displayed reduced efficacy. The enhanced activity associated with α,β-unsaturated carbonyl groups is commonly attributed to their ability to interact with multiple biological targets by acting as Michael acceptors. Overall, the available data emphasize furanogermacranolides as promising antitumor and antiparasitic agents within the *Helianthus* genus. However, given the limited number of compounds and biological assays, further systematic studies are required to establish robust structure–activity relationships across the different STL classes found in the genus. Further investigations have highlighted the potential of heliangolide derivatives, such as 20-dehydroeucannabinolide, which display both antioxidant and antidiabetic activities [115]. Additionally, the FHL 17,18-dihydrobudlein A, isolated from *Helianthus debilis* subsp. *cucumerifolius* leaves demonstrated potent antimicrobial activity against *B. brevis* [22]. Lastly, glandulon A, B, and C—bizabolene-type sesquiterpenes found in *H. annuus*—also exhibited antimicrobial effects, particularly against *B. brevis* [22].

In summary, these observations point out STLs as key contributors to the diverse pharmacological profile of the *Helianthus* genus and reinforce their potential as leads for anticancer, antiparasitic, and antimicrobial drug development.

Regarding phenolic compounds, caffeoylquinic acids—such as cynarin, chlorogenic acid, 3-O-caffeoylquinic acid, and 1,5-dicaffeoylquinic acid—isolated from *H. annuus* and *H. tuberosus*, have been shown to possess antioxidant and antidiabetic properties [62,75,91,100,117]. In *H. annuus*, these compounds have also been associated with a reduction in body weight among obese women [87]. Additionally, the choleretic and biliary-stimulating effects observed in dry extracts of *H. annuus* are likely attributed to its flavonoid content [112]. Taken together, these findings highlight phenolic compounds as key contributors to the metabolic, hepatobiliary, and antioxidant activities of *Helianthus* species, reinforcing their relevance for the development of functional foods and phytotherapeutic agents.

In addition to the major phytochemical classes traditionally associated with *Helianthus*, evidence indicates that this genus produces other secondary metabolites, including alkaloids, phytosterols, tocopherols, polysaccharides, and bioactive proteins, which contribute to a broad spectrum of biological activities. Although many of these compounds have been mainly investigated in *H. annuus* and *H. tuberosus*, their reported antioxidant, metabolic, neuroprotective, and enzyme-targeting effects highlight the need to further explore their distribution, mechanisms of action, and pharmacological relevance across the genus.

## 10. Conclusions

This review compiles and analyzes the scientific literature on species within the *Helianthus* genus, highlighting that, despite the genus’s diversity, *H. annuus* and *H. tuberosus* are the most widely used in traditional medicine, typically prepared as infusions, decoctions, or macerations. Phytochemical studies indicate that STLs and phenolic compounds are the most relevant chemical classes across the genus. These species exhibit a broad spectrum of in vitro and in vivo biological activities, including antidiabetic, antimicrobial, anti-inflammatory, antioxidant, antiparasitic, and anticancer effects.

While *H. annuus* and *H. tuberosus* have been extensively studied, with numerous reports on terpenoids, flavonoids, phenolic acids, and diverse pharmacological activities, other *Helianthus* species remain largely unexplored. Comparative analyses show that *H. annuus* displays the highest chemical diversity and pharmacological potential, including anticancer, antidiabetic, antioxidant, and antimicrobial activities. In contrast, *H. tuberosus* is mainly valued for its glycosylated flavonols, phenolic acids, and functional food properties. Less-studied species, such as *H. angustifolius*, *H. debilis*, and *H. salicifolius*, exhibit only sporadic bioactivity reports, representing significant research gaps.

Systematic investigations of underexplored species could uncover novel bioactive compounds. Targeted studies on pharmacologically critical compound classes, including STLs, diterpenes, triterpenes, and phenolic acids, may help validate traditional uses and support translational applications. Furthermore, integrating preclinical and clinical research could facilitate the development of *Helianthus*-derived functional foods, nutraceuticals, or therapeutic agents, expanding the genus’ potential for human health applications.

Overall, this review provides an updated synthesis of ethnobotanical, phytochemical, and pharmacological data, emphasizing chemical composition and bioactivity of extracts and isolated compounds, while identifying underexplored species and highlighting the therapeutic potential of plants of this genus.

## Figures and Tables

**Table 1:** List of *Helianthus* species and geographical distribution.

Accepted Name Species	Geographical Distribution
*Helianthus agrestis* Pollard	Florida, Georgia
*Helianthus* × *alexidis* B. Boivin (*H. maximilianii* × *H. nuttallii*)	Central Canada (Manitoba)
*Helianthus* × *ambiguus* (Torr. & A. Gray) Britton (*H. annuus* × *H. petiolaris*)	North Central to Northeastern U.S.A. (Michigan, Ohio, New York, Wisconsin)
*Helianthus angustifolius* L.	North Central and Eastern U.S.A.
*Helianthus annuus* L.	Southwestern U.S.A to Mexico
*Helianthus anomalus* S. F. Blake	Arizona, Utah
*Helianthus argophyllus* Torr. & A. Gray	Southeastern U.S.A (Florida, North Carolina, Texas)
*Helianthus arizonensis* R. C. Jacks	Arizona, New Mexico
*Helianthus atrorubens* L.	New Jersey to Southeastern U.S.A.
*Helianthus bolanderi* A. Gray	Western U.S.A (California, Oregon)
*Helianthus* × *brevifolius* E. Watson (*H. grosseserratus* × *H. mollis*)	North Central and Northeastern U.S.A. (Illinois, Ohio)
*Helianthus californicus* DC.	California and Mexico Northwest
*Helianthus carnosus* Small	Florida
*Helianthus ciliaris* DC.	Western Central U.S.A. to North Mexico
*Helianthus* × *cinereus* Torr. & A. Gray (*H. mollis* × *H. occidentalis*)	Central U.S.A. (Illinois, Texas)
*Helianthus cusickii* A. Gray	Western U.S.A. (California, Idaho, Nevada, Oregon, Washington)
*Helianthus debilis* Nutt.	Eastern U.S.A.
*Helianthus decapetalus* L.	Eastern Canada to North Central and Eastern U.S.A.
*Helianthus deserticola* Heiser	Arizona, Nevada, Utah
*Helianthus devernii* T. M. Draper	Nevada
*Helianthus dissectifolius* R.C. Jacks.	Mexico Northeast
*Helianthus divaricatus* L.	Southeastern Canada to North Central and Eastern U.S.A.
*Helianthus* × *divariserratus* R. W. Long (*H. divaricatus* × *H. grosseserratus*)	Northeastern U.S.A (Connecticut, Indiana, Michigan)
*Helianthus* × *doronicoides* Lam. (*H. giganteus* × *H. mollis*)	Central and Eastern U.S.A.
*Helianthus eggertii* Small	Southeastern U.S.A (Alabama, Kentucky, Tennessee)
*Helianthus exilis* A. Gray	California
*Helianthus floridanus* A. Gray ex Chapm.	Southeastern U.S.A.
*Helianthus giganteus* L.	Eastern Canada to North Central and Eastern U.S.A.
*Helianthus glaucophyllus* D. M. Sm.	Southeastern U.S.A (North Carolina, South Carolina, Tennessee)
*Helianthus* × *glaucus* Small (*H. divaricatus* × *H. microcephalus*)	Eastern USA
*Helianthus gracilentus* A.Gray	California, Mexico Northwest
*Helianthus grosseserratus* M. Martens	Southeastern Canada to Central and Eastern U.S.A.
*Helianthus heterophyllus* Nutt.	Southeastern U.S.A. to Texas
*Helianthus hirsutus* Raf.	Southeastern Canada and Eastern U.S.A to Mexico
*Helianthus inexpectatus* D. J. Keil & Elvin	California (Los Angeles County)
*Helianthus* × *intermedius* R. W. Long (*H. grosseserratus* × *H. maximiliani*)	Northeastern and Central U.S.A.
*Helianthus* × *kellermanii* Britton (*H. grosseserratus* × *H. salicifolius*)	North Central and Northeastern U.S.A.
*Helianthus laciniatus* A. Gray	South Central U.S.A. to Mexico
*Helianthus* × *laetiflorus* Pers. (*H. pauciflorus* × *H. tuberosus*)	Eastern Canada to Central and Eastern U.S.A.
*Helianthus laevigatus* Torr. & A. Gray	North Carolina, South Carolina, Virginia, West Virginia
*Helianthus longifolius* Pursh	Southeastern U.S.A. (Alabama, Georgia, North Carolina)
*Helianthus* × *luxurians* E. Watson (*H. giganteus* × *H. grosseserratus*)	North Central & Northeastern U.S.A. (Illinois, Minnesota, Ohio, Wisconsin)
*Helianthus maximiliani* Schrad.	Canada and USA to Northeastern Mexico.
*Helianthus microcephalus* Torr. & A. Gray	Eastern Central and Southeastern U.S.A.
*Helianthus mollis* Buc’hoz	Southeastern Canada to Central and Eastern U.S.A.
*Helianthus neglectus* Heiser	South Central USA (New Mexico, Texas)
*Helianthus niveus* (Benth.) Brandegee	Arizona, California, to North Mexico
*Helianthus nuttallii* Torr. & A. Gray	Western and Central Canada to Western and Central U.S.A.
*Helianthus occidentalis* Riddell	North Central & Eastern U.S.A.
*Helianthus paradoxus* Heiser	Texas to Northeastern Mexico
*Helianthus pauciflorus* Nutt.	Canada to North and Eastern Central U.S.A.
*Helianthus petiolaris* Nutt.	Canada and U.S.A to North Mexico
*Helianthus porteri* (A. Gray) Pruski	Southeastern U.S.A.
*Helianthus praecox* Engelm. & A. Gray	Texas
*Helianthus pumilus* Nutt.	Colorado, Wyoming
*Helianthus radula* (Pursh) Torr. & A. Gray	Southeastern U.S.A.
*Helianthus resinosus* Small	Southeastern U.S.A.
*Helianthus salicifolius* A. Dietr.	Central U.S.A.
*Helianthus schweinitzii* Torr. & A. Gray	North Carolina, South Carolina
*Helianthus silphioides* Nutt.	N. Central and SE. U.S.A.
*Helianthus simulans* E. Watson	Southeastern U.S.A to Texas
*Helianthus smithiorum* Heiser	Southeastern USA (Alabama, Georgia, Tennessee)
*Helianthus strumosus* L.	Southeastern Canada to Central and E. U.S.A.
*Helianthus tuberosus* L.	Central and Eastern Canada to U.S.A.
*Helianthus verticillatus* Small	Southeastern USA (Alabama, Georgia, Mississippi, Tennessee)
*Helianthus winteri* J. C. Stebbins	California

The taxonomic classification and geographical distribution of *Helianthus* species were obtained from “Plants of the World Online” (https://powo.science.kew.org/ accessed on 20 May 2025) and “International Plant Names Index” (https://ipni.org accessed on 20 May 2025).

**Table 2:** Terpene compounds identified or isolated from *Helianthus* species.

Name	Structure	Type of Terpenoid	Species	References
2-Oxo-8(2-methyl-2,3-epoxybutanoyl)-guaia-1(10),3,11(13)-trien-6,12-olide	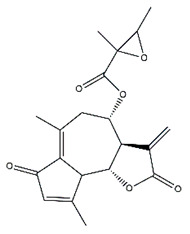	Sesquiterpene lactone	*H. microcephalus*	[59]
2-Oxo-8(2-methyl-2,3-epoxybutanoyl)-guaia-3,11(13)-dien-10-hydroxy-6,12-olide	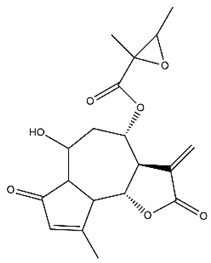	Sesquiterpene lactone	*H. microcephalus*	[59]
3-Hydroxy-8β-tigloyloxy-1,10-dehydroariglovin	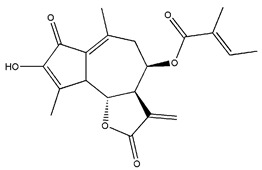	Sesquiterpene lactone	*H. tuberosus*	[62]
Ciliaric acid	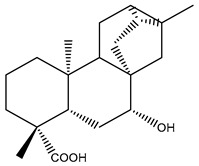	Diterpene	*H. petiolaris*; *H. ciliaris*	[22]
Annuolide A	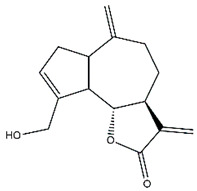	Sesquiterpene lactone	*H. annuus*	[66,67,68,69,70]
Annuolide B	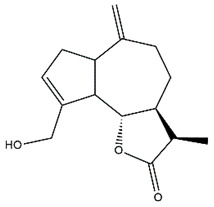	Sesquiterpene lactone	*H. annuus*	[66,67,68,69,70]
Annuolide C	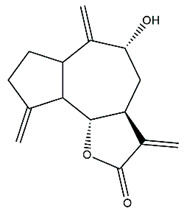	Sesquiterpene lactone	*H. annuus*	[66,67,68,69,70]
Annuolide D	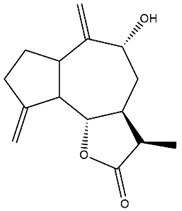	Sesquiterpene lactone	*H. annuus*	[66,67,68,69,70]
Annuolide E	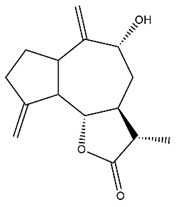	Sesquiterpene lactone	*H. annuus*	[66,67,68,69,70]
Annuolide F	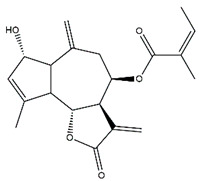	Sesquiterpene lactone	*H. annuus*	[66,67,68,69,70]
Annuolide G	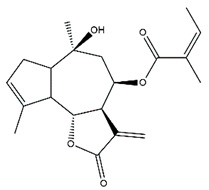	Sesquiterpene lactone	*H. annuus*	[66,67,68,69,70]
Annuolide H	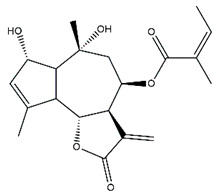	Sesquiterpene lactone	*H. annuus*	[66,67,68,69,70]
Argophyllin A	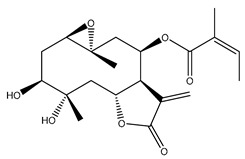	Sesquiterpene lactone	*H. argophyllus*; *H. annuus*	[21]
Argophyllin B	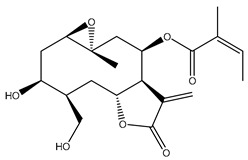	Sesquiterpene lactone	*H. argophyllus*; *H. annuus*; *H. bolanderi*	[21]
Argophyllin C	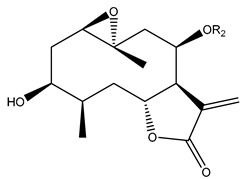	Sesquiterpene lactone	*H. argophyllus*	[21]
Argophyllone B	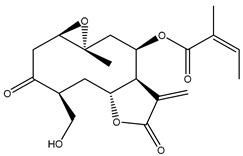	Sesquiterpene lactone	*H. annuus*, *H. argophyllus*	[72,77]
Atripliciolide angelate	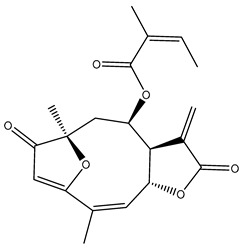	Sesquiterpene lactone	*H. debilis*; *H. petiolaris*; *H. praecox*; *H. arizonensis*; *H. decapetalus*; *H. schweinitzii*; *H. heterophyllus*	[21,58,59]
Atripliciolide isobutyrate	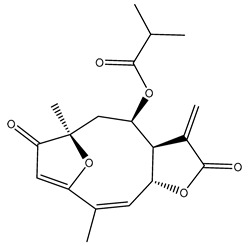	Sesquiterpene lactone	*H. angustifolius*	[59]
Atripliciolide 2-methylbutyrate	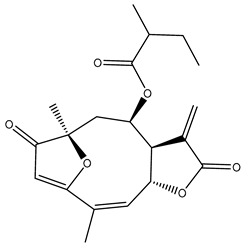	Sesquiterpene lactone	*H. heterophyllus*	[59]
Atripliciolide tiglate	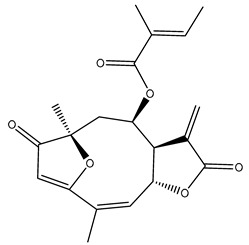	Sesquiterpene lactone	*H. arizonensis*	[58]
Atripliciolide tiglate 2-hydro-3-hydroxy	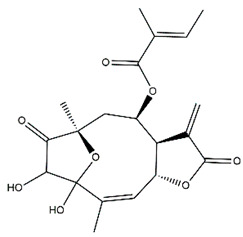	Sesquiterpene lactone	*H. tuberosus*	[59]
4,5-iso-atripliciolide angelate	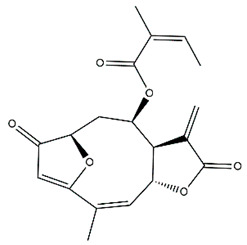	Sesquiterpene lactone	*H. angustifolius*; *H. floridanus*	[59]
4,15-iso-atripliciolide angelate	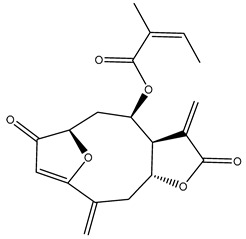	Sesquiterpene lactone	*H. tuberosus*	[62,78]
4,15-iso-atripliciolide isobutyrate	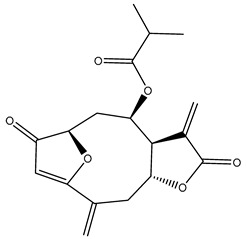	Sesquiterpene lactone	*H. angustifolius*; *H. tuberosus*	[59,61,62,78]
4,15-iso-atripliciolide-8-Isovaleryl	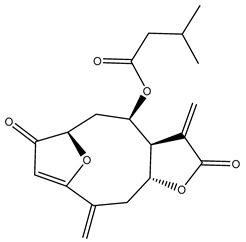	Sesquiterpene lactone	*H. angustifolius*	[61]
4,15-iso-atripliciolide methacrylate	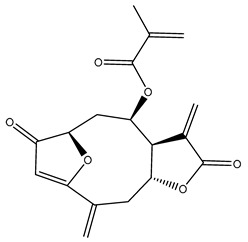	Sesquiterpene lactone	*H. tuberosus*, *H. angustifolius*, *H. tuberosus*	[61,62,78]
4,15-iso-atripliciolide-8-methylbutyryl	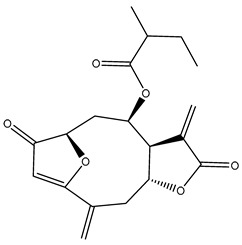	Sesquiterpene lactone	*H. angustifolius*; *H. tuberosus*	[61,78]
4,5-iso-atripliciolide tiglate	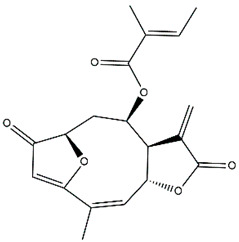	Sesquiterpene lactone	*H. schweinitzii*; *H. angustifolius*; *H. floridanus*	[59]
4,15-iso-atripliciolide tiglate	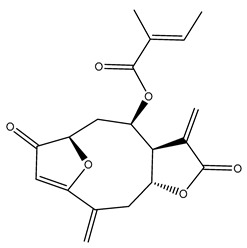	Sesquiterpene lactone	*H. tuberosus*	[62,63,78]
Budlein A	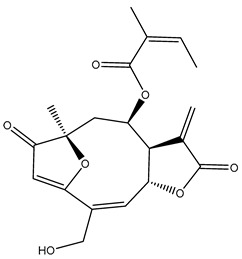	Sesquiterpene lactone	*H. debilis* ssp. *cucumerifolius*; *H. debilis*; *H. petiolaris*; *H. praecox*; *H. decapetalus*; *H. hirsutus*; *H. maximiliani*; *H. nuttallii*; *H. schweinitzii*; *H. strumosus*; *H. tuberosus*; *H. heterophyllus; H. floridanus*; *H. angustifolius; H. Iongifolius*; *H. simulans*	[21,59,78]
Budlein A isobutyrate	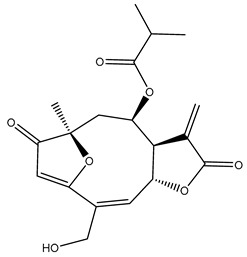	Sesquiterpene lactone	*H. tuberosus*; *H. decapetalus*	[59,78]
Budlein A isovalerate	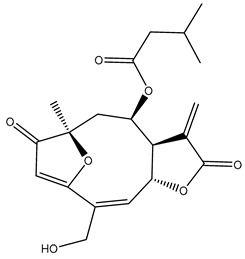	Sesquiterpene lactone	*H. maximiliani*	[59]
Budlein A methacrylate	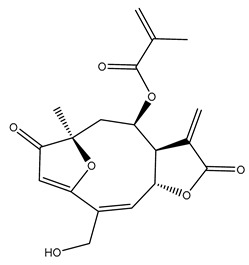	Sesquiterpene lactone	*H. tuberosus*	[62]
Budlein A 2-methylbutyrate	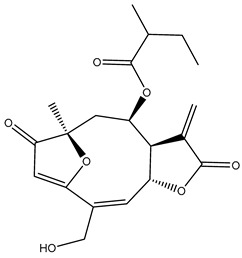	Sesquiterpene lactone	*H. debilis* ssp. *cucumerifolius*; *H. debilis*; *H. petiolaris*; *H. praecox* ssp. *hirtus*; *H. praecox* ssp. *praecox*; *H. maximiliani*; *H. strumosus*; *H. floridanus*; *H. longifolius*; *H. radula*; *H tuberosus*	[21,59,78]
Budlein A tiglate	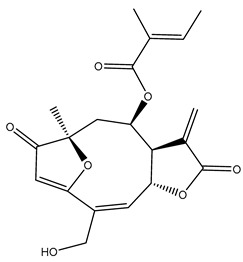	Sesquiterpene lactone	*H. tuberosus*; *H. schweinitzii*	[59,62,78]
4,15-iso-Budlein A isobutyrate	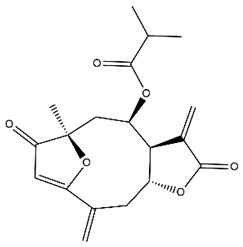	Sesquiterpene lactone	*H. simulans*; *H. floridanus*; *H. californicus*; *H. angustifolius*; *H. tuberosus*	[59,78]
Chamissonin 3-acetyl	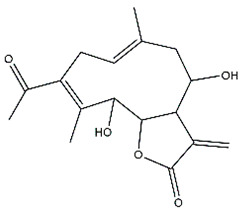	Sesquiterpene lactone	*H. niveu* ssp. *canescens*	[21]
Chamissonin 3-acetyl-11,13-dihydro	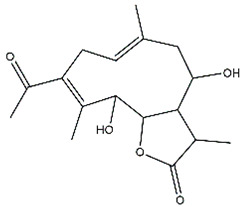	Sesquiterpene lactone	*H. californicus*	[59]
Chamissonin 11,13-dihydro	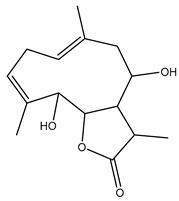	Sesquiterpene lactone	*H. pumilus*	[58]
Ciliarin	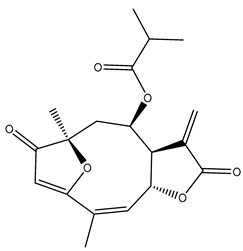	Sesquiterpene lactone	*H. arizonensis*; *H. ciliaris*; *H. angustifolius*	[58]
Costunolide 8 β-14-dihydroxy	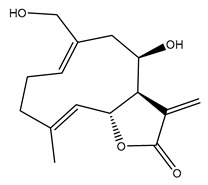	Sesquiterpene lactone	*H. niveu* ssp. *niveus*; *H. californicus*; *H. grosseserratus; H. strumosus*; *H. tuberosus*	[21,59,78]
Costunolide 8-14-dihydroxy-14-isobutyrate	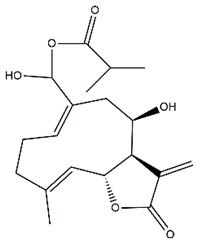	Sesquiterpene lactone	*H. niveu* ssp. *niveus*; *H. pauciflorus*	[21,59]
Desacetylovatifolin	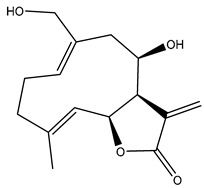	Sesquiterpene lactone	*H. tuberosus*	[62]
*Ent*-12- β acetoxykaurenoic acid	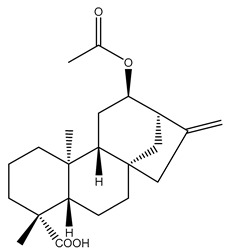	Diterpene	*H. decapetalus*; *H. decapetalus* var. *multiflorus*; *H. hirsutus*; *H. rigidus*	[22]
*Ent*-13(S)-angeloxyatisenoic acid	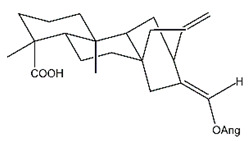	Diterpene	*H. decapetalus*	[22]
*Ent*-12,16-cyclokaurenoic acid (trachylobanoic acid)	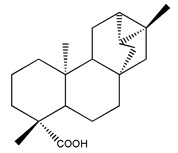	Diterpene	*H. debilis*; *H. giganteus*; *H. hirsutus*; *H. rigidus*; *H. tomentosus*	[22,56]
*Ent*-9,11-didehydrokaurenoic acid	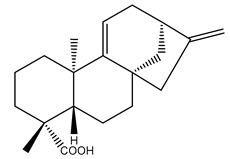	Diterpene	*H. grosse-serratus*; *H. maximiliani*	[22]
*Ent*-kaurenoic acid	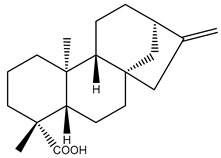	Diterpene	*H. decapetalus*; *H. giganteus*; *H. nuttallii*; *H. rigidus*	[22]
Erioflorin	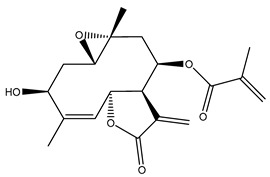	Sesquiterpene lactone	*H. tuberosus*	[78]
Eupasserin	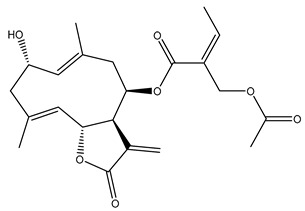	Sesquiterpene lactone	*H. pumilus*; *H. gracilentus*; *H. ciliaris*; *H. mollis*; *H. porteri*; *H. occidentalis; H. pauciflorus*	[58,59]
Eupasserin acrylate 2-hydroxyethyl	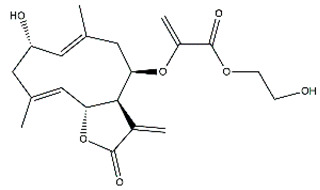	Sesquiterpene lactone	*H. pumilus*; *H. gracilentus*; *H. cusickii*; *H. pumilus*; *H. mollis*; *H. occidentalis*; *H. resinosus*	[58,59]
Eupasserin angelate	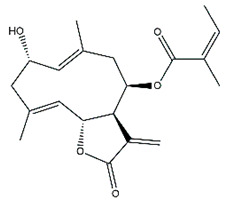	Sesquiterpene lactone	*H. gracilentus*; *H. pumilus*	[58]
Eupasserin desacetyl	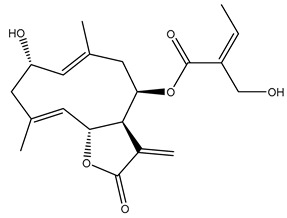	Sesquiterpene lactone	*H. porteri*; *H. gracilentus*; *H. pumilus*; *H. decapetalus*; *H. mollis*; *H. glaucophyllus; H. porteri*; *H. occidentalis*; *H. californicus*; *H. divaricatus*; *H. eggertii*; *H. maximiliani*; *H. resinosus*; *H. salicifolius*; *H. schweinitzii*; *H. laevigatus*; *H tuberosus*; *H. arizonensis*;	[21,58,59,78]
Eupasserin dessarracenate	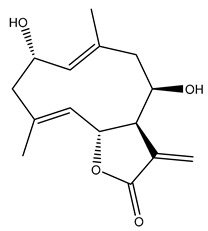	Sesquiterpene lactone	*H. pumilus*; *H. nuttallii*; *H. resinosus*	[58,59]
Eupasserin isovalerate	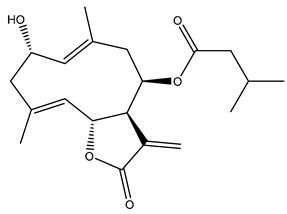	Sesquiterpene lactone	*H. gracilentus*	[58]
Eupatolide	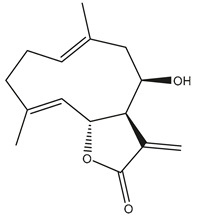	Sesquiterpene lactone	*H. argophyllus*	[21]
Glandulone A	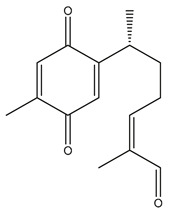	Sesquiterpene	*H. annuus*	[21,58,59]
Glandulone B	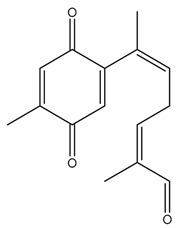	Sesquiterpene	*H. annuus*	[21,58,59]
Glandulone C	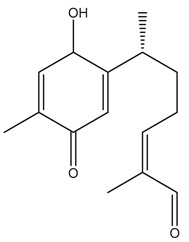	Sesquiterpene	*H. annuus*	[21,58,59]
Haageanolide	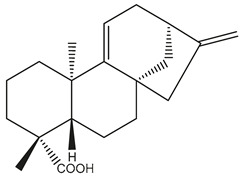	Sesquiterpene lactone	*H. annuus*	[72]
Heliangin	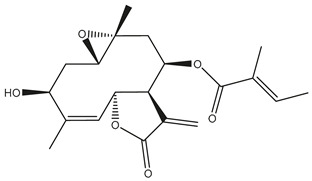	Sesquiterpene lactone	*H. tuberosus*	[65,78]
Helianthoside 1	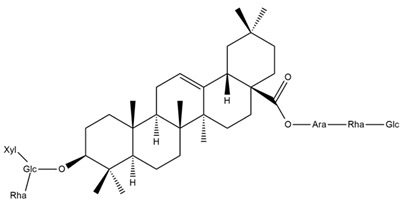	Triterpenoid glycoside	*H. annuus*	[56,75,76]
Helianthoside 2	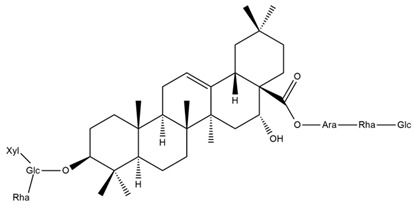	Triterpenoid glycoside	*H. annuus*	[56,75,76]
Helianthoside 3	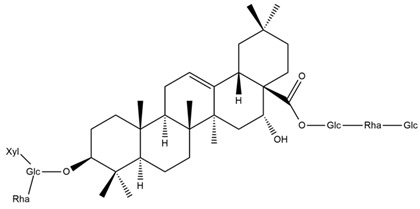	Triterpenoid glycoside	*H. annuus*	[56,75,76]
Helianthoside 4	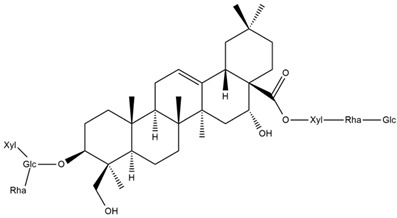	Triterpenoid glycoside	*H. annuus*	[56,75,76]
Helianthoside 5	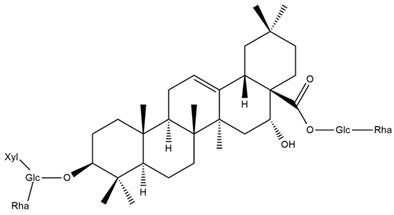	Triterpenoid glycoside	*H. annuus*	[56,75,76]
Helianthoside B	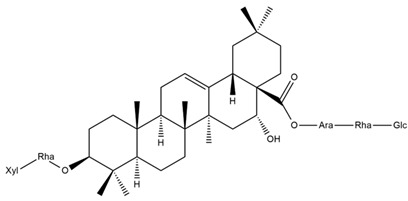	Triterpenoid glycoside	*H. annuus*	[56,75,76]
Heliantuberolide-8-O-tiglate	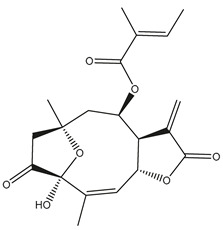	Sesquiterpene lactone	*H. tuberosus*	[64]
Helieudesmanolide A	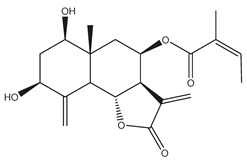	Sesquiterpene lactone	*H. annuus*	[66,67,68,69,70]
Helieudesmanolide B	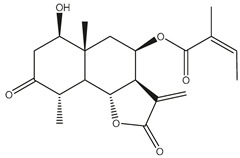	Sesquiterpene lactone	*H. annuus*	[71]
Helikaurolide A	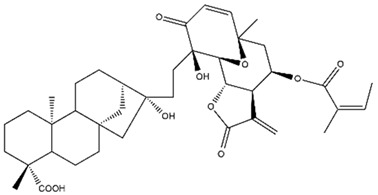	Diterpene	*H. annuus*	[74]
Helikaurolide B	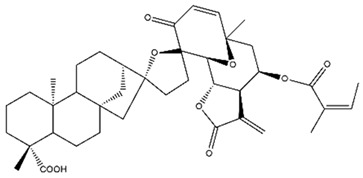	Diterpene	*H. annuus*	[74]
Helikaurolide C	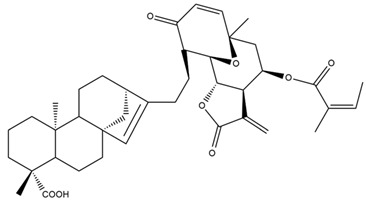	Diterpene	*H. annuus*	[74]
Helikaurolide D	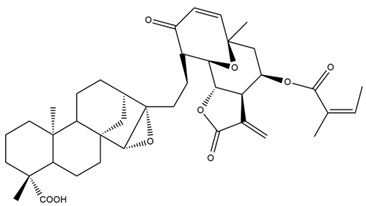	Diterpene	*H. annuus*	[74]
Helivypolide B	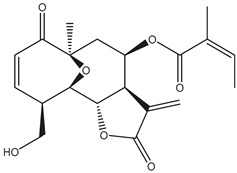	Sesquiterpene lactone	*H. annuus*	[66,67,68,69,70]
Helivypolide D	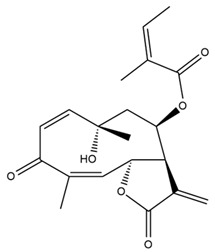	Sesquiterpene lactone	*H. annuus*	[56,66,67,68,69,70]
Helivypolide E	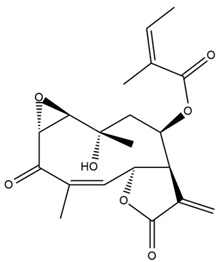	Sesquiterpene lactone	*H. annuus*	[56,66,67,68,69,70]
Helivypolide F	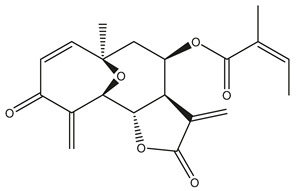	Sesquiterpene lactone	*H. annuus*	[56,66,67,68,69,70]
Helivypolide G	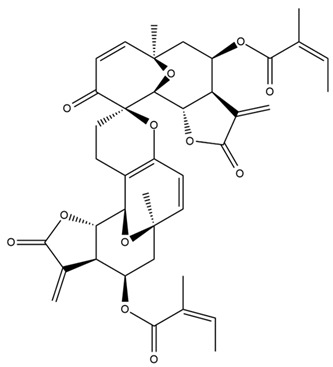	Sesquiterpene lactone	*H. annuus*	[56]
Helivypolide H	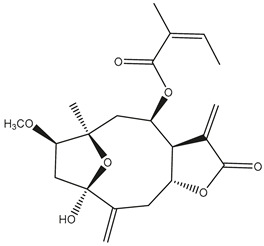	Sesquiterpene lactone	*H. annuus*	[56,66,67,68,69,70]
Helivypolide I	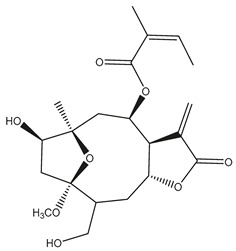	Sesquiterpene lactone	*H. annuus*	[56,66,67,68,69,70]
Helivypolide J	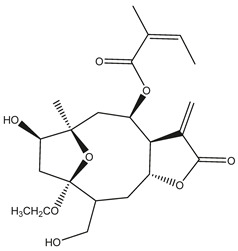	Sesquiterpene lactone	*H. annuus*	[56,66,67,68,69,70]
Helivypolide K	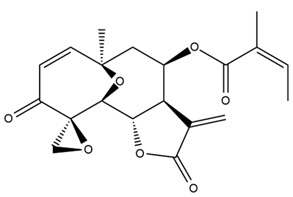	Sesquiterpene lactone	*H. annuus*	[56,71]
Helivypolide L	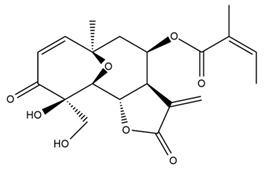	Sesquiterpene lactone	*H. annuus*	[56,71]
Leptocarpin	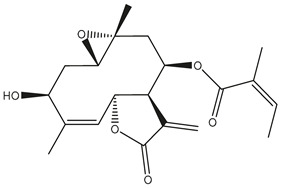	Sesquiterpene lactone	*H. tuberosus*; *H. simulans*	[59,78]
Melampolide	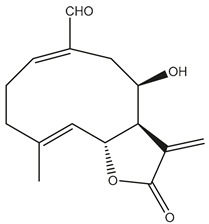	Sesquiterpene lactone	*H. tuberosus*	[62,63]
Mollisorin B	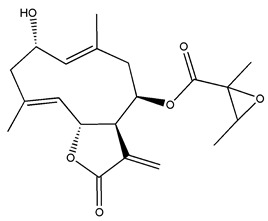	Sesquiterpene lactone	*H. porteri*; *H. agrestis*; *H. gracilentus*; *H. pumilus*; *H. tuberosus*	[21,58,78]
Mollisorin B 2′R,3′R	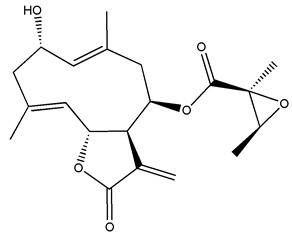	Sesquiterpene lactone	*H. pumilus*; *H. gracilentus*; *H. arizonensis*; *H. laciniatus*; *H. laevigatus*; *H. giganteus*; *H. smithii*; *H. californicus; H. resinosus*; *H. atrorubens*; *H. microcephalus*	[58,59]
Mollisorin B 2′S,3′S	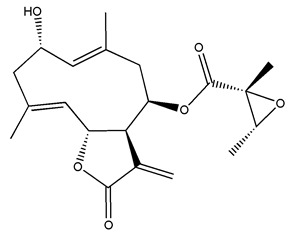	Sesquiterpene lactone	*H. cusickii*; *H. pumilus*; *H. gracilentus*; *H. arizonensis*; *H. laciniatus*; *H. laevigatus*; *H. giganteus*; *H. smithii*; *H. divaricatus*; *H. maximiliani*; *H. simulans*; *H. eggertii*; *H. grosseserratus; H. nuttallii*; *H. glaucophyllus; H. porter*; *H. silphioides*; *H. occidentalis*; *H. pauciflorus*; *H.* *angustifolius*; *H. floridanus*	[58,59]
Niveusin A	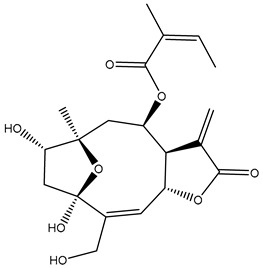	Sesquiterpene lactone	*H. niveu* ssp. *canescens*; *H. niveus* ssp. *tephrodes*; *H. argophyllus*; *H. annuus*; *H. deserticola*; *H. neglectus*; *H. gracilentus*; *H. grosseserratus*; *H. salicifolius*; *H. laevigatus*; *H. smithii*; *H. pauciflorus*	[21,58,59]
Niveusin A 1,2-anhydrido	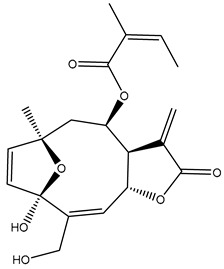	Sesquiterpene lactone	*H. annuus*	[21]
Niveusin A 1,2-anhydrido-4,5-dihydro	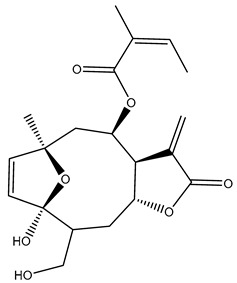	Sesquiterpene lactone	*H. annuus*	[21]
Niveusin A 4,5-dihydro	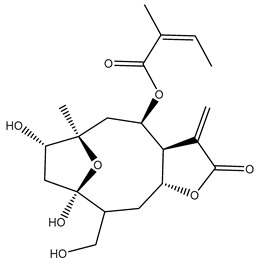	Sesquiterpene lactone	*H. annuus*	[21]
Niveusin A 1-methoxy-4,5-di-hydro	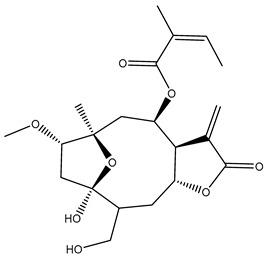	Sesquiterpene lactone	*H. annuus*; *H. argophyllus*	[21]
Niveusin A 2-methylbutyrate	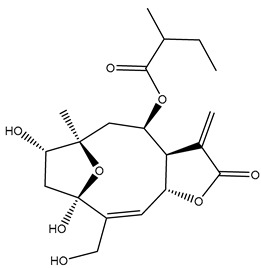	Sesquiterpene lactone	*H. heterophyllus*	[59]
Niveusin B	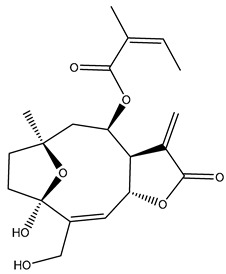	Sesquiterpene lactone	*H. niveu* ssp. *canescens*; *H. annuus*; *H. anomalus*; *H. argophyllus*; *H. bolanderi*; *H. niveus*; *H. praecox* ssp *o runyonii*; *H. praecox* ssp. *praecox*; *H. decapetalus*; *H. smithii*	[21,59]
Niveusin B isobutyrate	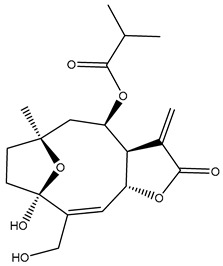	Sesquiterpene lactone	*H. gracilentus*; *H. laciniatus*	[58]
Niveusin B 2-methylbutyrate	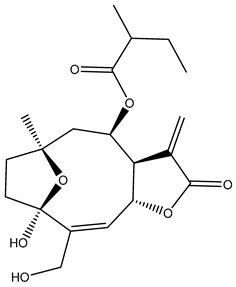	Sesquiterpene lactone	*H. laciniatus*	[58]
Niveusin C	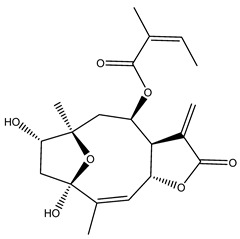	Sesquiterpene lactone	*H. niveu* ssp. *canescens*; *H. annuus*; *H. bolanderi*; *H. neglectus*; *H. niveus* ssp. *canescens*; *H. niveus* ssp. *tephrodes*; *H. praecox* ssp. *praecox*; *H. laciniatus*; *H. maximiliani*; *H. salicifolius*; *H. strumosus*; *H. laevigatus*; *H. pauciflorus*	[21,58,59]
Niveusin C des-3-hydroxy	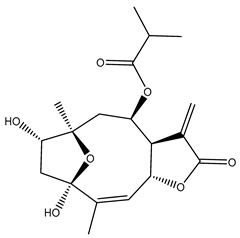	Sesquiterpene lactone	*H. maximiliani*	[59]
Orizabin	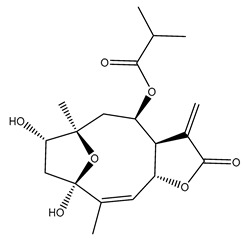	Sesquiterpene lactone	*H. niveu* ssp. *niveus*	[21]
Pinnatifidin-1α-acetoxy-	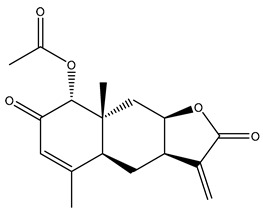	Sesquiterpene lactone	*H. tuberosus*	[62,63]
Pinnatifidin 1,2-dihydroxy	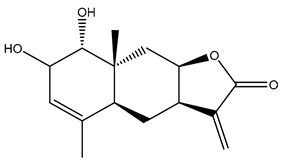	Sesquiterpene lactone	*H. pumilus*; *H. grosseserratus*; *H. maximiliani*; *H. nuttallii*; *H. carnosus*; *H. floridanus*; *H. hirsutus*; *H. resinosus*; *H. strumosus*; *H. porter*; *H. smithii*; *H. Iongifolius*; *H. tuberosus*	[58,59,78]
Pinnatifidin 1-hydroxy	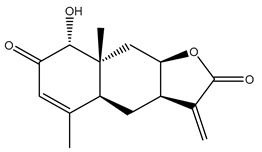	Sesquiterpene lactone	*H. grosseserratus*; *H. maximiliani*; *H. nuttallii*; *H. carnosus*; *H. floridanus*; *H. pauciflorus*	[59]
Simsiolide	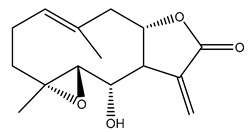	Sesquiterpene lactone	*H. argophyllus*	[21]
Tagitinin A	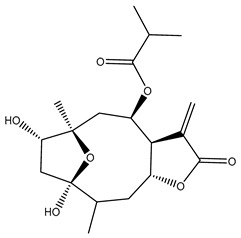	Sesquiterpene lactone	*H. niveu* ssp. *niveus*	[21]
Tagitinin C 4,5-di-hydroxy	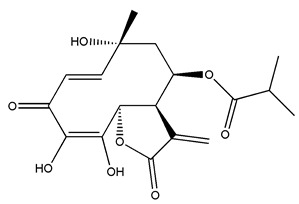	Sesquiterpene lactone	*H. niveu* ssp. *niveus*	[21]
Tagitinin E	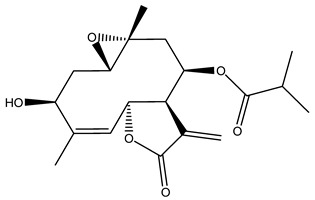	Sesquiterpene lactone	*H. tuberosus*	[78]
Ternifolin angelate	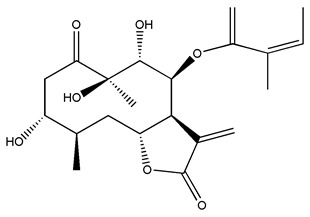	Sesquiterpene lactone	*H. californicus*; *H. schweinitzii*	[59]
Tifruticin	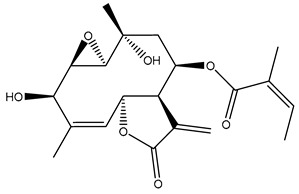	Sesquiterpene lactone	*H. cusickii*; *H. eggertii*; *H. maximiliani*; *H. floridanus*	[58,59]
Tifruticin acetyl	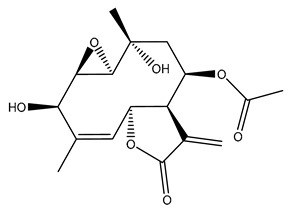	Sesquiterpene lactone	*H. pumilus*; *H. decapetalus*; *H. eggertii*; *H. maximiliani*	[58,59]
Tifruticin desoxy	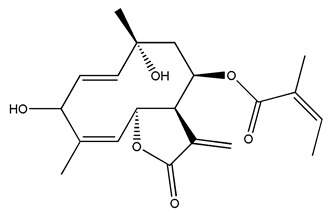	Sesquiterpene lactone	*H. cusickii*; *H. arizonensis*; *H. pauciflorus*; *H. laevigatus*	[58,59]
Tifruticin desoxy-1,2-dihydro	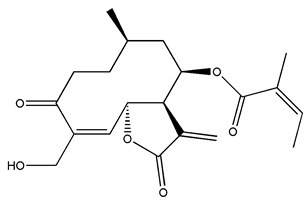	Sesquiterpene lactone	*H. arizonensis*; *H. ciliaris*; *H. pauciflorus*; *H. salicifolius*; *H. maximiliani*	[58,59]
Tifruticin desoxy-3-dehydro-15-hydroxy	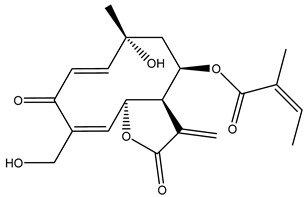	Sesquiterpene lactone	*H. annuus*; *H. anomalus*; *H. argophyllus*; *H. neglectus*; *H. gracilentus*; *H. ciliaris*	[21,56,58]
Tifruticin 1-hydroxy	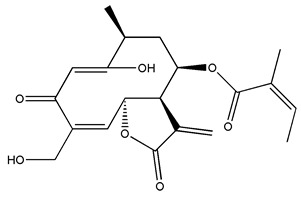	Sesquiterpene lactone	*H. maximiliani*; *H. occidentalis*	[59]
Tirotundin	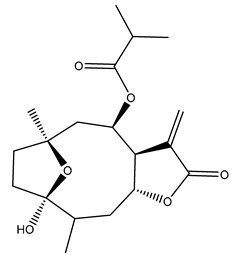	Sesquiterpene lactone	*H. niveu* ssp. *niveus*	[21]
Tithifolin epoxyangelate, 14-acetoxy	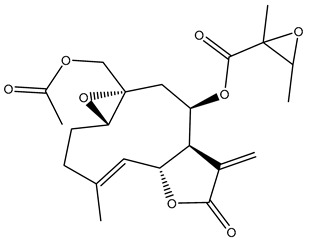	Sesquiterpene lactone	*H. tuberosus*	[78]

**Table 3:** Flavonoids and phenolic compounds identified or isolated from *Helianthus* species.

Name	Structure	Type of Phenolic Compound	Species	References
1,5-Dicaffeoylquinic acid	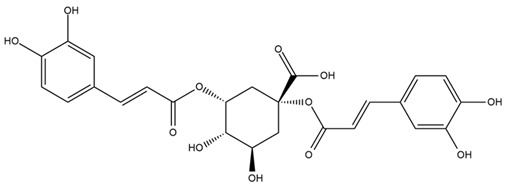	Phenolic acid	*H. tuberosus*	[81]
2′,4′,4-Trihydroxy-3′-methoxychalcone	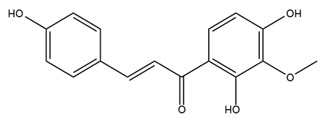	Chalcone	*H. annuus*	[79]
3,4-Di-O-caffeoylquinic acid	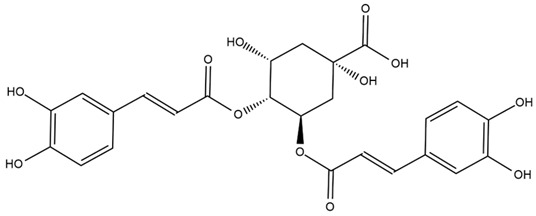	Phenolic acid	*H. tuberosus*; *H. annuus*	[81,87]
3,5-Di-O-caffeoylquinic acid	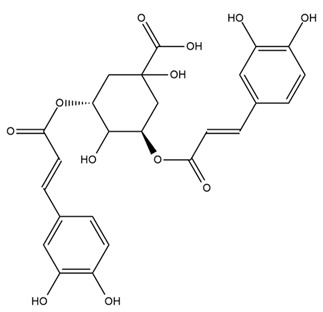	Phenolic acid	*H. annuus*	[87]
3-Feruloylquinic acid	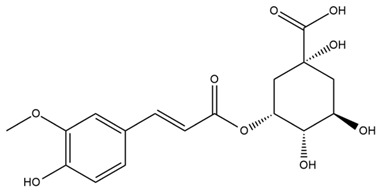	Phenolic acid	*H. tuberosus*	[81]
3-O-Caffeoylquinic acid	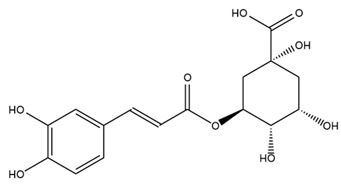	Phenolic acid	*H. annuus*	[87]
4,5-Di-O-caffeoylquinic acid	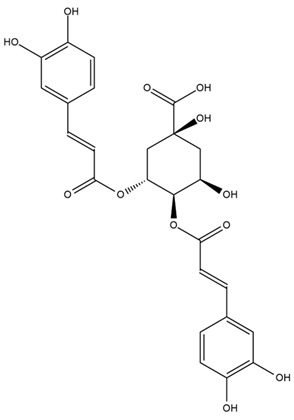	Phenolic acid	*H. annuus*	[87]
4-O-Caffeoylquinic acid	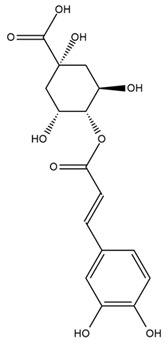	Phenolic acid	*H. annuus*	[87]
5,8-Dihydroxy-6,7,3′,4′-tetramethoxy flavone	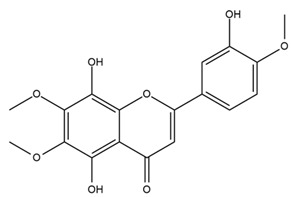	Flavone	*H. tuberosus*	[62]
5-Deoxy-flavenone	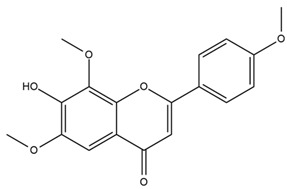	Flavone	*H. annuus*	[82]
5-Hydroxy-4,6,4′-trimethoxyaurone	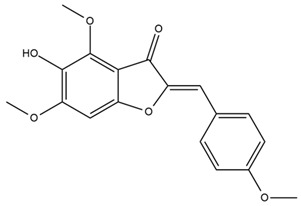	Aurone	*H. annuus*	[22,79]
5-Hydroxy-7,4′-dimethoxyflavan	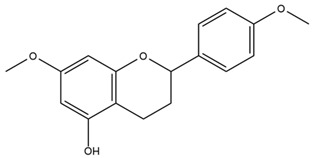	Flavane	*H. microcephalus*	[79]
5-O-Caffeoylquinic acid	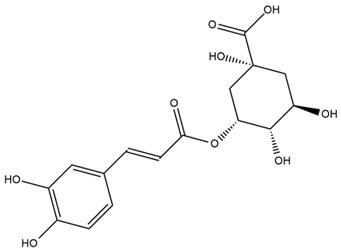	Phenolic acid	*H. annuus*	[87]
5-O-Feruloylquinic acid	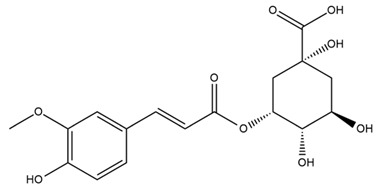	Phenolic acid	*H. annuus*	[86]
5-O-*p*-Coumaroylquinic acid	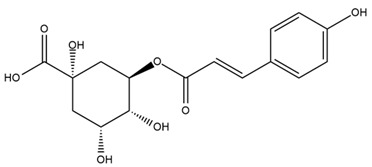	Phenolic acid	*H. annuus*	[86]
Acerosin	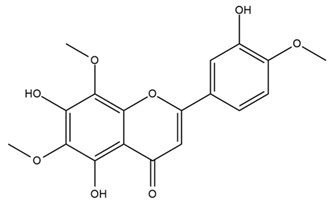	Flavone	*H. annuus*; *H. tuberosus*; *H. strumosus*; *H. simulans*; *H. microcephalus*	[76,82,83,85]
Andrographin	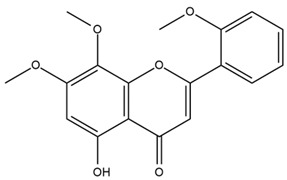	Flavone	*H. tuberosus*	[81]
Apigenin	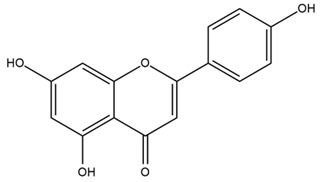	Flavone	*H. annuus*	[86]
Astragaline	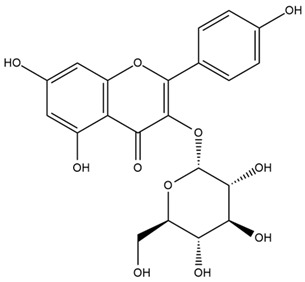	Flavonol glycoside	*H. tuberosus*	[80]
Biochanin A	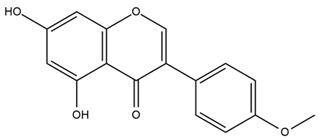	Isoflavone	*H. annuus*	[86]
Butein	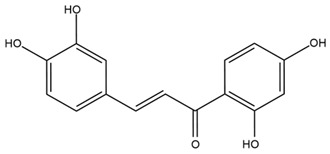	Chalcone	*H. tuberosus*	[80]
Caffeic acid	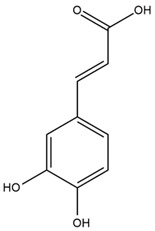	Phenolic acid	*H. annuus*	[76]
Catechin	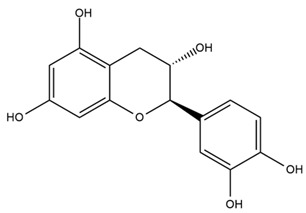	Flavanol	*H. tuberosus*	[81]
Chlorogenic acid	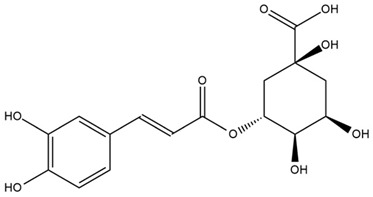	Phenolic acid	*H. tuberosus*	[81]
*cis*-Ferulic acid	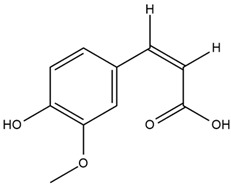	Phenolic acid	*H. annuus*	[76]
Coreopsin (Butein-4′-glucoside)	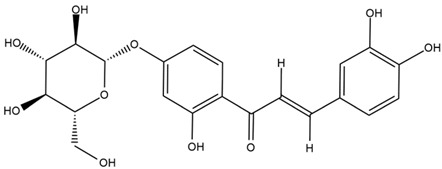	Chalcone (tetrahydroxychalcone)	*H. angustifolius*; *H. floridanus*; *H. heterophyllus*; *H. longifolius*; *H. atrorubens*; *H. glaucophyllus*; *H. gracilentus*	[22,79]
Daidzein	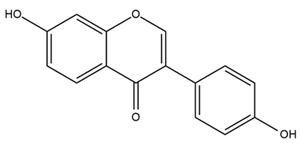	Isoflavone	*H. annuus*	[86]
Daidzin	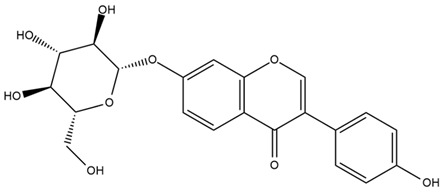	Isoflavone glycoside	*H. annuus*	[86]
Demethoxysudachitin	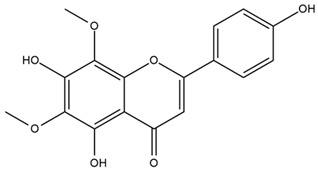	Flavone	*H. annuus*	[82,83]
Dihydroflavonol	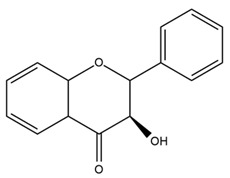	Hydroxyflavanone	*H. annuus*	[86]
Epigallocatechin gallate	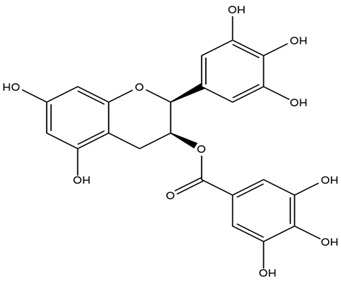	Flavonoid (flavan 3-ol)	*H. tuberosus*	[81]
Fisetin	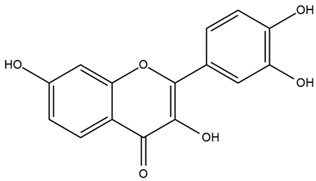	Flavonol	*H. gracilentus*	[79]
Formononetin	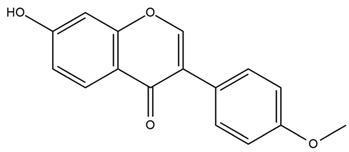	Isoflavone	*H. annuus*	[86]
Gallic acid	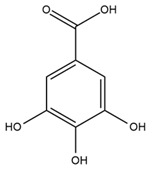	Phenolic acid	*H. annuus*	[76]
Gardenin B	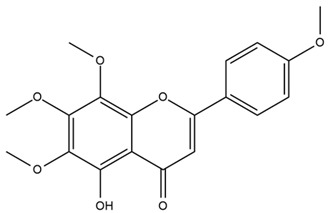	Flavone	*H. annuus*	[82]
Genistein	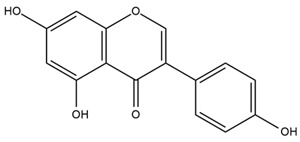	Isoflavone	*H. annuus*	[86]
Genistin	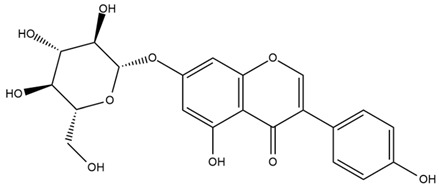	Isoflavone glycoside	*H. annuus*	[86]
Heliannone A	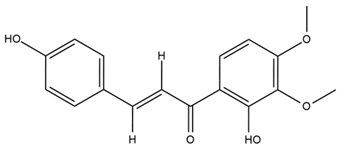	Chalcone	*H. annuus*	[79]
Heliannone B	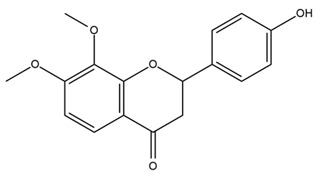	Flavanone	*H. annuus*	[76]
Heliannone C	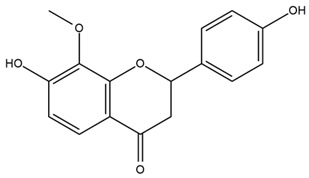	Flavanone	*H. annuus*	[79]
Hispidulin	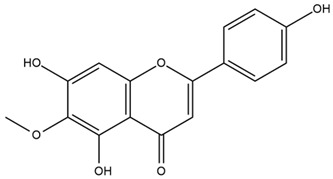	Flavone	*H. angustifolius*; *H. floridanus*; *H. microcephalus*; *H. simulans*	[22]
Hymenoxin	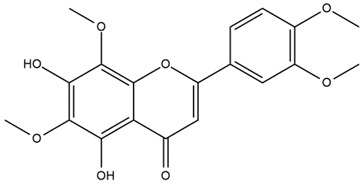	Flavone	*H. angustifolius*; *H. floridanus*; *H. microcephalus*; *H. simulans*; *H. strumosus*; *H. tuberosus*	[22,80,81]
Isoliquiritigenin	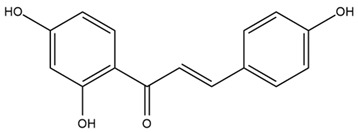	Chalcone	*H. tuberosus*; *H. longifolius*	[22,84,85]
Isoquercetin (quercetin 3-O-β-d-glucopyranoside	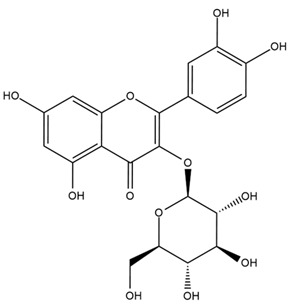	Flavonol glycoside	*H. angustifolius*; *H. carnosus*; *H. floridanus*; *H. heterophyllus*; *H. longifolius*; *H. microcephalus*; *H. tuberosus*	[22,80]
Isorhamnetin-3-O-glucoside	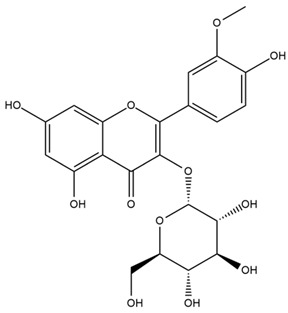	Flavonol glycoside	*H. tuberosus*	[80]
Isorhamnetin-3-O-glucuronide	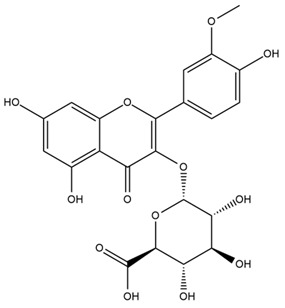	Flavonol glycoside	*H. tuberosus*	[80]
Jaceosidin	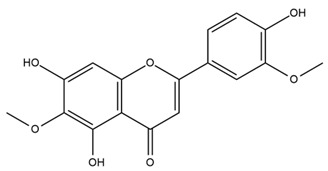	Flavone	*H. angustifolius*; *H. floridanus*; *H. microcephalus*; *H. simulans*	[22]
Kaempferol	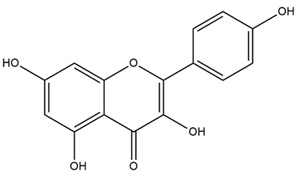	Flavonol	*H. tuberosus*; *H. annuus*	[81,86]
Kaempferol 3-glucuronide	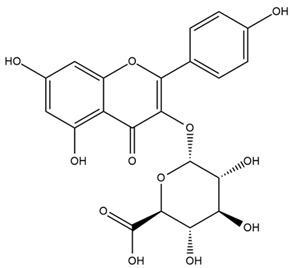	Flavonol glycoside	*H. tuberosus*	[80]
Kaempferol-3-O-glucoside	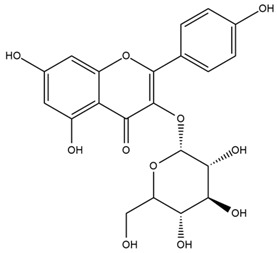	Flavonol glycoside	*H. tuberosus*	[81,84]
Kukulkanin B	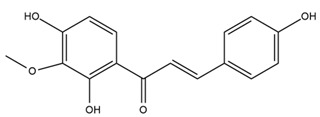	Chalcone	*H. annuus*; *H. tuberosus*	[76,80]
Liquiritigenin	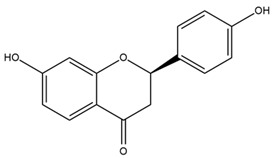	Flavanone (2,3-Dihydroflavone)	*H. tuberosus*	[80,85]
Luteolin	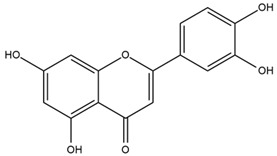	Flavone	*H. microcephalus*; *H. annuus*	[76]
Methylsudachitin (5,4′-Dihidroxy-6,7,8,3′-tetramethoxyflavone)	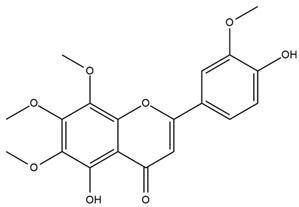	Flavone	*H. annuus*	[82]
Nepetin	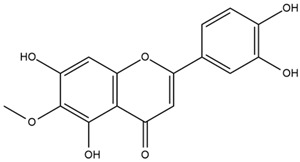	Flavone	*H. angustifolius*; *H. floridanus*; *H. microcephalus*; *H. simulans*	[22]
Nevadensin	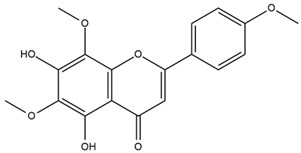	Flavone	*H. annuus*; *H. angustifolius*; *H. floridanus*; *H. microcephalus*; *H. simulans*; *H. strumosu*; *H. tuberosus*	[22,80,81,83]
Nobiletin	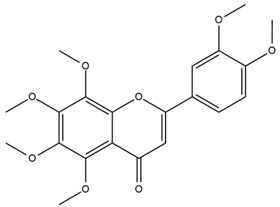	Flavone	*H. tuberosus*	[81]
p-Coumaric acid	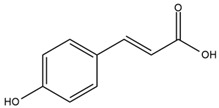	Phenolic acid	*H. annuus*	[76]
p-Coumaroylquinic acid	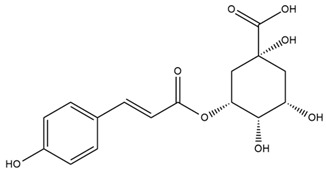	Phenolic acid	*H. tuberosus*	[81]
Pedunculin	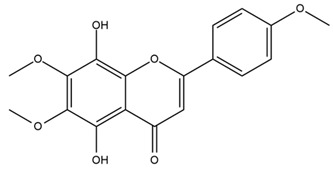	Flavone	*H. tuberosus*	[62]
Protocatechuic acid	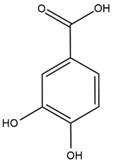	Phenolic acid	*H. annuus*	[76]
Puerarin	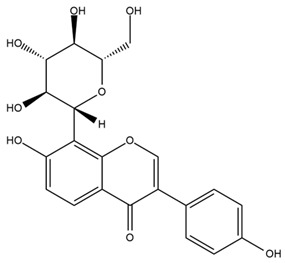	Isoflavone glycoside	*H. tuberosus*	[81]
Quercetin	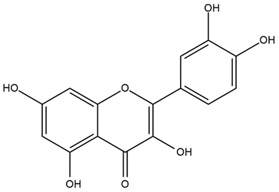	Flavonol	*H. annuus*	[86]
Quercetin 7-O-glucoside	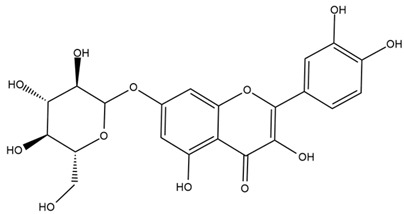	Flavonol glycoside	*H. floridanus*; *H. carnosus*; *H. microcephalus*; *H. tuberosus*	[22,84,85]
Rhamnazin	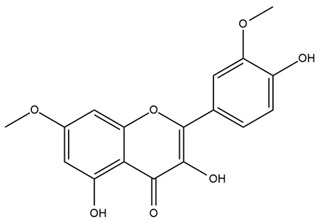	Flavonol	*H. tuberosus*	[81]
Rutin	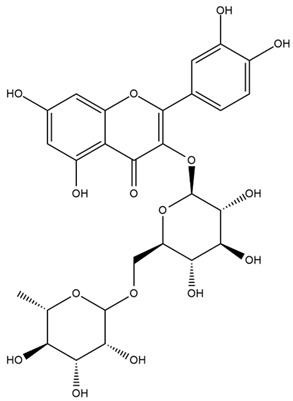	Flavonol glycoside	*H. annuus*; *H. tuberosus*	[76,81]
Salicylic acid	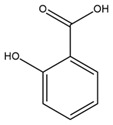	Phenolic acid	*H. tuberosus*	[81]
Sideritiflavone	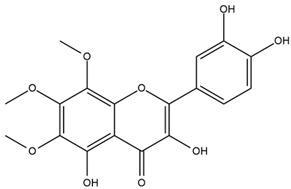	Flavone	*H. annuus*	[82]
Silymarin	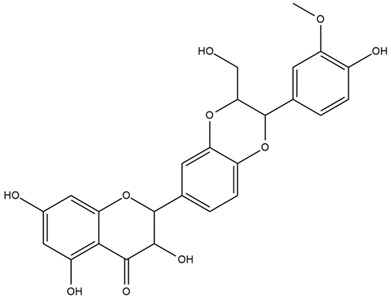	Flavonolignan	*H. tuberosus*	[81]
Sinapic acid	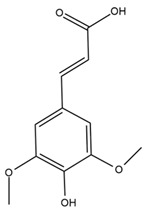	Phenolic acid	*H. annuus*	[76]
Sudachitin	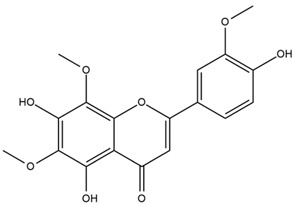	Flavone	*H. simulans*; *H. strumosus*	[82]
Sulfuretin	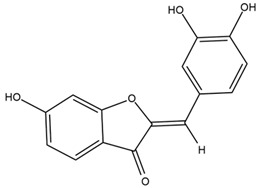	Aurone	*H. angustifolius*; *H. floridanus*; *H. heterophyllus*; *H. longifolius*; *H. atrorubens*; *H. glaucophyllus*; *H. gracilentus*; *H. tuberosus*	[22,79,85]
Sulfuretin 6-glucoside	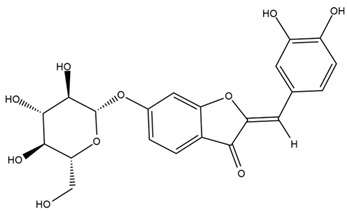	Aurone gluycoside	*H. tuberosus*	[85]
Tambulin	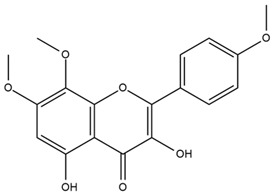	Flavonol	*H. annuus*	[79]
*trans*-Ferulic acid	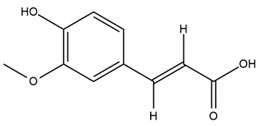	Phenolic acid	*H. annuus*	[76]
Xanthomicrol	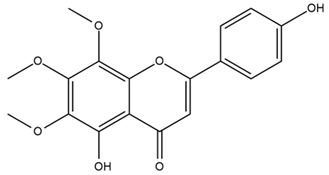	Flavone	*H. annuus*	[83]

**Table 4:** Occurrence and identification confidence of the most representative flavonoids in *Heliantus* species.

Species	Compound									
	Nevadensin	Hymenoxin	Hispidulin	Nepetin	Jaceosidin	Coreopsin	Sulfuretin	Acerosin	Quercetin 7-O-glucoside	Isoquercetin
*H. annuus*	SC	-	-	-	-	-	-	SC	-	-
*H. angustifolius*	L	L	L	L	L	L	L	-	-	L
*H. floridanus*	L	L	L	L	L	L	L	-	L	L
*H. microcephalus*	L	L	L	L	L	-	-	L	L	L
*H. simulans*	L	L	L	L	L	-	-	L	-	-
*H. strumosus*	L	L	-	-	-	-	-	L	-	-
*H. tuberosus*	MS	MS	-	-	-	-	SC	SC	SC	MS
*H. heterophyllus*	-	-	-	-	-	L	L	-	-	L
*H. longifolius*	-	-	-	-	-	L	L	-	-	L
*H. atrorubens*	-	-	-	-	-	L	L	-	-	-
*H. glaucophyllus*	-	-	-	-	-	L	L	-	-	-
*H. gracilentus*	-	-	-	-	-	L	L	-	-	-
*H. carnosus*	-	-	-	-	-	-	-	-	L	L

Identification confidence codes: L = Literature-only correspond to those flavonoids reported in the literature), MS = MS tentative, SC = standard confirmed, - = not reported.

**Table 5:** Other compounds identified or isolated from *Helianthus* species.

Name	Structure	Type of Compound	Species	Reference
2-Naphthylalanine	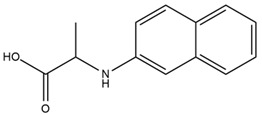	Alkaloid	*H. annuus*	[124]
Campesterol	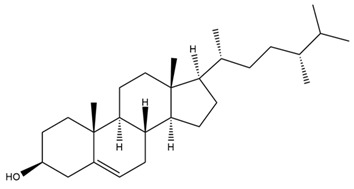	Phytosterol	*H. annuus*	[125]
Fenspiride	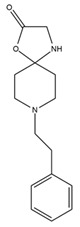	Alkaloid	*H. annuus*	[124]
Inulin	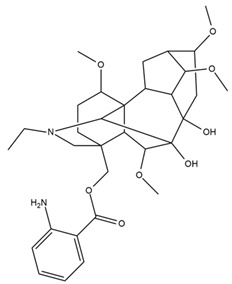	Polysaccharide	*H. tuberosus*	[127]
Linoleic acid	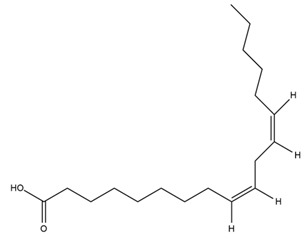	Unsaturated fatty acid	*H. annuus*	[126]
Medroxalol	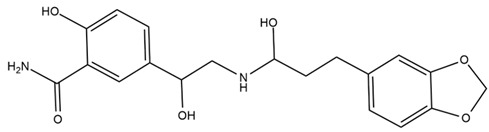	Alkaloid	*H. annuus*	[124]
Stigmasterol	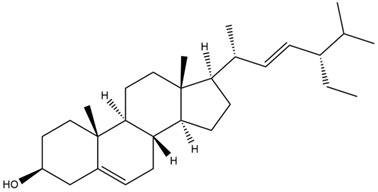	Phytosterol	*H. annuus*	[125]
α-tocopherol	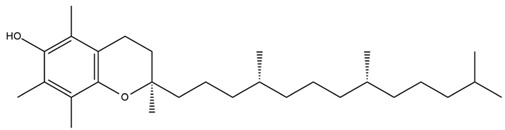	Tocopherol	*H. annuus*	[86]
β-sitosterol	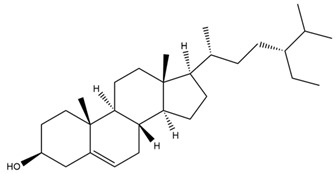	Phytosterol	*H. annuus*	[125]
β-tocopherol	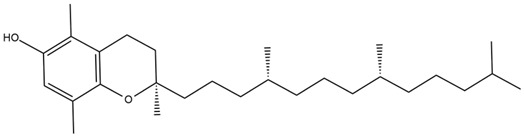	Tocopherol	*H. annuus*	[86]
γ-tocopherol	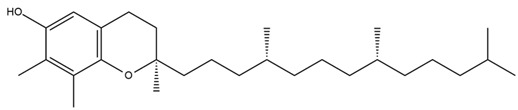	Tocopherol	*H. annuus*	[86]

## Data Availability

No new data were created in this study.

## Supplementary Materials

The following supporting information can be downloaded at: https://www.mdpi.com/article/10.3390/plants15030401/s1, Table S1: Uses of *Helianthus* species in different regions; Table S2: Antidiabetic and hepatoprotective activities reported for *Helianthus* species; Table S3: Analgesic, anti-inflammatory, antiulcer, antihistaminic, antidiarrheal, antifertility, antihyperuricemic, antiobesity, and Anti-urolithiatic activities reported for *Helianthus* species; Table S4: Antimicrobial and antiparasitic activities reported for *Helianthus* species; Table S5: Antioxidant activities reported for *Helianthus* species; Table S6: Anti-asthmatic effects, anti-atopic and anti-inflammatory activities, central nervous system modulation, cytotoxic and antiproliferative effects, photoprotective actions, and choleretic activity reported for *Helianthus* species; Table S7: Biological activities of terpenoid compounds isolated from *Helianthus* species; Table S8: Biological activities of flavonoids and phenolic compounds isolated from *Helianthus* species; Table S9: Biological activity of other compounds isolated from *Helianthus*.
