# Supplementary material for: Phytochemical Insights and Biological Potential of the Helianthus Genus
Source: Plants (Basel). 2026 Jan 28;15(3):401. doi: 10.3390/plants15030401 (PMC12899590; doi:10.3390/plants15030401)
Supplement: Supplementary file 1 [file plants-15-00401-s001.zip › plants-4046701-supplementary.pdf]

Phytochemical Insights and Biological Potential of the *Helianthus* Genus

Aldana Malen Corlatti [1,2], Hernán Bach [3,4], Ignacio Jorge Aguelo [5], Orlando Germán Elso [1,2], Rafael Ricco [5], Laura Cecilia Laurella [1,2,*] and Valeria Patricia Sülsen [1,2,*]

[1] Universidad de Buenos Aires, Facultad de Farmacia y Bioquímica, Cátedra de Farmacognosia, Junín 956 2° floor, Buenos Aires 1113, Argentina; aldanamalencorlattil@gmail.com (A.M.C.); orlando.elso@gmail.com (O.G.E.)
[2] CONICET–Universidad de Buenos Aires, Instituto de Química y Metabolismo del Fármaco (IQUIMEFA), Junín 956 2° floor, Buenos Aires 1113, Argentina
[3] Instituto Nacional de Tecnología Agropecuaria, Nicolas Repetto y De los Reseros s/n, Hurlingham; Buenos Aires B1686, Argentina; bach.hernan@inta.gob.ar
[4] Universidad de Buenos Aires, Facultad de Farmacia y Bioquímica, Museo de Farmacobotánica "Juan A. Dominguez", Junín 956 1° floor, Buenos Aires 1113, Argentina
[5] Universidad de Buenos Aires, Facultad de Farmacia y Bioquímica, Cátedra de Farmacobotánica, Junín 956 4° floor, Buenos Aires 1113, Argentina; iagudelo@ffyb.uba.ar (I.J.A.); raricco@ffyb.uba.ar (R.R.)
* Correspondence: vsulsen@ffyb.uba.ar (V.P.S.); c.laurella@docente.ffyb.uba.ar (L.C.L.); Tel.: +54-(011)-5287-4272 (V.P.S.)

Supplementary material

Table S1. Uses of *Helianthus* species in different regions

Species	Used part	Use/Indication	People group	Preparation	Geographic area	Reference
H. annuus	Aerial parts	Forage	Rural inhabitants	Not specified	Nuevo León, México	[26]
	Achenes	Asthma treatment	Romans	decoction mixed with J. rhombifolia	Northwestern Argentina	[28]
	Achenes	Hyperchloesterolemia, hypertension	Not specified	Not specified	Roma people, Brazil	[29]
	Achenes	Vermifuge	Not specified	Not specified	Bahía, Brazil	[30]
	Seeds	Strokes, headaches, toothaches and insect bites	Not specified	Alcoholic beverages	Pará Brazil	[31]
	Seeds	Strokes, headaches, toothaches and insect bites	Not specified	Infusions and decoctions	Northeastern Brazil	[32]
	Seeds	Strokes and labyrinthitis	Not specified	Infusions and decoctions	Ceará, Brazil	[33]
	Part not specified	Respiratory conditions	Riverside populations	Syrup	Matto Grosso, Brazil	[34]
	Achenes and flowering aerial parts	Digestive conditions	Not specified	Decoction	Boyacá, Colombia	[35]
	Part not specified	Food and ornamental purposes	Not specified	Not specified	Asturias, Spain	[36]
	Seeds	Cholesterol and circulatory pathologies	Not specified	Not specified	Morocco	[37]

	Seeds	Skin infections	Albanians and Gorani people	Mix with animal fat	Southern Kosovo	[38]
	Part not specified	Dermatitis and anti-stomachic	Albanians	Infusions and decoctions	Western Balkans	[39]
	Leaves	Respiratory infections	Not specified	Infusions and decoctions	Ordo, Nigeria	[40]
H. tuberosus	Tubers	Diabetes	Rural inhabitants	Eaten	Turkey	[42-45]
		Diabetes	Rural inhabitants	Eaten	Southeastern Serbia	[46]
		Constipation/Edible	Not specified	Decoction/Salad	Macedonia, Greece	[47]
		Diabetes	Rural inhabitants	Eaten	Lazio, Italia	[48]
		Diabetes	Not specified	Eaten	Emilia Romagna, Italia and Malta	[49]
		Galactagogue	Not specified	Eaten	Central Italy	[50]
		Edible	Not specified	Eaten	Slovenia	[51]
		Edible	Albanians	Eaten	Southern Kosovo	[38]
		Edible	Not specified	Eaten	Armenia	[52]
		Forage	Rural inhabitants	Eaten	Argentina	[53]
H. debilis	Sap	Wound healing	Not specified	Not specified	Uttarakhand region, India	[54]
H. x *laetiflorus*	Part not specified	Decorative purposes	Not specified	Not specified	Asturias, Spain	[36]

Table S2. Antidiabetic and hepatoprotective activities reported for *Helianthus* species

Species	Activity	Plant part	Extract	Assay/ model (evidence level)	Main result	Reference
H. annuus	Antidiabetic	Leaves	Ethyl acetate, hexane, acetone extracts	*In vitro* α-glucosidase and α-amylase inhibition assays (Level D)	Hexane extract highest inhibitory activity (α-glucosidase IC_{50}: 3.29 ± 0.12 mg/mL; α-amylase 3.92 ± 0.02 mg/mL)	[94]
H. annuus	Antidiabetic, antiglycative	Seeds	Ethanolic extract	*In vivo* rat models (normoglycemic, glucose-loaded, STZ-induced type 2 diabetes) (Level A)	Significantly reduced blood glucose in normoglycemic rats ($p < 0.05$); markedly stronger hypoglycemic effect and improved glucose tolerance in diabetic rats ($p < 0.01$)	[75]
H. annuus	Antidiabetic	Not specified	Crude methanolic extract and fractions	*In vivo* alloxan-induced hyperglycemic rats (Level A)	At 6 h, crude extract (600 mg/kg) reduced glucose by 66.74%; fractions 8, 9, 10, and 13 (60 mg/kg) reduced glucose by 59.80–78.03%, comparable or superior to glibenclamide	[75]

H. annuus	Antidiabetic	Leaves	Methanolic extract	*In vivo* alloxan-induced diabetic rats; oral glucose tolerance test (OGTT) (Level A)	Dose- and time-dependent glucose reduction ($p < 0.05$); 66.74% decrease at 600 mg/kg (6 h); improved OGTT responses comparable to glibenclamide; concentration-dependent antioxidant activity	[75]
H. annuus	Antiglycative	Sprouts	Not specified	*In vitro* AGE-formation inhibition (Level D)	Inhibited AGE formation by 83.29% at 1.0 mg/mL, exceeding aminoguanidine (80.88%)	[75]
H. annuus	Hepatoprotective	Flowers	Aqueous and ethanolic extracts	*In vivo* CCl_4-induced hepatotoxicity in Wistar rats (Level A)	Treatment (200 mg/kg) significantly reduced serum ALP, total bilirubin, GPT, and GOT levels ($p < 0.001$) and improved liver histopathology	[75]
H. annuus	Hypoglycemic, antidyslipidemic hepatoprotective	Leaves	Hydro methanolic extract	*In vivo* alloxan-induced diabetic rat model (Level A)	Oral administration (150, 300, 600 mg/kg/day, 21 days) significantly reduced fasting blood glucose, glycosylated hemoglobin, malondialdehyde, serum lipid parameters ($p < 0.05$), and improved body weight gain compared with the negative control (5% Tween-20). Histopathological showed reversal of alloxan-induced hepatic and pancreatic degeneration, with effects comparable to glibenclamide (2 mg/kg)	[95]
H. annuus	Hepatoprotective	Seeds	Methanolic extract	*In vivo* paracetamol-induced hepatotoxicity l in rats (Level A)	Oral administration (100, 300, 500 mg/kg/day, 7 days) significantly reduced serum hepatic enzymes (ALT, AST, ALP) after acetaminophen challenge ($p < 0.0001$); effect associated with high phenolic content (40.60 ± 1.14 mg GAE/g) and flavonoid content (7.72 ± 2.3 mg QE/g)	[96]
H. tuberosus	Antidiabetic	Tubers	Methanolic extract; *n*-hexane, methanolic and ethyl acetate fractions	*In vitro* α-amylase and α-glucosidase inhibition; insulin-resistant HepG2 glucose uptake (Level C)	The ethyl acetate fraction showed the strongest activity, inhibiting α-glucosidase (IC_{50} = 187.04 ± 0.42 μg/mL) and α-amylase (IC_{50} = 102.53 ± 1.39 μg/mL). It also enhanced glucose uptake in insulin-resistant HepG2 cells	[97]

Evidence level classification used: Level A: in vivo animal models or clinical data, Level B: ex vivo or complex in situ biological systems, Level C: in vitro cellular models, Level D: in vitro chemical or biochemical assays

Table S3. Analgesic, anti-inflammatory,antiulcer, antihistaminic, antidiarrheal, antifertility, antihyperuricemic, antiobesity, and Anti-urolithiatic activities reported for *Helianthus* species

Species	Activity	Plant part	Extract	Assay/ model (evidence level)	Main result	Reference
H. annuus	Analgesic	Seeds	Methanolic extract	*In vivo* acetic acid–induced writhing and hot plate tests in mice (Level A)	Inhibited nociceptive responses by 50.35% (100 mg/kg) and 57.85% (200 mg/kg) ($p < 0.05$) in the writhing test; increased reaction latency in the hot plate test (13.0 ± 0.91 s and 16.5 ± 1.55 s at 60 min), efficacy comparable to or greater than aspirin.	[75, 76]
H. annuus	Analgesic, anti-inflammatory	Leaves	Ethanolic extracts	*In vivo* rat models: tail immersion test; hot plate test; albumin-induced paw edema (Level A)	Treatment (0.5–4 g/kg or 150–600 mg/kg) produced dose-dependent analgesic and anti-inflammatory effects. Significant reductions in paw edema within 3 h (300 mg/kg), and nociceptive inhibition comparable to indomethacin, acetylsalicylic acid, and pentazocine in standard pain models.	[76,98]
H. annuus	Anti-ulcer	Leaves	Hydroalcoholic extract	*In vivo* Albino Wistar rat models: ethanol-induced gastric ulcer; pyloric ligation–induced gastric ulcer (Level A)	Administration (200 and 400 mg/kg) produced dose-dependent gastroprotection. In the ethanol-induced model, ulcer inhibition reached 57.79% and 62.41%; in the pyloric ligation model inhibition was 61.69% and 67.18%, respectively. Combination with *A. indicum* showed protection up to 75.23%, approaching omeprazole (20 mg/kg).	[99]
H. annuus	Antihistaminic	Leaves	Ethanolic extract	*In vivo* histamine-induced bronchoconstriction (microshock) models in guinea pigs (0.1% histamine aerosol) and rabbits (0.2% histamine aerosol) (Level A)	At 250 and 500 mg/kg, the extract afforded 46.54% and 62.15% protection in guinea pigs and 52.55% and 70.69% protection in rabbits, respectively ($p < 0.001$)	[76]

H. annuus	Antidiarrheal	Leaves	Ethanolic extract	*In vivo* castor oil–induced diarrhea and gastrointestinal transit models in mice (Level A)	At 250 and 500 mg/kg, fecal output was reduced by 42.15% and 67.01%, respectively ($p < 0.001$), while intestinal transit was decreased by 27.59% and 48.62% compared with controls	[76]
H. annuus	Anti-fertility / reproductive toxicity	Leaves	Ethanolic extract	*In vivo* Wistar rat reproductive models (fecundity assessment; epididymal sperm analysis; reproductive hormone levels; testicular histology) (Level A)	Oral administration at 0.5 g/kg/day for 14 days did not affect coital frequency but significantly reduced pregnancy rate and number of pups per rat. Treatment altered epididymal sperm parameters, circulating reproductive hormone levels, and induced histo-degenerative changes in the gonads, indicating impaired fertility in male reproductive function under the tested conditions	[75, 76]
H. annuus	Anti-gout, antihyperuricemic	Flower head	Sunflower head extract (SHEB; 20% ethanol: 80% water)	*In vivo* MSU-induced acute gout in Sprague–Dawley rats; induced hyperuricemia model in BALB/c mice (Level A)	Oral administration at 1 g/kg/day for 8 days significantly reduced ankle swelling (16.2% at 12 h; 27.1% at 48 h, $p < 0.05$), lowered serum uric acid (~50% reduction) and inhibited xanthine oxidase activity (13.1% in serum; 40.2% in liver, $p < 0.05–0.01$). Treatment improved joint histology by reducing inflammatory cell infiltration and increased anti-inflammatory IL-10 (35.5 ± 4.9 vs. 22.3 ± 3.0 pg/mL, $p < 0.001$), and reduced oxidative stress markers (MDA 66.5%, NO 59.1%, SOD 59.2%, GPx 65.2%).	[100]

H. annuus	Antiobesity, hypolipidemic	Seeds	Mathanolic extract	*In vivo* diet-induced obesity model in mice (cafeteria diet) (Level A)	At 200 mg/kg/day for 6 weeks significantly reduced body weight, BMI, LIO, food intake, blood glucose, triglycerides, total cholesterol, and LDL levels, while increased HDL levels and locomotor activity. Effects were comparable to atorvastatin (10 mg/kg).	[75, 76]
H. tuberosus	Antiobesity	Roots	Polyherbal formulations containing *H. tuberosus* root powder	*In vivo* diet-induced obesity model in mice (high-fat diet) (Level A)	Formulations significantly reduced body weight gain compared with the high-fat diet control group, improved lipoprotein profile and atherogenic index, and decreased liver and epididymal white adipose tissue weights, indicating attenuation of obesity-associated metabolic alterations in obese mice.	[101]
H. annuus	Anti-urolithiatic (anti-nephrolithiasis)	Leaves	Ethanolic and aqueous extract	*In vivo* calcium oxalate nephrolithiasis model in male rats (hyperoxaluria induced by ammonium chloride and ethylene glycol*)* (Level A)	Both extracts (500 mg/day for 10 days) significantly reduced renal deposition of stone-forming constituents, including calcium and phosphorus, compared with calculogenic control rats	[75]

Evidence level classification used: Level A: in vivo animal models or clinical data, Level B: ex vivo or complex in situ biological systems, Level C: in vitro cellular models, Level D: in vitro chemical or biochemical assays

Table S4. Antimicrobial and antiparasitic activities reported for *Helianthus* species

Species	Activity	Plant part	Extract	Assay/ model (evidence level)	Main result	Reference
H. annuus	Antibacterial, antifungal	Polen (bee pollen)	Ethanolic extracts (frozen, freeze-dried, dried)	*In vitro* agar well diffusion assay against Gram-positive and Gram-negative bacteria and filamentous fungi (Level D)	Antimicrobial activity is expressed as inhibition zones (mm). Freeze-dried extracts showed the strongest antibacterial activity against *Paenibacillus larvae* (2.73 ± 0.21 mm), *Brochothrix thermosphacta* (2.64 ± 0.15 mm), and *Enterococcus raffinosus* (2.57 ± 0.21 mm), with antifungal activity against *Aspergillus niger* (1.77 ± 0.15 mm)	[102]
H. annuus	Antibacterial	Flowers	Ethanolic and aqueous extracts	*In vitro* disc diffusion and tube dilution assays against multidrug-resistant bacteria (Level C)	Ethanolic extract inhibited MDR *Escherichia coli* (MIC = 4.0 mg/mL) and *Pseudomonas* spp. (MIC = 2.5 mg/mL). Aqueous extract showed limited or no activity against MDR strains.	[103]
H. annuus	Antibacterial and antifungal	Seeds	Methanolic extract	*In vitro* agar diffusion assays (bacteria) and antifungal susceptibility tests (Level D)	At 50 μg/mL, strong antibacterial activity against *Salmonella typhi* (1.5 cm inhibition zone), moderate activity against *Staphylococcus aureus* (1.2 cm) and *Vibrio cholerae* (1.1 cm), comparable to ampicillin (10 μg/mL). Strong antifungal activity against *Aspergillus fumigatus* (1.3 cm) and *Rhizopus stolonifer* (1.2 cm).	[75]
H. annuus	Antibacterial	Leaves	Aqueous and ethanolic extracts	*In vitro* disc diffusion and agar well diffusion assays (Level D)	Ethanolic extract exhibited stronger antibacterial activity than aqueous extract, producing inhibition zones of ~5.2–7.1 mm against *S. aureus*, *E. coli*, *P. aeruginosa*, *K. pneumoniae*, and *B. subtilis*, *S. typhimurium*, and *M. luteus*	[75]
H. annuus	Antibacterial and antifungal	Stem	Ethanolic extract (HMT)	*In vitro* MIC and MBC/MFC determination (Level C)	Antimicrobial activity observed against *S. aureus* (MIC/MBC = 70/90 mg/mL), *Candida albicans* (50/70 mg/mL), and *Aspergillus niger* (80/80 mg/mL). *E. coli* was resistant.	[75]
H. annuus	Antimicrobialdermatological	Seeds	Polar oil extract	*In vitro* antimicrobial assays; *in vivo* topical application in diaper dermatitis (Level A–C)	Seed oil inhibited *P. aeruginosa*, *S. epidermidis*, *S. aureus*, *C. albicans*, *P. vulgaris*, and *E. coli in vitro* and was effective as a topical agent in diaper dermatitis models.	[75]

H. annuus	Antimicrobial	Seeds	Sunflower seed oil	*In vivo* clinical study in preterm infants (<34 weeks gestation) (Level A)	Topical application (3× daily) significantly improved skin condition ($p = 0.037$) and reduced nosocomial infection incidence (adjusted incidence ratio = 0.46; 95% CI: 0.26–0.81; $p = 0.007$), with no adverse effects reported.	[75]
H. annuus	Antibacterial	Leaves	n-Hexane, methanol, ethyl acetate extracts	*In vitro* agar diffusion assay; MIC determination (Level C–D)	Methanolic extract inhibited all tested bacteria (*P. aeruginosa, S. aureus, K. pneumoniae, B. subtilis,* and *E. coli*) with MIC values ranging from ~1000–3000 mg/L. n-Hexane and ethyl acetate extracts showed selective activity, mainly against *P. aeruginosa*, *E. coli* and *B. subtilis*	[104]
H. salicifolius	Antibacterial	Aerial parts	Supercritical CO_2 extract (with water co-solvent)	*In vitro* MIC determination and time–kill assay against *S. aureus*; antioxidant EC_{50} assays (Level C–D)	Exhibited strong antistaphylococcal activity (MIC = 0.62 mg/mL) and antioxidant activity (EC_{50} = 0.609 mg/mL). Total polyphenol content: 13.75 ± 0.50 mg GAE/g.	[105]
H. tuberosus	Antibacterial	Aerial parts	Supercritical CO_2 extract (with water co-solvent)	*In vitro* MIC determination and time–kill assay against *S. aureus*; antioxidant EC_{50} assays (Level C–D)	Antistaphylococcal activity against *S. aureus* (MIC = 2.5 mg/mL) and strong antioxidant activity (EC_{50} = 0.332 mg/mL), correlated with high polyphenol content (33.06 ± 0.80 mg GAE/g).	[105]
H. annuus	Antimicrobial	Seeds	Phenolic-rich ethanolic and sodium bisulfite extracts	*In vitro* MIC determination against *S. aureus, E. coli, B. subtilis, and P. aeruginosa* (Level C)	Ethanolic extract showed highest activity, particularly against *E. coli* (MIC = 11.6 mg CGA/mL), followed by *B. subtilis* (26.5 mg CGA/mL), *S. aureus* (33.2 mg CGA/mL), and *P. aeruginosa* (33.2 mg CGA/mL). Sodium bisulfite extract showed lower potency.	[106]
H. annuus	Antibacterial	Leaves	Ethanolic extract ± lycopene (1 μM)	*In vitro* antibacterial assays (disc diffusion, MIC) (Level C)	Lycopene supplementation enhanced antibacterial activity against MDR *Streptococcus agalactiae* (MIC reduced from 175 to 80 μg) and *S. pyogenes* (125 to 50 μg)	[107]
H. annuus	Antileishmanial	Leaves	Chloroform and petroleum ether extracts	*In vitro* assay against *Leishmania donovani* promastigotes (Level C)	High antileishmanial potency observed (IC_{50} = 3.0 μg/mL for chloroform extract; 4.5 μg/mL for petroleum ether extract).	[108]

H. annuus	Antiplasmodial	Seeds	Methanolic and petroleum ether extracts	*In vitro* assay against *Plasmodium falciparum* K1 strain (Level C)	Exhibited notable antiplasmodial activity with IC_{50} values of 0.1 μg/mL (methanol) and 0.6 μg/mL (petroleum ether).	[75]
H. annuus	Antiplasmodial	Leaves	Ethanolic extract	*In vivo Plasmodium berghei*-infected Swiss albino mice (Level A)	Administration (2 and 4 g/kg/day, 3 days) produced very high chemosuppression rates (98.1% and 98.3%, respectively)	[75]
H. annuus	Antimalarial	Roots, leaves (also flowers, seeds, stems)	Ethanolic extract	*In vitro P. falciparum* 3D7 growth inhibition; heme detoxification inhibition; *in vivo P. berghei* BALB/c mouse models (Level A–C)	Root extract showed highest ***in vitro*** activity (IC_{50} = 2.3 ± 1.4 μg/mL), followed by leaf extract (IC_{50} = 4.3 ± 2.2 μg/mL). *In vivo*, root extract produced 63.6 ± 8.0% parasite inhibition at 100 mg/kg (suppressive test), 79.2% inhibition at 400 mg/kg (prophylactic test), and an ED_{50} of 10.6 ± 0.2 mg/kg (curative test). Both root and leaf extracts inhibited heme detoxification more effectively than chloroquine (IC_{50} = 0.4 ± 0.0 and 0.5 ± 0.0 mg/mL, respectively).	[109]

Evidence level classification used: Level A: in vivo animal models or clinical data, Level B: ex vivo or complex in situ biological systems, Level C: in vitro cellular models, Level D: in vitro chemical or biochemical assays

Table S5. Antioxidant activities reported for *Helianthus* species

Species	Activity	Plant part	Extract	Assay/ model (evidence level)	Main result	Reference
H. annuus	Antioxidant	Cotyledons	Aqueous and ethanolic extracts (sequential extraction)	*In vitro* chemical antioxidant assays: DPPH, ORAC, FRAP assays (Level D)	The aqueous extract (30 µg/mL) exhibited higher antioxidant activity than the ethanolic extract across all assays (DPPH inhibition 50.18%; ORAC 1.5 Trolox equivalents; FRAP 45.27 µmol), corresponding to ~45–66% of the activity of BHT depending on the assay	[110]
H. annuus	Antioxidant	Bee pollen	Ethanolic extracts (frozen, dried, freeze-dried)	*In vitro* DPPH radical scavenging and phosphomolybdenum reducing power assays (Level D)	All extracts showed moderate antioxidant activity (~48–50% DPPH inhibition). The freeze-dried pollen extract displayed the highest activity, which correlated positively with total polyphenol content	[102]
H. annuus	Antioxidant	Leaves	Methanolic extract and fractions	*In vitro* DPPH radical scavenging assay (Level D)	The crude methanolic extract showed moderate activity (55 ± 0.05% inhibition at 500 µg/mL). Fraction 7 exhibited the highest activity (65% inhibition; IC_{50} = 329 µg/mL)	[111]
H. annuus	Antioxidant	Leaves	Methanolic extract and fractions	*In vitro* DPPH and FRAP assays (Level D)	The crude methanolic extract and fractions 8, 9, 10, and 13 showed notable antioxidant activity at 400 µg/mL. Fraction 13 was the most active (DPPH inhibition 92.72%; FRAP 0.28 µM)	[75]
H. annuus	Antioxidant	Seeds	Methanolic extract	*In vitro* DPPH radical scavenging assay (Level D)	The methanolic seed extract exhibited strong, concentration-dependent DPPH radical scavenging activity, consistent with the presence of phenolic antioxidants	[75]
H. annuus	Antioxidant	Sprouts	80% methanolic extract	*In vitro* DPPH, ferric reducing power, and β-carotene oxidation assays (Level D)	The extract showed strong antioxidant activity with EC_{50} values of 35.00 ± 3.82 µg/mL (DPPH), 18.00 ± 0.31 µg/mL (Fe^{3+} reduction), and 2.93 ± 0.23 µg/mL (β-carotene oxidation protection)	[75]

H. annuus	Antioxidant	Leaves, flowers, stem bark	Ethanolic extract	*In vitro* DPPH radical scavenging assay (Level D)	Leaf extract showed the strongest activity (IC_{50} = 48.84 ppm), followed by flower (IC_{50} = 180.50 ppm), while stem bark exhibited weak activity (IC_{50} = 274.03 ppm). Leaf extract activity correlated with total phenolic (35.15 mg GAE/g extract) and flavonoid content (10.92 mg QE/g extract).	[112]
H. annuus	Antioxidant	Seeds	Phenolic-rich ethanolic and sodium bisulfite extracts	*In vitro* DPPH radical scavenging assay (Level D)	The ethanolic extract showed greater antioxidant activity than the sodium bisulfite extract (EC_{50} = 0.36 vs. 1.01 g extract/g DPPH), confirming ethanol as a more efficient solvent for extracting antioxidant phenolics	[106]
H. annuus	Antioxidant	Leaves	Ethanolic extract enriched with lycopene	*In vitro* DPPH assay and cellular ROS model in human fibroblasts (Level C–D)	Lycopene supplementation enhanced antioxidant activity, increasing DPPH scavenging by 30% and reducing intracellular ROS levels by 24% compared with the extract alone, indicating a synergistic interaction	[107]
H. tuberosus	Antioxidant	Root	Aqueous–methanolic extract	*In vitro* human plasma oxidative stress model (H_2O_2/Fe^{2+}–induced); TBARS, protein carbonylation, thiol oxidation; ORAC; TAC; TLC–DPPH• (Level C–D)	At 50 µg/mL, the extract reduced plasma protein carbonylation by ~50%, inhibited thiol oxidation, and significantly modulated lipid peroxidation (TBARS). No significant effects on ORAC or TAC were observed. Moderate radical scavenging activity was detected by TLC–DPPH• (0.140 ± 0.01 relative to chlorogenic acid)	[113]
H. tuberosus	Antioxidant	Tuber	Methanolic extract and fractions (n-hexane, ethyl acetate)	*In vitro* DPPH and ABTS radical scavenging assays (Level D)	The ethyl acetate fraction exhibited the strongest antioxidant activity (IC_{50} = 161.55 ± 0.98 µg/mL for DPPH; 104.45 ± 3.01 µg/mL for ABTS), outperforming the crude extract and other fractions	[97]

Evidence level classification used: Level A: in vivo animal models or clinical data, Level B: ex vivo or complex in situ biological systems, Level C: in vitro cellular models, Level D: in vitro chemical or biochemical assays

Table S6. Anti-asthmatic effects, anti-atopic and anti-inflammatory activities, central nervous system modulation, cytotoxic and antiproliferative effects, photoprotective actions, and choleretic activity reported for *Helianthus* species

Species	Activity	Plant part	Extract	Assay/ model (evidence level)	Main result	Reference
H. annuus	Anti-asthmatic, immunomodulatory	Seeds	Aqueous extract	*In vivo* ovalbumin (OVA)-induced murine asthma model; lung histopathology, immunohistochemistry, ELISA (IL-4, IL-13, IgE) (Level A)	Treatment attenuated OVA-induced asthma features in mice, reducing pulmonary inflammatory cell infiltration and $CD4^+$ T-cell accumulation. Th2 cytokines were markedly downregulated (IL-4 reduced to ~42% and IL-13 to ~30% of OVA-induced levels), with a significant decrease in serum IgE. No morphological toxicity was observed at 6–60 g/kg/day	[114]
H. tuberosus	Anti-atopic, anti-inflammatory	Tubers	30% ethanolic extract	*In vivo* NC/Nga mouse model of atopic dermatitis induced with *Dermatophagoides farinae body (Dfb)* topical treatment; *In vitro* TNF-α/IFN-γ-stimulated HaCaT keratinocytes (Level A–B)	Topical treatment (100 mg/kg/day, 4 weeks) significantly reduced dermatitis score ($p < 0.001$), epidermal thickness, mast cell infiltration, serum IgE and histamine levels; restored filaggrin expression; downregulated ICAM-1, VCAM-1 and E-selectin. Effects were associated with inhibition of NF-κB, Akt and MAPK (ERK, JNK, p38) signaling pathways; effects comparable to or greater than dexamethasone	[115]
H. annuus	CNS activity (stimulant, anxiolytic, antidepressant)	Seeds	Methanolic extract	*In vivo* behavioral assays in mice (light–dark box, elevated plus maze, tail suspension test) (Level A)	At 100–200 mg/kg, the extract induced significant CNS-stimulant effects (increased spontaneous activity, grip strength, pain response, and motor reflexes), moderate anxiolytic activity with increased latency to enter the dark compartment (34 ± 5.63 s in controls vs. 63 ± 0.62 s and 72 ± 0.85 s at 100 and 200 mg/kg, respectively) and increased time spent in open arms in the elevated plus maze. Marked antidepressant-like effects by reduced immobility time (190.8 ± 0.75 s in controls vs. 93 ± 0.47 s and 78 ± 1.3 s at 100 and 200 mg/kg; $p < 0.05$) in the tail suspension test. No neurotoxicity was observed.	[116]

H. angustifolius	Cytotoxic (anticancer)	Flowers	Dichloromethane extract	*In vitro* cytotoxicity assay on CCRF-CEM leukemia cells (Level D)	Potent cytotoxic activity, reducing CCRF-CEM cell viability to ~0.2% of control at 10 μg/mL, indicating strong antiproliferative activity	[61]
H. annuus	Antiproliferative (cytotoxic)	Roots	Chloroform extract	*In vitro* antiproliferative assays on human cancer cell lines (MCF-7, A-431, HeLa) (Level D)	Marked cytotoxic effects, MIC_{50} =3.36 μg/mL (MCF-7), 4.19 μg/mL (A-431), and 3.51 μg/mL (HeLa), demonstrating strong antiproliferative effects at low concentrations	[75, 76]
H. annuus	Chemopreventive	Seeds	Phenolic-rich ethanolic and sodium bisulfite extracts	*In vitro* antiproliferative assay and plasmid DNA oxidative damage model (AAPH-induced) (Level D)	No direct antiproliferative effect observed at 0.25–250 μg/mL; however, both extracts protected DNA from oxidative damage. The ethanolic extract showed superior DNA protection (89% DNA retention vs. 52% the bisulfite extract), suggesting chemopreventive potential via antioxidant-mediated DNA protection	[106]
H. annuus	Photoprotective	Flowers	Ethanolic extract	*In vitro* UVB-induced photoaging model in human dermal fibroblasts; DPPH and ABTS assays (Level C–D)	The extract reduced UVB-induced ROS generation (UVB irradiation 144 mJ/cm^2); inhibited MMP-1 and MMP-3 expression; preserved procollagen I; and suppressed inflammatory mediators (activated Nrf2 nuclear translocation and upregulated TGF-β1; inhibited MAPK/AP-1 signaling; suppressed VEGF, IL-6, COX-2, iNOS, and TNF-α production). Antioxidant activity was confirmed (IC_{50} = 72.65 μg/mL, DPPH; 174.52 μg/mL, ABTS)	[117]
H. annuus	Choleretic and cholekinetic activity	Sunflower heads	Dry extract (DESH)	*In vivo* CCl_4 + ethanol–induced liver injury model in rats (Level A)	DESH significantly increased bile secretion rate by 1.7-fold (75 mg/kg) and 1.5-fold (100 mg/kg) ($p < 0.05$); normalized bile acid synthesis (1.4-fold increase, $p < 0.05$) and improved biliary cholesterol content (1.7-fold at 75 mg/kg; 1.5-fold at 100 mg/kg). Effective doses ranged from 75–100 mg/kg, indicating pronounced choleretic activity	[118]

Evidence level classification used: Level A: in vivo animal models or clinical data, Level B: ex vivo or complex in situ biological systems, Level C: in vitro cellular models, Level D: in vitro chemical or biochemical assays

Table S7. Biological activities of terpenoid compounds isolated from *Helianthus* species

Species	Activity	Compound	Type of natural terpenoid	Plant source	Assay/ model (evidence level)	Main result	Reference
H. annuus	Anti-inflammatory	Kaurenoic acid; Trachylobanoic acid; Grandifloric acid	Diterpene acids	Flower heads (petroleum ether extract)	*In vitro* LPS-stimulated RAW 264.7 macrophages (NO, PGE_2, TNF-α, NOS-2, COX-2); *In vivo* TPA-induced mouse ear edema (Level C-B)	Concentration-dependent inhibition of NO, PGE_2 and TNF-α (1–20 μM); downregulation of NOS-2 and COX-2; significant reduction of ear edema and MPO activity, comparable to indomethacin	[75, 76]
H. annuus	Anti-inflammatory	Helianthosides 1, 2, 3, 4, 5; Helianthoside B	Triterpene glycosides	Flower petals (MeOH extract, n-BuOH fraction)	*In vivo* TPA-induced mouse ear edema model (Level B)	Strong inhibition of ear edema (ID_{50}: 65–262 nmol/ear); higher potency than indomethacin; helianthoside B comparable to hydrocortisone	[75, 76]
H. tuberosus	Anti-inflammatory, anti-atherosclerotic (cellular)	Heliangin	Sesquiterpene lactone	Leaves (ethanolic extract)	*In vitro* LPS-stimulated RAW 264.7 macrophages; TNF-α–stimulated vascular endothelial cells (Level C)	Reduced NO production (2–25 μM); suppressed ICAM-1, VCAM-1, E-selectin and MCP-1 via inhibition of NF-κB/IκBα phosphorylation	[65]
H. annuus	Antiviral, (EBV)	Sunpollenol; (24R)-24,25 Epoxysunpollenol; (24S)-24,25-Epoxysunpollenol; (23E)-23-Dehydro-25-hydroxysunpollenol; (24R)-24,25-Dihydroxysunpollenol; (24S)-24,25-Dihydroxysunpollenol.	3,4-seco-tirucallane-type triterpenoids	Pollen grains (diethyl ether extract)	*In vitro* EBV-EA activation induced by TPA in Raji cells (Level C)	Potent inhibition of EBV-EA activation (97–100% inhibition at 1×10^3 mol ratio/TPA) without cytotoxicity	[119]

H. tuberosus	Antiparasitic	4,15-iso-atriplicolide tiglate	Sesquiterpene lactone	Aerial parts (CH_2Cl_2 extract)	*In vitro* against *Trypanosoma brucei rhodesiense*, *T. cruzi*, *P. falciparum* (Level C)	Strong antitrypanosomal activity (IC_{50} = 0.015 μM); moderate activity against *T. cruzi* (3.7 μM) and *P. falciparum* (1.0 μM)	[64]
H. tuberosus	Cytotoxic	4,15-iso-atriplicolide tiglate	Sesquiterpene lactone	Leaves (EtOAc extract)	*In vitro* MTT assay on MCF-7, A549, HeLa cells (Level C)	Significant growth inhibitory activity (0.1–20 μg/mL) against MCF-7, A549, and HeLa cancer cells lines with IC_{50} values of 1.97 ± 0.04, 7.79 ± 0.44, and 9.87 ± mg/ml, respectively	[62]
H. angustifolius	Cytotoxic	8-Methacrylyl-4,15-iso-atriplicolide	Sesquiterpene lactone	Flowers (CH_2Cl_2 extract)	*In vitro* XTT viability assay against human cancer cell lines (CCRF-CEM leukemia, MDA-MB-231 breast cancer, U251 glioblastoma, and HCT 116 colon cancer cells),and human lung fibroblast cell line MRC-5 (Level C)	High potency against CCRF-CEM (IC_{50} = 0.26 ±0.01 μM), IC_{50} =3.08±0.15 μM for MDA-MB-231 cells, 10.17±1.60 μM for U251 cells, 1.02±0.09 μM for HCT 116 cells; selective cytotoxicity vs. MRC-5 (IC_{50} = 4.22 ± 0.26 μM)	[61]
H. annuus	Antioxidant, antidiabetic	20-Dehydroeucannabinolide	Sesquiterpene lactone	Leaves (hydromethanolic extract)	*In vitro* DPPH and NO scavenging; *in vivo* alloxan-induced diabetic rats (Level A–D)	Moderate radical scavenging (23.7–26.0% at 954.2 μM for DPPH and NO radical scavenging assays); significant ($p < 0.05$) reduction of fasting blood glucose comparable to glibenclamide	[121]

H. debilis subsp. cucumerifolius	Antimicrobial	17,18-Dihydrobudlein A	Sesquiterpene lactone	Leaves (acetone extract)	*In vitro* Agar diffusion assay against *Bacillus brevis* (Level C)	Strong antibacterial activity (MIC = 16 ng/mL)	[22]
H. annuus	Antimicrobial	Glandulon A, B, C	Sesquiterpenes	Not specified	*In vitro* Agar diffusion assay against *Bacillus brevis* (Level C)	Cytostatic antibacterial effect	[22]

Evidence level classification used: Level A: in vivo animal models or clinical data, Level B: ex vivo or complex in situ biological systems, Level C: in vitro cellular models, Level D: in vitro chemical or biochemical assays

Table S8. Biological activities of flavonoids and phenolic compounds isolated from *Helianthus* species

Species	Activity	Compound	Type of natural compound	Plant source	Assay/ model (evidence level)	Main result	Reference
H. tuberosus	Antioxidant	3-O-caffeolquinic acid	Phenolic acid	Leaves (60% ethanol extract, petroleum ether, ethyl acetate, n-butanol, and aqueous fractions)	*In vitro*: DPPH, ABTS$^+$, hydroxyl radical scavenging assays (Level D)	Strong radical scavenging activity; major contributor to antioxidant capacity due to caffeoyl moiety	[122]
H. tuberosus	Antioxidant	1,5-Dicaffeoylquinic acid	Phenolic acid	Leaves (60% ethanol extract, petroleum ether, ethyl acetate, n-butanol, and aqueous fractions)	*In vitro*: DPPH, ABTS$^+$, hydroxyl radical scavenging assays (Level D)	Higher antioxidant activity than monocaffeoyl derivatives; enhanced activity associated with two caffeoyl groups	[122]
H. annuus	Antioxidant	Chlorogenic acid	Phenolic acid	Defatted seeds flour	*In vitro:* DPPH, ABTS, ORAC assays (Level D)	Main contributor to antioxidant activity; accounts for ~62% of total phenolics; strong radical scavenging capacity	[106]

H. tuberosus	Antioxidant	5,8-diOH-6,7-diMeO-2-(3,4-diMeOPh)-4-benzopyrone	Flavonoid	Leaves	*In vitro*: DPPH●, $ABTS^+$, hydroxyl radical scavenging assays (Level D)	Dose-dependent radical scavenging with superior $ABTS^+$ and hydroxyl radical scavenging compared to BHT. The SC_{50} value in the DPPH assay was lower (90.61 ± 0.59 μg/mL) than that of BHT (103.30 ± 0.76 μg/mL), with significantly higher potency in the $ABTS^+$ assay (SC_{50} = 1.40 ± 0.06 μg/mL vs. 5.13 ± 0.21 μg/mL for BHT). Enhanced hydroxyl radical scavenging was also observed (SC_{50} = 15.07 ± 0.56 μg/mL vs. 46.62 ± 0.35 μg/mL for BHT).	[123]
H. tuberosus	Antioxidant	5,8-diOH-6,7,4′-triOMe (pedunculin)	Flavonoid	Leaves	*In vitro*: DPPH●, $ABTS^+$, hydroxyl radical scavenging assays (Level D)	Stronger radical scavenging activity than BHT, In the DPPH assay, the SC_{50} value (106.80 ± 0.85 μg/mL) was comparable to that of BHT (103.30 ± 0.76 μg/mL). In contrast, markedly superior activity was observed in the $ABTS^+$ assay (SC_{50} = 1.31 ± 0.11 μg/mL vs. 5.13 ± 0.21 μg/mL for BHT), along with enhanced hydroxyl radical scavenging capacity (SC_{50} = 10.61 ± 0.31 μg/mL vs. 46.62 ± 0.35 μg/mL for BHT).	[123]
H. annuus	Anti-obesity	Chlorogenic acids (including 3-, 4-, 5-caffeoylquinic acids, as well as 3,4- 3,5-, and 4,5-di-caffeoylquinic acids)	Phenolic acids	Seeds (40% chlorogenic acids standardized extract	*In vivo*: Randomized, double-blind, placebo-controlled clinical trial (Level A)	Reduction in body weight of 6.8% in the sunflower extract group versus 5.7% in the placebo group. Sunflower extract supplementation for 12 weeks resulted in a significantly greater reduction in BMI (−2.60 vs. −1.88; p = 0.02) and waist circumference (−8.44 vs. −4.75 cm; p = 0.001) compared with placebo, with more pronounced effects in women >30 years	[87]

Evidence level classification used: Level A: in vivo animal models or clinical data, Level B: ex vivo or complex in situ biological systems, Level C: in vitro cellular models, Level D: in vitro chemical or biochemical assays

Table S9. Biological activity of other compounds isolated from *Helianthus*

Species	Activity	Compound	Type of natural compound	Plant source	Assay/ model (evidence level)	Main result	Reference
H. annuus	Anti-gout	2-naphthylalanine, medroxalol, and fenspiride	Alkaloid	Receptacles	*In silico* (LC-MS profiling, molecular docking, molecular dynamics, MM-PBSA, computational clustering)	Strong predicted xanthine oxidase inhibitory activity; medroxalol showed higher predicted affinity than allopurinol.	[124]
H. annuus	Antioxidant	alpha-tocopherol, beta-tocopherol, gamma-tocopherol	Tocopherol	Seed oil	*In vitro* chemical and biochemical antioxidant assays (Level D)	α-Tocopherol shows the highest antioxidant efficacy in vivo, while its relative activity differs in vitro compared to other tocopherols due to distinct chemical interactions with lipid peroxidation	[86]
H. annuus	Neuroprotective; NGF-enhancing activity	β-sitosterol, stigmasterol and campesterol	Phytosterols (sterols)	Seeds	*In vitro* PC12 cell neurite outgrowth assay (Level C)	Sunflower seed extract promoted neurite outgrowth via NGF-enhancing activity. Bioassay-guided purification identified β-sitosterol as the main active compound; β-sitosterol and stigmasterol showed the strongest neurite outgrowth-promoting effects, associated with increased neurofilament expression.	[125]

H. tuberosus	Antihyperglycaemic; metabolic regulation	Inulin	Polysaccharide	Tubers	*In vivo* mouse model of hyperglycaemia (HFD + STZ) (Level A)	Inulin reduced fasting blood glucose, body weight, lipid parameters and liver weight, modulated liver gene expression, and improved intestinal microbiota composition, notably increasing Bacteroides.	[127]

Evidence level classification used: Level A: in vivo animal models or clinical data, Level B: ex vivo or complex in situ biological systems, Level C: in vitro cellular models, Level D: in vitro chemical or biochemical assays
